# Inflammatory cytokine alterations in genetic and clinical high risk groups of psychosis: a systematic review and network meta-analysis

**DOI:** 10.1017/S0033291726103559

**Published:** 2026-04-10

**Authors:** Huiqun Huang, Yuqi Sun, Jingyu Yin, Siyao An, Muchen Liu, Qiyong Gong, Ying Chen, Hong Deng

**Affiliations:** 1 Mental Health Center, West China Hospital of Sichuan University, Chengdu, Sichuan, China; 2 Department of Radiology and Huaxi MR Research Center (HMRRC), Functional and Molecular Imaging Key Laboratory of Sichuan Province, West China Hospital, Sichuan University, Chengdu, Sichuan, China; 3 Department of Developmental Psychology and Socialisation, University of Padova, Via Venezia, 8, 35131, Padova, Italy; 4 Law School of Southwest Minzu University, Chengdu, Sichuan, China; 5 Research Unit of Psychoradiology, Chinese Academy of Medical Sciences, Chengdu, Sichuan, China; 6 Xiamen Key Lab of Psychoradiology and Neuromodulation, Department of Radiology, West China Xiamen Hospital of Sichuan University, Xiamen, Fujian, China; 7 Mental Health Center, West China Xiamen Hospital of Sichuan University, Xiamen, Fujian, China

**Keywords:** cytokines, clinical high risk, genetic high risk, psychosis, network meta-analysis

## Abstract

**Background:**

Inflammation has been implicated in psychosis, but its role in individuals at clinical (CHR) and genetic (GHR) high-risk remains unclear. We therefore conducted a network meta-analysis (NMA) to compare circulating cytokine levels across CHR, GHR, and healthy control (HC) groups.

**Methods:**

We systematically searched multiple databases up to February 2025, extracting cytokine levels (plasma/serum) from CHR, GHR, and HC groups. Standardized mean differences (SMDs) with 95% confidence intervals (CIs) were estimated using random-effects models. Given that no direct head-to-head comparisons between CHR and GHR were available, indirect comparisons were performed through the common comparator (HC). The transitivity assumption was assessed by comparing key study and participant characteristics across comparisons.

**Results:**

Thirty studies were included (CHR: 1601, GHR: 675, HC: 1980). NMA estimates indicated higher IL-6 levels in CHR compared with GHR, while IL-6 and IL-1β levels were lower in GHR compared with HC. In pairwise subgroup analyses, CHR converters showed higher IL-13 levels than non-converters. The evidence network was sparse and star-shaped, with all CHR–GHR estimates relying exclusively on indirect comparisons.

**Conclusions:**

This study represents the first NMA to synthesize cytokine alterations in individuals at high risk for psychosis using indirect evidence. Elevated IL-6 in CHR individuals suggests immune activation, whereas reduced IL-6 in GHR may reflect a distinct immune profile. Increased IL-13 levels in converters highlight potential involvement of Th2-related pathways during transition to psychosis. However, the sparse nature of the evidence network necessitates cautious interpretation of the findings, and larger, standardized multi-center studies are required for confirmation.

## Introduction

Accumulating evidence indicates that dysregulation of the immune-inflammatory system plays a significant role in the pathophysiology of psychosis (Halstead et al., 2023). Studies demonstrate significantly elevated levels of pro-inflammatory pathway cytokines – – including C-reactive protein (CRP), tumor necrosis factor-α (TNF-α), interferon-γ (IFN-γ), interleukin-1β (IL-1β), and IL-6 – in patients with first-episode psychosis (FEP) (Roomruangwong et al., 2020). Notably, aberrant cytokine expression has also been observed in genetic high-risk individuals (GHR, first-degree relatives of psychosis patients with genetic susceptibility but no clinical symptoms) and clinical high-risk individuals (CHR, presenting with subthreshold psychotic symptoms) (Fu et al., 2025). This suggests that immune system dysregulation precedes the onset of psychosis, indicating that inflammatory imbalance may be a potential driver of disease development and progression.

CHR and GHR represent two distinct risk constructs in the field of psychiatry. The former is primarily defined by the presence of sub-threshold clinical symptoms, whereas the latter is defined through genetic susceptibility (Niznikiewicz, 2019). This fundamental difference in definition suggests that the underlying pathophysiological processes, including immune mechanisms, may differ between the two.

For CHR individuals, environmental factors such as childhood trauma, can activate the sympathetic nervous system and the hypothalamic–pituitary–adrenal axis, inducing peripheral inflammatory responses (Miller et al., 2024). According to the neuroimmune network model (Nusslock & Miller, 2015), these inflammatory substances can enter the brain through multiple pathways – such as leaky areas like circumventricular organs, specific transporter proteins, and neural signal transduction – damaging the blood–brain barrier, activating microglia to trigger neuroinflammation, and disrupting the functional balance of the dopamine and glutamate systems, ultimately leading to the emergence of subthreshold symptoms (Paul et al., 2024).

Conversely, among GHR individuals, inflammatory manifestations more closely resemble an innate, constitutionally set trait determined by genetic background, rather than a mere reaction to external stressors (Iakunchykova, Leonardsen, & Wang, 2024). Their immune system may be in a baseline state of mild dysregulation from early development, manifesting as congenital immune regulatory abnormalities, such as differences in microglial functional states (Howes & McCutcheon, 2017). This ‘developmental immune dysregulation’ may interfere with the normal pruning and shaping of neural circuits during critical periods of brain development, such as adolescence (Howes & Onwordi, 2023). For example, specific risk genes they carry (e.g., C4A variants in the MHC region) may render the brain more sensitive to subtle inflammatory signals during development (Sekar et al., 2016; Yilmaz et al., 2020). This intrinsic immune-neurodevelopmental interaction forms the unique risk basis for GHR individuals, keeping the nervous system in a potentially vulnerable state even in the absence of significant environmental stressors (Sellgren et al., 2019).

Collectively, the inflammatory dysregulation in CHR is more ‘stress-driven’, acting as a mediating mechanism between the environment and the brain, whereas the inflammatory characteristics in GHR tend to be ‘developmentally set’, representing an early manifestation of their genetic traits at the neuroimmune level. Moreover, CHR individuals who also possess genetic susceptibility exhibit a higher risk of disease conversion compared to pure GHR populations (Smieskova et al., 2013). Consequently, comparing inflammatory cytokine changes between these two high-risk groups can help elucidate the underlying immunopathological mechanisms associated with their respective risk origins.

Nevertheless, cytokine studies focusing on GHR and CHR individuals remain relatively limited, with existing results being fragmented and inconsistent. Some studies report elevated pro-inflammatory cytokines (e.g., IL-6, TNF-α) in CHR individuals, while GHR research shows mixed results – including elevations, reductions, or no significant changes (Corsi-Zuelli et al., 2020; Karanikas et al., 2017; Lizano et al., 2016; Stojanovic et al., 2014; Zeni-Graiff et al., 2016). To date, only the meta-analysis by Misiak et al. has compared peripheral blood inflammatory markers across different risk groups (Misiak et al., 2021). Their pairwise comparisons revealed significantly higher IL-6 levels in CHR individuals compared to healthy controls (HC), but no significant difference between GHR and HC. However, traditional meta-analysis methods are inadequate for directly comparing the similarities and differences in inflammatory profiles between GHR and CHR populations.

To address this gap, this study employs a network meta-analysis (NMA) approach to integrate direct and indirect evidence. It aims to systematically compare differences in peripheral blood inflammatory cytokine levels between CHR and GHR populations, with a focus on exploring whether risk type (clinically phenotype-driven vs. genetically driven) is associated with specific patterns of inflammatory dysregulation. Where data permit, it will also analyze differences in inflammatory cytokines between CHR converters (CHR-T) and non-converters (CHR-NT) to identify potential immune biomarkers associated with disease conversion.

## Methods

### Study selection and selection criteria

We searched PubMed, Web of Science, Cochrane, Embase, and MEDLINE from inception to February 18, 2025, using keywords related to psychosis risk and inflammatory markers (Supplementary Table S1). Additionally, references of eligible publications (Halstead et al., 2023; Misiak et al., 2021; Park & Miller, 2020) were checked for any additional relevant studies. This meta-analysis was registered on PROSPERO (CRD 42023479829).

The inclusion criteria were: 1) studies comparing peripheral cytokine levels between either CHR individuals (assessed using validated clinical instruments) or GHR individuals (unaffected first-degree relatives) and healthy controls; 2) inclusion of a healthy control group; 3) availability of necessary quantitative data; 4) publication as a peer-reviewed original article in English. Exclusion criteria were: 1) absence of a healthy control group; 2) insufficient data on cytokine levels (mean ± SD or median [IQR]) after author contact; 3) overlapping study populations; 4) non‑original research (e.g., reviews, animal or genetic studies, case reports).

**Figure 1.**
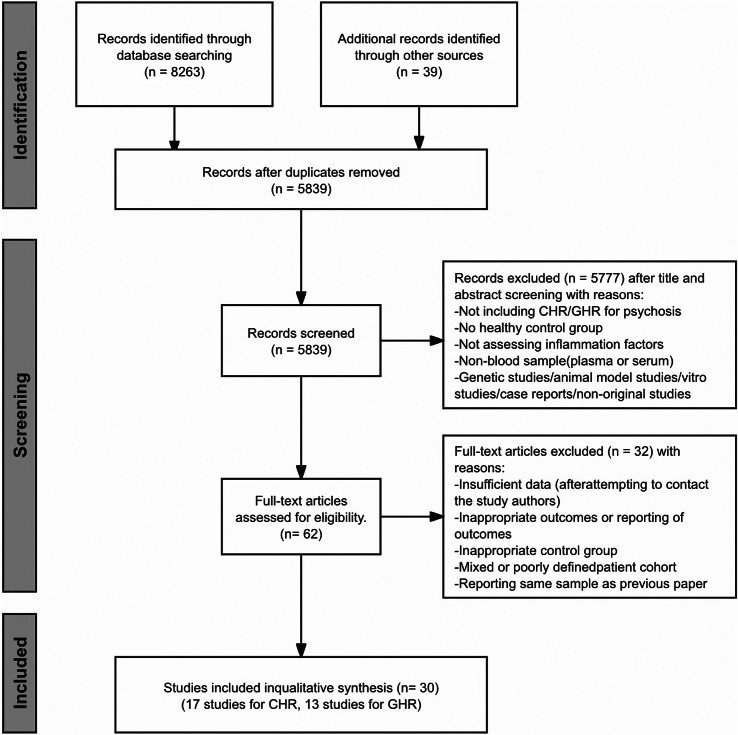
Flow chart of the study selection process. Abbreviations: CHR=clinical high risk; GHR=genetic high risk.

After duplicate removal, two authors (JYY and HQH) independently screened titles, abstracts, and full texts of all candidate studies. Any disagreements regarding study eligibility were resolved by consensus with a third reviewer (YC). Of 5839 initially identified records, 30 studies were ultimately included in this meta-analysis (17 CHR studies and 13 GHR studies). The study selection process is summarized in the flow chart (Figure 1).

### Quality assessment

Quality assessment was done via an adapted scale based on the Joanna Briggs Institute(JBI) critical appraisal tool for case–control studies (Glaría, Fernández, Salgado, & Hernández-Leal, 2024), similarly, to (Halstead et al. (2023), including seven criteria (Group Comparability, Matching, Criteria Consistency, Confounders Identified, Strategies for Confounding, Outcome Assessment) (Supplementary Table S3). For each criterion, articles were scored as ‘Yes’ (criteria met), ‘No’ (criteria not met), or ‘Unclear’ (insufficient evidence). Total quality scores ranged from 0 to 7, with higher scores indicating higher study quality. Scores from 0 to 2, 3 to 5, and 6 to 7 were classified as low, moderate, and high quality, respectively. A ‘quality index’ (total score/7) was derived for each study and used in meta-regression to examine potential quality-related bias (Supplementary Table S5).

### Data extraction

Three reviewers (JYY, YQS, HQH) independently screened and extracted data using the Cochrane tool (Föcking et al., 2016), with disagreements resolved through discussion or by a fourth reviewer (YC). The outcomes included peripheral concentrations of inflammatory proteins listed in Supplementary Table S2. Means and SDs were obtained directly from the articles or the appendix. For studies reporting medians with interquartile ranges, data were converted using the Cochrane-recommended online tool (math.hkbu.edu.hk/~tongt/papers/median2mean.html). Missing data (Lizano et al., 2016) were supplemented from prior meta-analyses.

We extracted baseline demographics (age, sex, BMI, smoking status, medication use), blood sampling details, comorbidities, and study characteristics (e.g., design, population, assessment tools). Categorical variables are summarized as proportions and continuous variables as mean ± SD in Supplementary Table S4. Unreported variables are listed as ‘NA’; healthy controls were assumed free of psychotropic medication. Sensitivity and meta-regression analyses were performed to examine potential sources of between-study bias.

### Statistical analysis

For all inflammatory markers, a frequentist NMA was performed if both CHR and GHR groups had at least one study contributing data and the total number of included articles was ≥3. Three articles were excluded from NMA due to insufficient data from both groups (Supplementary Table S6). NMA integrates direct and indirect evidence. For each eligible marker, a network plot was constructed (Supplementary Figure S2): nodes represent intervention groups (CHR, GHR, healthy controls HC), sized proportionally to sample sizes; edges indicate available direct comparisons. A random-effects model was used, with effect sizes estimated as standardized mean differences (SMDs) and 95% confidence intervals (CIs). SMDs of 0.2, 0.5, and 0.8 correspond to small, medium, and large effects, respectively (Leppink, O’Sullivan, & Winston, 2016). The Benjamini–Hochberg method adjusted p-values to control for multiple testing. Transitivity was assessed by comparing potential effect modifiers (mean age, sex distribution, antipsychotic use, blood draw timing) across CHR vs. HC and GHR vs. HC comparisons (Supplementary Figure S1). Network heterogeneity and inconsistency were evaluated using *I* ^2^ and *Q* statistics from a design-by-treatment interaction model (Borenstein, Hedges, Higgins, & Rothstein, 2010; Higgins, Thompson, Deeks, & Altman, 2003). Network meta-regression examined covariates including age, sex, and medication (5,000 adaptations, 10,000 iterations, thinning of 20). Education, BMI, nicotine, and baseline symptom severity were not analyzed due to inadequate data.

Pairwise meta-analyses were conducted using random-effects models with Cohen’s d as the effect size for two comparisons: (1) comparing SMD between CHR and HC when GHR lacked data, and (2) comparing concentrations between CHR-T and CHR-NT populations. Forest plots were generated for each inflammatory marker. Sensitivity analysis was performed by excluding each study individually to assess bias. Meta-regression was applied to markers with >2 studies to examine effects of age, gender, education, nicotine, and antipsychotics. Publication bias was assessed using funnel plots and Egger’s test for analyses with ≥3 studies.

Analyses were performed in R Studio (v4.2.3): pairwise meta-analyses used the meta and metafor packages, network meta-analysis used netmeta, and Bayesian meta-regression used gemtc. A Benjamini–Hochberg adjusted *p*-value <0.05 was considered statistically significant for all analyses.

## Results

From 8263 identified references, 62 articles remained after deduplication and title/abstract screening. Full‑text review led to the exclusion of 32 articles for reasons including insufficient data, inappropriate controls, poorly defined cohorts, or sample overlap, yielding 30 included studies (Figure 1).

The 30 included studies comprised 4256 participants: 2276 individuals at psychosis risk (1601 CHR, 675 GHR) and 1980 healthy controls. CHR cohorts were investigated in 17 studies and GHR cohorts in 13 studies; no study included both risk groups. Publication years ranged from 1997 to 2024, with a mean sample size of 142 (range 18–494). Mean age was 20.6 (SD 5.5) years for CHR participants (54% male), 30.4 (12.8) years for GHR participants (43% male), and 28.9 (20.4) years for controls (52% male). Geographically, studies were predominantly conducted in China (8 studies), Brazil (6), Spain (4), and the United States (3). Most participants were diagnosed using validated clinical instruments such as the Comprehensive Assessment of At‑Risk Mental States (CAARMS), the Structured Interview for Prodromal Syndromes (SIPS), or the Scale of Psychosis‑Risk Syndromes (SOPS). The characteristics and outcomes of the included studies are summarized in Table 1, with additional details provided in Supplementary Table S4.

**Table 1.** Summary of included studies with demographic details in the meta-analysis

Study	Type (CHR/GHR)	CHR type	Sample size, *N*		Age, Years (mean,SD)		Sex proportion (males,%)		BMI, kg/m^2^ (mean, SD)		Education, years(mean, SD)		Smokers(%)		Subjects taking antipsychotics(%)	Inflammatory markers
			CHR/GHR	HC	CHR/GHR	HC	CHR/GHR	HC	CHR/GHR	HC	CHR/GHR	HC	CHR/GHR	HC	CHR/GHR	
Arolt et al., 1997	GHR	/	12	38	NA	34.6 ± 12.3	NA	47	NA	NA	NA	NA	NA	NA	NA	CD4+, CD5+, CD8+
Chouinard et al., 2019	GHR	/	22	15	24.2 ± 5.6	22.5 ± 3.5	36	41	24.6 ± 3.4	23.5 ± 4.3	5.4 ± 1.6	5.4 ± 1.3	0	0	0	CRP, IL–6, TNF-α
Corsi-Zuelli et al., 2020	GHR	/	57	251	30.7 ± 10.5	31.3 ± 11.0	32	51	24.9 ± 4.9	26.2 ± 5.3	NA	NA	18	17	0	IFN-γ, IL–1β, IL–4, IL–6, IL–10, TNF-α, TGF-β
Corsi-Zuelli et al., 2022	GHR	/	25	78	29.8 ± 8.7	54.0 ± 69.2	32	69	25.8 ± 5.2	26.5 ± 5.8	NA	NA	20	10	0	IL–6
Corsi-Zuelli et al., 2024	GHR	/	66	100	31.7 ± 10.7	31.6 ± 11.4	32	54	25.3 ± 4.9	26.0 ± 5.2	NA	NA	66	NA	0	CRP, IFN-γ, IL–1β, IL–4, IL–6, IL–10, TNF-α, TGF-β
Delaney et al., 2019	CHR	APSS/BIPS/GRD	17	33	22.8 ± 3.7	25.3 ± 4.8	65	42	23.9 ± 4.0	24.8 ± 6.3	NA	NA	NA	NA	NA	CRP, IL–6
Gallart-Palau et al., 2023	CHR	APSS/BIPS/GRD	58	67	22.3 ± 5.7	23.6 ± 4.6	69	52	22.3 ± 3.6	22.5 ± 3.3	NA	NA	33	34	31	CRP, Fibrinogen, IL–6
Gaughran et al., 2002	GHR	/	51	126	33.5 ± 19.6	44.2 ± 32.5	41	51	NA	NA	NA	NA	NA	NA	NA	sIL2-RA
Kelsven et al., 2020	CHR	APSS/BIPS/GRD	11	7	18.5 ± 4.7	20.4 ± 4.9	64	71	NA	NA	NA	NA	NA	NA	0	BDNF, IFN-γ, IL–1β, IL–6, IL–8, IL–10, IL–12p70, TNF-α
Labad et al., 2015	CHR	APSS/BIPS/GRD	39	44	22.3 ± 4.6	23.2 ± 4.4	69	65	21.9 ± 3.5	22.4 ± 4.3	NA	NA	NA	NA	18	CRP, Fibrinogen
Lizano et al., 2016	GHR	/	35	39	16.5 ± 0.6	25.1 ± 1.0	43	64	NA	NA ± NA	NA	NA	NA	NA	0	BDNF, IFN-γ, IL–1β, IL–6, IL–8, IL–10, IL–12, TNF-α, TNF-β
Mondelli et al., 2023	CHR	APSS/BIPS/GRD	269	56	26.6 ± 0.4	22.9 ± 0.5	55	54	24.4 ± 0.5	22.4 ± 0.5	14.3 ± 0.3	16.1 ± 0.4	54	25	0	IFN-γ, IL–1β, IL–2, IL–4, IL–5, IL–6, IL–8, IL–10, IL12p70, IL–13, IL–15, TNF-α, TNF-β
Moreno et al., 2023	CHR	APSS/BIPS/GRD	34	42	23.1 ± 5.0	23.0 ± 4.0	68	64	22.0 ± 3.7	22.7 ± 3.6	10.7 ± 2.3	13.0 ± 2.5	44	29	29	CRP, Fibrinogen, IL–6
Noyan et al., 2021	GHR	/	139	210	31.3 ± 10.0	36.7 ± 11.5	44	31	25.0 ± 3.5	25.3 ± 4.3	11.5 ± 4.2	11.2 ± 4.5	45	44	NA	IL–1β
Ntouros et al., 2018	CHR	APSS/BIPS/GRD	12	23	24.5 ± 3.1	27.0 ± 2.9	100	100	NA	NA	NA	NA	NA	NA	NA	IFN-γ, IL–1β, IL–2, IL–4, IL–5, IL–8, IL–10, IL–12, IL–12p70, TNF-α, TNF-β,
Nunes et al., 2006	GHR	/	41	48	NA	NA	NA	NA	NA	NA	NA	NA	NA	NA	NA	IL–6
Ouyang et al., 2022	CHR	APSS/BIPS/GRD	49	30	18.3 ± 3.5	19.0 ± 2.9	49	70	NA	NA	11.0 ± 2.6	12.2 ± 2.3	NA	NA	0	IFN-γ, IL–1β, IL–2, IL–4, IL–6, IL–17, TNF-α, TNF-β,
Perkins et al., 2015	CHR	APSS	72	35	19.4 ± 4.2	20.0 ± 4.5	66	66	NA	NA	NA	NA	30	9	20	BDNF, CRP, Fibrinogen, IL–1β, IL–4, IL–5, IL–6, IL–8, IL–10, IL–12, IL–13, IL–15, TGF-β, TNF-α
Piotrowski et al., 2019	GHR	/	37	42	36.9 ± 11.0	27.8 ± 8.4	32	38	24.3 ± 4.1	24.0 ± 3.5	15.7 ± 3.6	15.7 ± 2.5	19	10	0	CRP, Fibrinogen
Rebouças et al., 2018	GHR	/	36	47	40.5 ± 19.0	41.0 ± 26.0	42	45	25.4 ± 3.6	26.0 ± 4.1	NA	NA	14	17	NA	IL–6, TNF-α
Stojanovic et al., 2014	CHR	APSS/BIPS/GRD	17	25	21.4 ± 2.4	27.3 ± 4.2	71	48	22.1 ± 3.2	21.6 ± 2.5	NA	NA	41	24	47	CRP, Fibrinogen, IL–6
Wang et al., 2020	GHR	/	99	130	21.7 ± 5.1	21.0 ± 4.6	60	46	23.0 ± 4.7	21.7 ± 4.0	12.4	12.4	NA	NA	NA	IL–1β, IL–6, TNF-α
Wang et al., 2024	CHR	APSS/BIPS/GRD	51	51	19.5 ± 5.1	20.8 ± 2.9	55	57	NA	NA	11.0 ± 2.4	13.8 ± 2.4	NA	NA	NA	IL–1β, IL–2, IL–4, IL–6, IL–17
Yüksel et al., 2020	GHR	/	55	58	39.3 ± 11.5	37.6 ± 8.8	56	64	25.9 ± 4.0	25.6 ± 3.6	NA	NA	42	48	NA	Gal–1, Gal–3
Zeni-Graiff et al., 2016	CHR	APSS/BIPS	12	16	17.7 ± 3.4	18.7 ± 4.1	75	69	21.3 ± 4.1	23.5 ± 4.0	11.0 ± 3.4	13.0 ± 4.1	NA	NA	42	IFN-γ, IL–6, IL–17
Zhang et al., 2022	CHR	APSS/BIPS/GRD	84	65	18.6 ± 5.2	18.8 ± 5.1	58	48	NA	NA	10.4 ± 2.9	11.8 ± 2.5	NA	NA	0	IL–1β, L–6
Zhang et al., 2024	CHR	APSS/BIPS/GRD	385	95	18.9 ± 5.4	18.7 ± 4.9	48	54	NA	NA	10.9 ± 3.09	11.7 ± 2.3	NA	NA	NA	IL–1β, IL–2, IL–6, IL–8, TNF-α
Zhang, Wei, et al., 2023	CHR	APSS/BIPS/GRD	37	49	17.6 ± 3.7	18.3 ± 4.5	52	55	NA	NA	10.2 ± 2.8	11.5 ± 2.4	NA	NA	0	IL–2, IL–6
Zhang, Xiao, et al., 2023	CHR	APSS/BIPS/GRD	60	60	19.7 ± 6.1	20.8 ± 2.6	42	45	NA	NA	11.6 ± 3.1	12.4 ± 2.5	NA	NA	NA	IL–1β, IL–2, IL–6, IL–8, IL–10, TNF-α
Zhang et al., 2023	CHR	APSS/BIPS/GRD	394	100	18.9 ± 5.4	18.8 ± 5.2	49	52	NA	NA	11.0 ± 3.1	11.0 ± 3.1	NA	NA	NA	IL–1β, IL–2, IL–6, IL–8, IL–10, TNF-α

Abbreviations: BMI = body mass index; CHR = clinical high risk; GHR = genetic high risk; HC = healthy controls; NA = not available; BDNF = brain derived neurotrophic factor; CRP=C-reactive protein; IL = interleukin; IFN = interferon; TGF = transforming growth factor; TNF = tumor necrosis factor; APSS = attenuated positive symptom syndrome; BIPS = brief intermittent psychotic symptom syndrome; GRD = genetic risk and deterioration.

**Figure 2.**
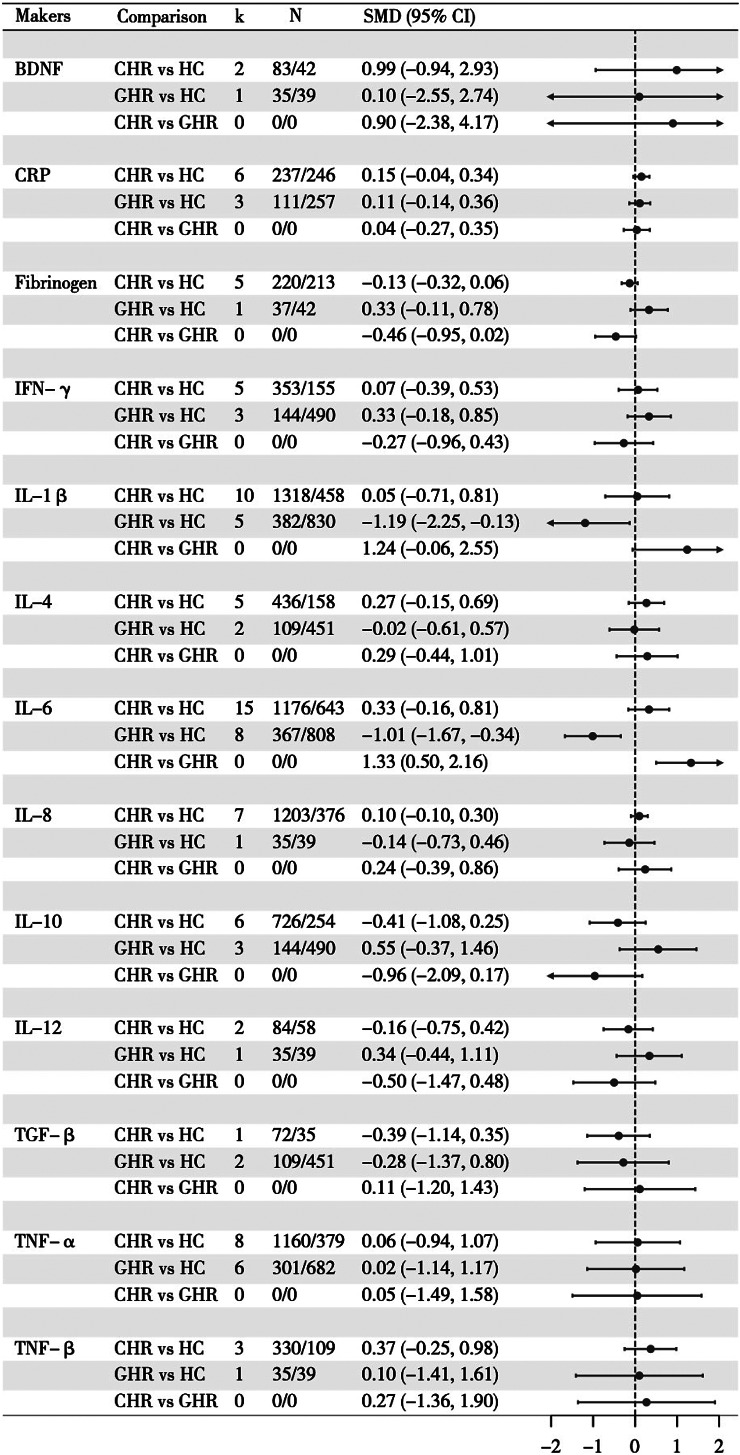
Network meta-analysis results of inflammatory makers in genetic and clinical high-risk groups of psychosis, compared with healthy controls. *K* denotes the number of studies with data for direct network comparison. *Number of participants in the respective first and second groups of each comparison (from studies with direct data). Abbreviations: SMD = standardized mean difference; CI = confidence interval; BDNF = brain derived neurotrophic factor; CHR = clinical high risk; GHR = genetic high risk; CRP = C-reactive protein; IFN = interferon; IL = interleukin; TGF = transforming growth factor; TNF = tumor necrosis factor. *Note*: The network meta-analysis was informed solely by comparisons of CHR vs. HC and GHR vs. HC. The estimate for **CHR vs. GHR** is derived from indirect comparison, as no direct head-to-head studies were available.

Comparisons of 13 inflammatory proteins were conducted individually in the network meta-analysis (Supplementary Table S9). The markers reported in each study were presented in Supplementary Table S7. IL-6 (23 studies), IL‑1β (15), and TNF‑α (14) were the most frequently studied. All network plots (Supplementary Figure S2) showed a star‑shaped structure with no direct link between CHR and GHR groups, meaning their comparison relied entirely on indirect evidence via HC. NMA (Figure 2) and adjusted *p*-value (Table 2) demonstrated that IL‑6 concentrations were significantly lower in the GHR group than in both the CHR group (SMD − 1.33, 95% CI − 2.16 to − 0.50) and HC (SMD − 1.01, −1.67 to − 0.34). IL‑1β was also lower in GHR versus HC (SMD − 1.19, −2.25 to − 0.13), though this did not survive multiple‑testing correction. No other markers showed significant differences.

**Table 2.** *p* Value and adjusted *p* value for each comparison in the network meta-analysis

Comparison		*p* Value	Adjusted *p*-value
BDNF	CHR vs HC	0.3148	0.8880
	GHR vs HC	0.9429	0.9429
	CHR vs GHR	0.5920	0.8880
CRP	CHR vs HC	0.1187	0.3561
	GHR vs HC	0.3804	0.5706
	CHR vs GHR	0.8037	0.8037
Fibrinogen	CHR vs HC	0.1897	0.1897
	GHR vs HC	0.1420	0.1852
	CHR vs GHR	0.0617	0.1852
IFN-γ	CHR vs HC	0.7706	0.7706
	GHR vs HC	0.2058	0.6175
	CHR vs GHR	0.4541	0.6811
IL1-β	CHR vs HC	0.8933	0.8933
	GHR vs HC	**0.0278**	**0.0927**
	CHR vs GHR	0.0618	0.0927
IL-4	CHR vs HC	0.2022	0.6067
	GHR vs HC	0.9578	0.9578
	CHR vs GHR	0.4370	0.6555
IL-6	CHR vs HC	0.1906	0.1906
	GHR vs HC	**0.0032**	**0.0048**
	CHR vs GHR	**0.0016**	**0.0048**
IL-8	CHR vs HC	0.3275	0.6513
	GHR vs HC	0.6513	0.6513
	CHR vs GHR	0.4559	0.6513
IL-10	CHR vs HC	0.2262	0.2411
	GHR vs HC	0.2411	0.2411
	CHR vs GHR	0.0969	0.2411
IL-12	CHR vs HC	0.5883	0.5883
	GHR vs HC	0.3973	0.5883
	CHR vs GHR	0.3165	0.5883
TGF-β	CHR vs HC	0.6096	0.8689
	GHR vs HC	0.2991	0.8689
	CHR vs GHR	0.8689	0.8689
TNF-α	CHR vs HC	0.9043	0.9796
	GHR vs HC	0.9796	0.9796
	CHR vs GHR	0.9526	0.9796
TNF-β	CHR vs HC	0.2458	0.7373
	GHR vs HC	0.9006	0.9006
	CHR vs GHR	0.7460	0.9006

*Note*: Bold shows significance.

Abbreviations: CHR = clinical high risk; GHR = genetic high risk; HC = healthy controls; BDNF = brain derived neurotrophic factor; CRP=C-reactive protein; IL = interleukin; IFN = interferon; TGF = transforming growth factor; TNF = tumor necrosis factor.

Pairwise meta-analyses were conducted for 7 inflammatory markers between the CHR group and healthy controls across 11 studies (Supplementary Table S9), showing no significant differences in concentrations (Supplementary Figure S4). Additional pairwise meta-analyses for 13 markers between CHR individuals who did or did not transition to psychosis (6 studies) included 955 participants, of whom 288 (30.2%) transitioned within 12–24 months (Supplementary Table S10). Only IL-13 was significantly elevated in those who transitioned compared to non-transitioners (SMD 0.14, 0.04 to 0.23).

A quality assessment of the included studies (Supplementary Table S5) showed that 25 were rated high quality (meeting 6–7 criteria), 5 were moderate quality (3–5 criteria), and none were low quality. The lowest-rated domain related to confounding factor reporting, as 5 studies provided insufficient details. No clear transitivity violations were observed (Supplementary Figure S1), though limited study numbers per comparison may have reduced the ability to detect intransitivity. Egger’s test indicated potential small-study effects for IL-2 in CHR vs. HC pairwise analysis (Supplementary Table S11), but no significant publication bias was found overall in the network meta-analysis. Substantial heterogeneity (*I* ^2^ > 80%) was noted for 7 markers in the network meta-analysis (Supplementary Table S12), but not in pairwise analyses. Due to the absence of closed loops (Supplementary Figure S2), consistency assessment was not performed. Meta-regression – restricted to demographic variables due to data limitations – identified gender as a significant moderator for IL-4 and IL-17. A higher proportion of males was associated with greater IL-4 increases in CHR vs. HC (*β* = 1.17, 0.19–2.05) and greater IL-17 decreases in CHR compared to controls (*β* = −0.14, p = 0.03). Sensitivity analyses (leave-one-out) generally showed robust results, except for IL-10, where omitting one study (Zhang et al., 2023) revealed significantly higher levels in CHR individuals who transitioned to psychosis.

## Discussion

Our meta-analysis identified inflammatory cytokine alterations in individuals presenting with CHR and GHR. However, it must be emphasized that the insufficient statistical power significantly limits the interpretability of these findings. Specifically, we observed increased levels of IL-6 in the CHR group, compared to the GHR group. Additionally, individuals with GHR exhibited decreased IL-6 and IL-1β levels compared to healthy controls. Conversely, CHR patients demonstrated predominantly non-significant differences in inflammatory cytokine levels. Furthermore, our subgroup analysis uncovered a statistically significant elevation in IL-13 levels among individuals diagnosed with CHR-T. These observed patterns of abnormal cytokine levels potentially signify the presence of immune imbalances during the pre-psychotic phases. Notably, these imbalances vary across genetic and clinical risk groups for psychosis, suggesting that genetic and environmental factors may involve distinct immune pathological mechanisms. Understanding these differences could provide insights into the diverse pathways leading to the development of psychosis, though definitive conclusions require verification through larger-scale studies.

### Inflammatory cytokine alterations in GHR and CHR patients

Our network meta-analysis reveals distinct inflammatory patterns in CHR and GHR individuals for psychosis. CHR individuals display significantly higher IL-6 levels compared to GHR, consistent with Misiak B et al.’s meta-analysis (Misiak et al., 2021). IL-6, a core factor in central nervous system immune activation, influences key pathological processes such as neurotransmitter metabolism, neurodevelopment, and synaptic plasticity (Gruol, 2015; Zhou, Tian, & Han, 2021). GHR individuals primarily reflect genetic susceptibility, whereas CHR individuals, in addition to potential genetic risk, already exhibit subclinical manifestations and functional impairment (Fusar-Poli et al., 2017; Niznikiewicz, 2019). The specific elevation of IL-6 in CHR subjects suggests that immune activation may mark a tipping point in the transition from genetic risk to clinical phenotype. This aligns with the ‘environmental trigger’ hypothesis: environmental factors (such as stress or infections) activate neuroimmune responses against a backdrop of genetic susceptibility, thereby driving disease progression (Howes & Onwordi, 2023). These observations in GHR and CHR cohorts corroborate earlier findings within the high-risk psychosis research domain. Accumulating studies of peripheral blood concentrations of cytokines (e.g., IL-6) have found that a low-grade and pro-inflammatory immune mechanism is activated in the presence of psychotic symptoms (Goldsmith, Rapaport, & Miller, 2016; Miller et al., 2011). In summary, the fluctuating levels of IL-6 during the CHR stage underscore the potential link between clinical symptoms and immune activation.

Furthermore, it should be noted that changes in the specific cytokine IL-6 must be understood within the broader context of immune regulatory networks. Previous studies have shown that fluctuations in IL-6 often occur in dynamic equilibrium with other pro-inflammatory factors (such as TNF-α and IL-1β) and anti-inflammatory mediators (Roomruangwong et al., 2020). However, limited data may prevent us from reliably detecting these coordinated inflammatory changes. Consequently, the elevated IL-6 levels observed in this study may only represent the ‘tip of the iceberg’ in terms of complex immune dysregulation patterns.

Overall, although there is currently a lack of studies directly comparing CHR and GHR populations, this research, by integrating an indirect evidence network, has quantitatively analyzed the differences in inflammatory levels between the CHR and GHR stages, confirming that IL-6 levels are significantly higher in CHR individuals than in GHR individuals. However, the limitations of the existing data suggest that future studies should prioritize expanded sample sizes and multi-center collaborations to enhance statistical power and better elucidate the mechanisms of immune dysregulation across different stages of psychiatric risk.

### Inflammatory cytokine alterations in GHR patients

Compared with HC, IL-6 levels in GHR group were significantly reduced both before and after multivariable adjustment, whereas IL-1β was lower than in HC only in the unadjusted model, with this difference disappearing after correction. Prior studies comparing plasma cytokine levels between GHR and HC are limited. Most found no alterations (Allswede, Yolken, Buka, & Cannon, 2020; Wang et al., 2020), but some reported reduced immune activation in GHR, like decreased IL-1β and increased IL-10 (Corsi-Zuelli et al., 2020; Lizano et al., 2016). Our results support the latter pattern, suggesting that an immunosuppressive state may be a potential endophenotype for psychosis.

Although this study found decreased IL-6 levels in individuals at GHR, the current analysis relies on relatively limited data, which may obscure coordinated changes of other cytokines within the immunoregulatory network. Therefore, future studies with larger sample sizes are still needed to further explore the potential immunopathological mechanisms during the development of psychosis in genetic high-risk populations.

### Inflammatory cytokine alterations in CHR patients

Both pairwise and NMA analyses did not reveal significant differences in immune factors between CHR and control groups. This negative result can largely be attributed to the limited number of studies. In addition, the clinical heterogeneity within the CHR population (including different subtypes such as APSS, BIPS, and GRD) and potential confounding factors (such as medication history, and comorbid status) may also have weakened the ability to detect true differences. Therefore, future studies with larger samples and more uniform designs are needed to further clarify these effects.

However, it is noteworthy that most pro-inflammatory factors we assessed exhibited an elevated trend in CHR patients. Previous studies have observed alterations in inflammatory cytokines (IL-1β, IL-4, IL-6, IL-17) in individuals at clinical high risk for psychosis (Karanikas et al., 2017; Lizano et al., 2016; Metcalf et al., 2017; Smesny et al., 2017; Zeni-Graiff et al., 2016). Two meta-analyses also reported increased IL-6 levels in CHR subjects, indicating immune system activation (Misiak et al., 2021; Park & Miller, 2020). Thus, our findings support the hypothesis of inflammatory abnormalities in clinical high-risk psychosis, consistent with the theory of a pre-clinically activated immune state, suggesting subclinical inflammation before psychosis onset (Trovão et al., 2019).

### Inflammatory cytokine alterations in CHR-NT and CHR-T groups

Our subgroup analysis revealed a conversion rate of 30.2% in the CHR group, closely aligning with the 29% reported in a meta-analysis, confirming the reliability of our sample (Fusar-Poli et al., 2012). Within this context, we observed significantly elevated serum IL-13 levels among CHR individuals who later developed psychosis, compared to those who did not. As a member of the Th2 cytokine family, the upregulation of IL-13 suggests activation of the Th2 pathway during the transformation process. This pathway may suppress pro-inflammatory responses by antagonizing inflammatory factors such as IL-6 and TNF-α, thereby protecting neurons (Mamuladze & Kipnis, 2023). Consistent with prior studies, we did not detect elevations in pro-inflammatory cytokines (Khoury & Nasrallah, 2018; Mondelli et al., 2023; Zhang et al., 2023). Collectively, these findings indicate that specific activation of the Th2 pathway may represent a critical juncture in the transition from CHR to psychosis.

However, it must be noted that the current analysis is based on a very limited number of studies (*n* = 6) and demonstrates a small effect size, which significantly constrains the reliability of this conclusion. Therefore, larger-scale studies with greater statistical power are needed to further investigate the role of the Th2 pathway during the conversion process in the prodromal stage of psychosis.

### Strengths and limitations

A key strength of this study lies in the application of network meta-analysis. By integrating multi-source trial data and indirect evidence, we constructed a robust three-arm comparison network encompassing genetic high-risk, clinical high-risk, and control groups. Notably, no previous studies have directly compared inflammatory factor levels between individuals at CHR and GHR for psychosis. This study is the first to employ NMA to systematically quantify and compare differences in immune-inflammatory biomarker levels across these two critical risk stages, thereby addressing an important evidence gap in the field.

Compared to conventional meta-analysis, which is limited to pairwise direct comparisons, NMA offers several distinct advantages. In the absence of head-to-head studies comparing CHR and GHR, NMA enables valid indirect comparisons by leveraging shared control groups. Furthermore, by incorporating all relevant comparisons into a single analytical framework, NMA avoids the multiple testing issues inherent in traditional piecewise analyses and ensures consistency of results.

However, our study also has several limitations. First, only 30 studies were included in this meta-analysis, which may have underpowered the overall effect of immunoinflammatory system on genetic and clinical high-risk psychosis. The limitation of including only English original articles may introduce bias; however, the influence of this element is often described as small (Jüni et al., 2002). Additionally, this network meta-analysis was limited by a sparse network structure. As shown in Supplementary Figure S2, the absence of direct comparative data on inflammatory markers between the GHR and CHR groups, along with the reliance solely on indirect evidence, restricted the ability to reliably differentiate between the two groups. Furthermore, uncontrolled factors such as smoking, drinking, gender, BMI, medication usage, and variations in cytokine measurements could potentially bias our results, despite sensitivity analyses revealing no significant relationships. Given the impact of other environmental factors, such as stress, infections, malnutrition, and toxins, on immune responses and immune expression, we were unable to address their differential effects on immune factor outcomes of GHR and CHR. Moreover, the inability to conduct analyses for variables with fewer than three included studies and the lack of examination of specific subtypes of individuals at clinical risk of psychosis (APSS, BIPS, and GRD) represent further limitations. We were unable to address their differential effects on the levels of immune-inflammatory markers. These limitations highlight the need for future research to expand sample sizes, refine network structures, and enhance control and analysis of confounding factors and subgroup characteristics.

## Conclusion

In summary, given the fragmented evidence in existing literature and the lack of head-to-head comparative data, this study marks the first systematic evaluation of peripheral blood inflammatory markers in genetic and clinical high-risk patients with psychosis, utilizing both NMA and pairwise analysis. The findings reveal distinct patterns of immune dysregulation in CHR and GHR individuals. Specifically, CHR individuals exhibit higher IL-6 levels compared to GHR individuals, suggesting that immune activation may mark a critical juncture in the transition from genetic susceptibility to clinical phenotype. Conversely, GHR individuals present an immune hypofunction state with reduced IL-6. Although most inflammatory factors did not show statistically significant differences between the CHR group and healthy controls, the observed upward trends, coupled with the specific elevation of IL-13 levels in converters (CHR-T) identified in subgroup analysis, collectively support the presence of immune abnormalities in the clinical high-risk stage and suggest that Th2 pathway activation may play a particular role in the disease conversion process.

It should be noted that the conclusions of this study are limited by the small number of included studies, population heterogeneity, and potential confounding factors, and therefore should be interpreted with caution. Future research should involve larger-scale multicenter studies to further explore the immune mechanisms in the high-risk stages of psychosis, thereby providing a theoretical foundation for the development of potential immune intervention strategies.

## Supporting information

10.1017/S0033291726103559.sm001 Huang et al. supplementary material Huang et al. supplementary material

## Data Availability

All the data that support the findings of the present study are available from the corresponding author through request.

## Supplementary material

The supplementary material for this article can be found at http://doi.org/10.1017/S0033291726103559.
