# Supplementary material for: Inflammatory cytokine alterations in genetic and clinical high risk groups of psychosis: a systematic review and network meta-analysis
Source: Psychol Med. 2026 Apr 10;56:e99. doi: 10.1017/S0033291726103559 (PMC13079210; doi:10.1017/S0033291726103559)
Supplement: Huang et al. supplementary material [file S0033291726103559sup001.pdf]

Online Supplementary Materials

Inflammatory Cytokine Alterations in Genetic and Clinical High Risk Groups of Psychosis: A systematic review and network meta-analysis

Supplementary Content

Table S1. Search keywords 2
Table S2. List of accepted cytokines and associated biomarkers for data extraction. 2
Table S3. Quality Appraisal Criteria - adapted from Joanna Briggs Institute. 3
Table S4. Table of included studies with additional characteristics. 5
Table S5. Overview of quality appraisal of included studies (criteria as per those in table S3, study references as per table S4) 15
Table S6. A list of articles not analyzed. 17
Table S7. Table of inflammatory markers examined in each included study. 17
Table S8. List of the number of articles on the inflammatory markers. 19
Table S9. Table of included studies in pairwise meta-analysis for CHR and HC groups. 21
Table S10. Table of included studies in pairwise meta-analysis for CHR-NT and CHR-T groups. 22
Table S11. Egger's regression tests for publication bias. 23
Table S12. Network meta-analysis inconsistency results. 24
Table S13. Bayesian Network Meta-Regression on age, gender, BMI, nicotine and medicine 26
Table S14. Meta regression in pairwise meta-analysis (CHR vs HC). 28
Table S15. Meta regression in pairwise meta-analysis (CHR-T vs CHR-NT). 28
Figure S1. Transitivity assessments of NMA analysis. 30
Figure S2. Network Geometry Plots. 44
Figure S3. Funnel plots of NMA analysis. 46
Figure S4. Pairwise meta-analysis results of comparison of makers between CHR and HC groups. 50
Figure S5. Results of sensitivity analysis tests for pairwise meta-analyses between CHR and HC groups. 51
Figure S6. Funnel plots of pairwise analysis of inflammatory factors between CHR and HC groups. 53
Figure S7. Pairwise meta-analysis results of comparison of makers between CHR-T and CHR-NT groups. 55
Figure S8. Results of sensitivity analysis tests for pairwise meta-analyses between CHR-T and CHR-NT groups. 59
Figure S9. Funnel plots of pairwise analysis of inflammatory factors between CHR-T and CHR-NT groups. 61
References for included studies (numbered as per table S4 & S5): 66
PRISMA 2020 checklist 69

Table S1. Search keywords

1) High risk for psychosis	("prodrom" OR "ultra-high risk" OR "clinical high risk" OR "high clinical risk" OR "high risk" OR "risk" OR "at risk mental state" OR "subthreshold" OR "sub-threshold" OR "subclinical" OR "sub-clinical" OR "attenuate" OR "basic symptoms" OR "genetic risk" OR "familial risk" OR "parental risk" OR "relatives" OR "twins" OR "offspring" OR "siblings")<br>AND<br>("psychosis OR "psychotic" OR "prepsychotic" OR "pre-psychotic" OR "prepsychosis" OR "pre-psychosis" OR "schizophrenia")
AND	
2) Cytokines and associated inflammation markers	("Cytokines" OR "C-Reactive Protein" OR cytokineOR interleukin OR "tnf-α" OR "tumour necrosis factor" OR "tumor necrosis factor" OR "tnf-α" OR "transforming growth factor-β*" OR "tgf-β*" OR crp OR "c-reactive protein" OR chemokine* OR monokine* OR "inflammatory markers" OR "markers of inflammation" OR "inflammation markers" OR "interferon OR "interferon-γ OR "IFN-γ" OR "IL-1α OR "IL-1β" OR "IL-1RA" OR "IL-2" OR "IL-3" OR "IL-4" OR "IL-5" OR "IL-6" OR "IL-8" OR "IL-10" OR "IL-17" OR "IL-18" OR "IL-23" OR "sIL-2R" OR "sIL-6R"<br>OR sTNF-R1 OR sTNF-R2)

Table S2. List of accepted cytokines and associated biomarkers for data extraction.

Category	Cytokines and associated biomarkers
Interleukins (IL-)	IL-1α, IL-1β, IL-1RA, IL-2, IL-3, IL-4, IL-5, IL-6, IL-8, IL-10, IL-12, IL-17, IL-18, IL-23, IL-27, IL-33, sIL-2R,sIL-6R
Tumor necrosis factors (TNF-)	TNF-α, TNF-β, sTNF-R1, sTNF-R2
Other	interferon-γ(IFN-γ), C-reactive protein (CRP), transforming growth factor (TGF-β), brain derived neurotrophic factor (BDNF), Fibrinogen

Table S3. Quality Appraisal Criteria - adapted from Joanna Briggs Institute.

#	Type	Criteria
C1	Group Comparability	Were the groups comparable other than the presence of disease in cases or the absence of disease in controls?
C2	Matching	Were cases and controls matched appropriately?
C3	Criteria Consistency	Were the same criteria used for identification of cases and controls?
C4	Confounders Identified	Were confounding factors identified?
C5	Strategies for Confounding	Were strategies to deal with confounding factors stated?

C6	Outcome Assessment Validity	Were outcomes assessed in a standard, valid and reliable way for cases and controls?
C7	Statistical Methodology	Was appropriate statistical analysis used?

Use of criteria

Each included article was reviewed against all seven criteria. For each criterion, articles were scored as either “Yes” if they met the criteria, “No” if they contravened the criteria, or “Unclear” if they did not provide sufficient evidence. The number of criteria answered with “Yes” were scored out of seven for each article. The following scoring system was used:

0 to 2 – Low quality
3 to 5 – Moderate quality
6 or 7 – High quality
The total score was divided by 7 to calculate the “quality index” referenced in table S5.

Table S4. Table of included studies with additional characteristics.

Study	Journal	Country	Type (CHR/GHR)	Diagnosis Criteria / Definition of high psychosis risk	blood draw timing	anti-inflammatory drugs	comorbidities
Arolt et al., 1997	Psychiatry Research	Germany	GHR	healthy first-degree relatives of schizophrenia	NA	0	0
Chouinard et al., 2019	Mol Psychiatry	USA	GHR	unaffected siblings of patients with	Fasting state	0	0

				first episode psychosis			
Corsi-Zuelli et al., 2019	Psychological Medicine	Brazil	GHR	unaffected siblings of patients with first episode psychosis	NA	0	0
Corsi-Zuelli et al., 2022	Translational Psychiatry	Brazil	GHR	unaffected siblings of patients with first episode psychosis	NA	0	0

Corsi-Zuelli et al., 2024	Psychological Medicine	Brazil	GHR	unaffected siblings of psychosis patients	NA	0	0
Delaney et al., 2019	Schizophrenia research	USA	CHR	from the prodromal clinic, the Center for Prevention and Evaluation (COPE)	NA	NA	NA

Gallart-Palau et al., 2023	Journal of Proteome Research	Spain	CHR	CAARMS	Fasting state	NA	0
Gaughran et al., 2002	Schizophrenia research	UK	GHR	siblings of schizophrenia patients	Noon to 6 p.m	NA	0
Kelsven et al., 2020	Schizophrenia research	Mexico	CHR	SIPS	Fasting state	NA	0
Labad et al., 2015	Journal of Psychiatric Research	Spain	CHR	CAARMS	Fasting state	NA	NA
Lizano et al., 2016	Schizophrenia Research	USA	GHR	first- or second-degree	Fasting state	NA	0

				relative diagnosed with schizophrenia or schizoaffective disorder			
Mondelli et al., 2023	Brain, Behavior, & Immunity	NA	CHR	CAARMS	NA	NA	NA
Moreno et al., 2023	Irene Moreno	Spain	CHR	CAARMS	Fasting state	0	0

Noyan et al., 2021	Schizophrenia Research	European	GHR	unaffected siblings	Fasting state	NA	NA
Ntouros et al., 2018	Cognitive Neuropsychiatry	Greece	CHR	CAARMS	Fasting state	0	0
Nunes et al., 2006	Schizophrenia Research	Brazil	GHR	biological relatives of patients with schizophrenia or schizoaffective disorder	NA	NA	NA

Ouyang et al., 2022	Frontiers in Psychiatry	China	CHR	SIPS	Fasting state	0	0
Perkins et al., 2015	Schizophrenia Bulletin	NA	CHR	SIPS	NA	NA	NA
Piotrowski et al., 2019	Schizophrenia Research	Poland	GHR	healthy offspring of schizophrenia patients	Fasting state	NA	NA
Rebouças et al., 2018	Neuroimmuno modulation	Brazil	GHR	unaffected siblings of patients with schizophrenia	NA	0	0

Stojanovic et al., 2014	Psychoneuroendocrinology	Spain	CHR	CAARMS	Fasting state	NA	NA
Wang et al., 2020	Schizophrenia research	China	GHR	offspring of patients with schizophrenia	Fasting state	NA	NA
Wang et al., 2024	Cerebral Cortex	China	CHR	SIPS	10 a.m. to 3 p.m	NA	0
Yüksel et al., 2020	Psychiatric Quarterly	Turkey	GHR	unaffected siblings of patients with schizophrenia	Fasting state	NA	NA
Zeni-Graiff et al., 2016	Schizophrenia Research	Brazil	CHR	CAARMS	Fasting state	0	0

Zhang et al., 2022	Psychiatry Research	China	CHR	SIPS	Fasting state	0	NA
Zhang et al., 2024	Progress In Neuro-psychopharmacology & Biological Psychiatry	China	CHR	SIPS	Fasting state	0	0
Zhang, Wei, et al., 2023	Psychiatry and Clinical Neurosciences	China	CHR	SIPS	NA	NA	NA

Zhang, Xiao, et al., 2023	The Journal of Clinical Psychiatry	China	CHR	SIPS	NA	NA	NA
Zhang, Zeng, et al., 2023	Neuropsychobiology	China	CHR	SIPS	NA	NA	NA

Abbreviations: CHR=clinical high risk; GHR=genetic high risk; HC= healthy controls; NA=not available; CAARMS= Comprehensive Assessment of at Risk mental States; SOPS=Scale of Psychosis Risk Syndromes; SIPS= Structured Interview for Prodromal Syndromes;

Table S5. Overview of quality appraisal of included studies (criteria as per those in table S3, study references as per table S4)

Study	Group Comparability	Matching	Criteria Consistency	Confounders Identified	Strategies for Confounding	Outcome Assessment Validity	Statistical Methodology	Quality Index
Arolt et al., 1997	Yes	Yes	Unclear	Unclear	Unclear	Yes	Yes	0.57
Chouinard et al., 2019	Yes	Yes	Yes	Yes	Yes	Yes	Yes	1
Corsi-Zuelli et al., 2019	Yes	Yes	Yes	Yes	Yes	Yes	Yes	1
Corsi-Zuelli et al., 2022	Yes	Yes	Yes	Yes	Yes	Yes	Yes	1
Corsi-Zuelli et al., 2024	Yes	Yes	Yes	Yes	Yes	Yes	Yes	1
Delaney et al., 2019	Yes	Yes	Unclear	Unclear	Unclear	Yes	Yes	0.67
Gallart-Palau et al., 2023	Unclear	Yes	Yes	Yes	Yes	Yes	Yes	0.86
Gaughran et al., 2002	Unclear	Unclear	Yes	Yes	Yes	Yes	Yes	0.71
Kelsven et al., 2020	Yes	Yes	Yes	Yes	Yes	Yes	Yes	1
Labad et al., 2015	Yes	Yes	Yes	Yes	Yes	Yes	Yes	1
Lizano et al., 2016	Yes	Yes	Yes	Yes	Yes	Yes	Yes	1
Mondelli et al., 2023	Unclear	Yes	Yes	Yes	Yes	Yes	Yes	0.86
Moreno et al., 2023	Yes	Yes	Yes	Yes	Yes	Yes	Yes	1
Noyan et al., 2021	Yes	Unclear	Unclear	Yes	Yes	Yes	Yes	0.71
Ntouros et al., 2018	Yes	Yes	Yes	Yes	Yes	Yes	Yes	1
Nunes et al., 2006	Yes	Unclear	Unclear	Unclear	Unclear	Yes	Yes	0.43
Ouyang et al., 2022	Yes	Yes	Yes	Yes	Yes	Yes	Yes	1
Perkins et al., 2015	Yes	Yes	Yes	Yes	Yes	Yes	Yes	0.86
Piotrowski et al., 2019	Yes	Yes	Yes	Yes	Unclear	Yes	Yes	1

Rebouças et al., 2018	Yes	Yes	Yes	Yes	Yes	Yes	Yes	1
Stojanovic et al., 2014	Yes	Yes	Yes	Yes	Yes	Yes	Yes	1
Wang et al., 2020	Yes	Yes	Yes	Yes	Yes	Yes	Yes	0.86
Wang et al., 2024	Yes	Yes	Yes	Yes	Yes	Yes	Yes	1
Yüksel et al., 2020	Yes	Yes	Yes	Yes	Unclear	Yes	Yes	0.86
Zeni-Graiff et al., 2016	Unclear	Yes	Yes	Yes	Yes	Yes	Yes	1
Zhang et al., 2022	Yes	Yes	Yes	Yes	Yes	Yes	Yes	1
Zhang et al., 2024	Yes	Yes	Yes	Yes	Yes	Yes	Yes	1
Zhang, Wei, et al., 2023	Yes	Yes	Yes	Yes	Yes	Yes	Yes	1
Zhang, Xiao, et al., 2023	Yes	Yes	Yes	Yes	Yes	Yes	Yes	1
Zhang, Zeng, et al., 2023	Yes	Yes	Yes	Yes	Yes	Yes	Yes	1

Table S6. A list of articles not analyzed.

#	articles	Primary Reason for Exclusion
1	Arolt et al., 1997	Insufficient data - Only GHR data were available for the inflammatory factors measured in the article
2	Gaughran et al., 2002	Insufficient data - Only GHR data were available for the inflammatory factors measured in the article
3	Yüksel et al., 2020	Insufficient data - Only GHR data were available for the inflammatory factors measured in the article

Table S7. Table of inflammatory markers examined in each included study.

Study	Comparison	Inflammatory markers
Arolt et al., 1997	GHR vs HC	CD4+, CD5+, CD8+
Chouinard et al., 2019	GHR vs HC	CRP, IL-6, TNF-α
Corsi-Zuelli et al., 2019	GHR vs HC	IFN-γ, IL-1β, IL-4, IL-6, IL-10, TNF-α, TGF-β
Corsi-Zuelli et al., 2022	GHR vs HC	IL-6
Corsi-Zuelli et al., 2024	GHR vs HC	CRP, IFN-γ, IL-1β, IL-4, IL-6, IL-10, TNF-α, TGF-β
Delaney et al., 2019	CHR vs HC	CRP, IL-6
Gallart-Palau et al., 2023	CHR vs HC	CRP, Fibrinogen, IL-6
Gaughran et al., 2002	GHR vs HC	sIL2-RA
Kelsven et al., 2020	CHR vs HC	BDNF, IFN-γ, IL-1β, IL-6, IL-8, IL-10, IL-12p70, TNF-α
Labad et al., 2015	CHR vs HC	CRP, Fibrinogen

Lizano et al., 2016	GHR vs HC	BDNF, IFN-γ, IL-1β, IL-6, IL-8, IL-10, IL-12, TNF-α, TNF-β
Mondelli et al., 2023	CHR vs HC	IFN-γ, IL-1β, IL-2, IL-4, IL-5, IL-6, IL-8, IL-10, IL12p70, IL-13, IL-15, TNF-α, TNF-β
Moreno et al., 2023	CHR vs HC	CRP, Fibrinogen, IL-6
Noyan et al., 2021	GHR vs HC	IL-1β
Ntouros et al., 2018	CHR vs HC	IFN-γ, IL-1β, IL-2, IL-4, IL-5, IL-8, IL-10, IL-12, IL-12p70, TNF-α, TNF-β,
Nunes et al., 2006	GHR vs HC	IL-6
Ouyang et al., 2022	CHR vs HC	IFN-γ, IL-1β, IL-2, IL-4, IL-6, IL-17, TNF-α, TNF-β,
Perkins et al., 2015	CHR vs HC	BDNF, CRP, Fibrinogen, IL-1β, IL-4, IL-5, IL-6, IL-8, IL-10, IL-12, IL-13, IL-15, TGF-β, TNF-α
Piotrowski et al., 2019	GHR vs HC	CRP, Fibrinogen
Rebouças et al., 2018	GHR vs HC	IL-6, TNF-α
Stojanovic et al., 2014	CHR vs HC	CRP, Fibrinogen, IL-6
Wang et al., 2020	GHR vs HC	IL-1β, IL-6, TNF-α
Wang et al., 2024	CHR vs HC	IL-1β, IL-2, IL-4, IL-6, IL-17
Yüksel et al., 2020	GHR vs HC	Gal-1, Gal-3
Zeni-Graiff et al., 2016	CHR vs HC	IFN-γ, IL-6, IL-17
Zhang et al., 2022	CHR vs HC	IL-1β, L-6
Zhang et al., 2024	CHR vs HC	IL-1β, IL-2, IL-6, IL-8, TNF-α
Zhang, Wei, et al., 2023	CHR vs HC	IL-2, IL-6

Zhang, Xiao, et al., 2023	CHR vs HC	IL-1β, IL-2, IL-6, IL-8, IL-10, TNF-α
Zhang, Zeng, et al., 2023	CHR vs HC	IL-1β, IL-2, IL-6, IL-8, IL-10, TNF-α

Table S8. List of the number of articles on the inflammatory markers.

Inflammatory markers	Comparison Group	Number of Studies
BDNF		***3***
	CHR vs HC	*2*
	GHR vs HC	*1*
CRP		***9***
	CHR vs HC	*6*
	GHR vs HC	*3*
Fibrinogen		***6***
	CHR vs HC	*5*
	GHR vs HC	*1*
IFN-γ		***8***
	CHR vs HC	*5*
	GHR vs HC	*3*
IL-1β		***15***
	CHR vs HC	*10*
	GHR vs HC	*5*
IL-2		***4***
	CHR vs HC	*4*

	GHR vs HC	*0*
IL-4		***7***
	CHR vs HC	*5*
	GHR vs HC	*2*
IL-5		***3***
	CHR vs HC	*3*
	GHR vs HC	*0*
IL-6		***23***
	CHR vs HC	*15*
	GHR vs HC	*8*
IL-8		***8***
	CHR vs HC	*7*
	GHR vs HC	*1*
IL-10		***9***
	CHR vs HC	*6*
	GHR vs HC	*3*
IL-12		***3***
	CHR vs HC	*2*
	GHR vs HC	*1*
IL-12p70		***3***
	CHR vs HC	*3*
	GHR vs HC	*0*

IL-13		***2***
	CHR vs HC	*2*
	GHR vs HC	*0*
IL-15		***2***
	CHR vs HC	*2*
	GHR vs HC	*0*
IL-17		***3***
	CHR vs HC	*3*
	GHR vs HC	*0*
TGF-β		***3***
	CHR vs HC	*1*
	GHR vs HC	*2*
TNF-α		***14***
	CHR vs HC	*8*
	GHR vs HC	*6*
TNF-β		***4***
	CHR vs HC	*3*
	GHR vs HC	*1*

Table S9. Table of included studies in pairwise meta-analysis for CHR and HC groups.

Study	Sample size		Inflammatory Markers in pairwise meta-analysis
	CHR	HC	
Kelsven et al., 2020	11	7	IL-12p70

Mondelli et al., 2023	269	56	IL-2, IL-5, IL-7, IL-12p70, IL-13, IL-15
Ntouros et al., 2018	12	23	IL-2, IL-5, IL-12p70
Ouyang et al., 2022	49	30	IL-2, IL-17
Perkins et al., 2015	72	35	IL-5, IL-7, IL-13, IL-15
Wang et al., 2024	34	14	IL-2, IL-17
Zeni-Graiff et al., 2016	12	16	IL-17
Zhang et al., 2024	385	95	IL-2
Zhang, Wei, et al., 2023	37	49	IL-2
Zhang, Xiao, et al., 2023	60	60	IL-2
Zhang, Zeng, et al., 2023	208	98	IL-2

Table S10. Table of included studies in pairwise meta-analysis for CHR-NT and CHR-T groups.

Study	Sample size		Follow-up time	Rate of transition	Inflammatory markers
	CHR-NT	CHR-T			
Labad et al., 2015	10	29	1 year	25.6	CRP, Fibrinogen
Mondelli et al., 2023	50	219	12 and 24 months	18.6	IL-1β, IL-2, IL-4, IL-6, IL-7, IL-8, IL-10, IL-13, IL-15, TNF-α
Perkins et al., 2015	32	40	2 years	44.4	CRP, IL-1β, IL-4, IL-6, IL-7, IL-8, IL-13, IL-15, TNF-α
Wang et al., 2024	22	12	2 years	35.3	IL-1β, IL-2, IL-4, IL-6, IL-17
Zhang et al., 2022	16	68	1 year	19	IL-1β, L-6
Zhang et al., 2024	88	297	1 year	22.9	IL-1β, IL-2, IL-6, IL-8, TNF-α
Zhang, Wei, et al., 2023	8	29	1 year	21.6	IL-2, IL-6
Zhang, Xiao, et al., 2023	15	45	2 years	25	IL-1β, IL-2, IL-6, IL-8, IL-10, TNF-α
Zhang, Zeng, et al., 2023	47	216	1 year	17.9	IL-1β, IL-2, IL-6, IL-8, IL-10, TNF-α

CHR-T： clinical high-risk transition to psychosis;

CHR-NT： clinical high-risk not transition to psychosis

Table S11. Egger's regression tests for publication bias.

Marker / Egger's (p)	network		Pairwise CHR-HC		Pairwise CHRT-NT	
	Studies	Egger's (p)	Studies	Egger's (p)	Studies	Egger's (p)
CRP	9	0.70	-	-	2	NA
TNF-α	14	0.93	-	-	5	0.26
TNF-β	4	0.85	-	-	-	-
IFN-γ	8	0.34	-	-	-	-
IL-2	-	-	8	**0.04**	6	0.52
IL-4	7	0.08	-	-	3	0.15
IL-5	-	-	3	0.13	2	NA
IL-6	23	0.41	-	-	8	0.83
IL-7	-	-	2	NA	2	NA
IL-8	8	0.13	-	-	5	0.21
IL-10	9	0.56	-	-	4	0.99
IL-1β	15	0.44	-	-	7	0.80
IL-12	3	0.43	-	-	-	-
IL-13	-	-	2	NA	2	NA
IL-15	-	-	2	NA	2	NA
IL-17	-	-	3	0.36	-	-
IL12p70	-	-	3	0.72	-	-
Fibrinogen	6	0.40	-	-	2	NA

BDNF	3	0.08	-	-	-	-
TGF-β	3	0.83	-	-	-	-

Egger's regression test was used to indicate possible publication bias. P-values beneath 0.05 were considered significant. Since the number of studies (k=2) was too small to test for small study effects, they were reported as 'NA'. "-" indicates that no data is analyzed.

Funnel plots were used to ascertain publication bias for markers with at least three studies.

Table S12. Network meta-analysis inconsistency results.

Cytokine	Number of studies included	I^2	Qwd	p (for Qwd)
CRP	9	7.1%	7.54	0.38
TNF-α	14	98.2%	657.78	**<0.0001**
TNF-β	4	80.8%	10.43	**0.01**
IFN-γ	8	77.3%	26.42	**0.0002**
IL-4	7	78.8%	23.63	**0.0003**
IL-6	23	95.5%	469.71	**<0.0001**
IL-8	8	56%	13.63	**0.0340**
IL-10	9	94.1%	119.04	**<0.0001**
IL-1β	15	97.8%	584.59	**<0.0001**
IL-12	3	54.1%	2.18	0.1398
Fibrinogen	6	0%	1.9	0.7551
BDNF	3	90%	9.96	**0.0016**
TGF-β	3	91.9%	12.29	**0.0005**

Qbd was the measure used to assess model inconsistency in the network meta-analysis between the three comparisons. For all of the cytokines, there were no multi-arm studies available – hence the Qbd and associated p-values didn't exist and reported.

Qwd was the heterogeneity within one design in the network meta-analysis. For TNF-α, Albumin and TGF-β, there was only one study in each design hence the Qwd and associated p-values were reported as ‘NA’.

Table S13. Bayesian Network Meta-Regression on age, gender, BMI, nicotine and medicine

Moderator / Marker	CRP		TNFα		TNFβ		IFNγ		IL4	
**age**	β: -0.58 (-1.31, 0.13)		β: 0.64 (-2.38, 3.31)		β: -0.68 (-1.70, 0.50)		β: 0.15 (-0.88, 1.19)		β: 0.14 (-1.47, 1.92)	
	No Covariate	Covariate age	No Covariate	Covariate age	No Covariate	Covariate age	No Covariate	Covariate age	No Covariate	Covariate age
	Dbar: 8.72	Dbar: 6.37	Dbar: 14.02	Dbar: 14.10	Dbar: 4.31	Dbar: 3.49	Dbar: 8.70	Dbar: 8.57	Dbar: 7.86	Dbar: 7.76
	pD: 4.29	pD: -12.31	pD: 13.75	pD: -125.55	pD: 3.81	pD: -4.17	pD: 7.08	pD: 6.04	pD: 6.55	pD: 5.75
	DIC: 13.01	DIC: -5.93	DIC: 27.77	DIC: -111.44	DIC: 8.12	DIC: -0.68	DIC: 15.77	DIC: 14.61	DIC: 14.41	DIC: 13.51
**gender**	β: 0.64 (-1.39, 4.61)		β: 4.54 (-0.02, 13.52)		β: -0.29 (-1.64, 1.00)		β: -0.49 (-1.66, 0.55)		β: 1.17 (0.19, 2.05)	
	No Covariate	Covariate gender	No Covariate	Covariate gender	No Covariate	Covariate gender	No Covariate	Covariate gender	No Covariate	Covariate gender
	Dbar: 8.72	Dbar: 8.43	Dbar: 14.02	Dbar: 14.06	Dbar: 4.31	Dbar: 4.22	Dbar: 8.70	Dbar: 8.89	Dbar: 7.86	Dbar: 6.47
	pD: 4.29	pD: -14.20	pD: 13.75	pD: -450337.88	pD: 3.81	pD: 3.07	pD: 7.08	pD: -0.44	pD: 6.55	pD: -27.94
	DIC: 13.01	DIC: -5.77	DIC: 27.77	DIC: -450323.82	DIC: 8.12	DIC: 7.29	DIC: 15.77	DIC: 8.45	DIC: 14.41	DIC: -21.47
	**IL-6**		**IL-8**		**IL-10**		**IL-1β**		**IL-12**	
**age**	β: 0.88 (-5.85, 3.47)		β: -0.21 (-1.26, 0.49)		β: -0.06 (-1.81, 1.78)		β: 0.20 (-1.59, 2.10)		β: -0.37 (-1.38, 0.53)	
	No Covariate	Covariate age	No Covariate	Covariate age	No Covariate	Covariate age	No Covariate	Covariate age	No Covariate	Covariate age
	Dbar:	Dbar:	Dbar:	Dbar:	Dbar:	Dbar: 9.05	Dbar:	Dbar:	Dbar:	Dbar: 3.00

	23.66	23.30	13.07	11.74	9.01		15.03	14.87	3.28	
	pD: 22.67	pD: -94.92	pD: 5.48	pD: 4.40	pD: 8.57	pD: 8.50	pD: 14.72	pD: 7.91	pD: 2.59	pD: 1.33
	DIC: 46.34	DIC: -71.61	DIC: 18.55	DIC: 16.13	DIC: 17.58	DIC: 17.55	DIC: 29.76	DIC: 22.78	DIC: 5.87	DIC: 4.32
**gender**	β: 0.27 (-1.52, 2.28)		β: 0.14 (-0.70, 0.86)		β: 0.27 (-1.23, 1.73)		β: -0.35 (-1.90, 1.19)		β: -0.40 (-1.65, 0.60)	
	No Covariate	Covariate gender	No Covariate	Covariate gender	No Covariate	Covariate gender	No Covariate	Covariate gender	No Covariate	Covariate gender
	Dbar: 23.66	Dbar: 23.66	Dbar: 13.07	Dbar: 12.97	Dbar: 9.01	Dbar: 9.07	Dbar: 15.03	Dbar: 14.67	Dbar: 3.28	Dbar: 2.88
	pD: 22.67	pD: 15.52	pD: 5.48	pD: 5.78	pD: 8.57	pD: 6.89	pD: 14.72	pD: 5.86	pD: 2.59	pD: 1.14
	DIC: 46.34	DIC: 39.18	DIC: 18.55	DIC: 18.74	DIC: 17.58	DIC: 15.96	DIC: 29.76	DIC: 20.53	DIC: 5.87	DIC: 4.02

Continued:

Moderator / Marker	**Fibrinogen**		**BDNF**		**TGFβ**	
**age**	β: -0.02 (-0.77, 0.71)		β: -3.70 (-17.64, 4.18)		β: 0.90 (-4.22, 10.87)	
	No Covariate	Covariate age	No Covariate	Covariate age	No Covariate	Covariate age
	Dbar: 3.94	Dbar: 4.41	Dbar: 3.32	Dbar: 3.17	Dbar: 3.29	Dbar: 3.14
	pD: 2.95	pD: 3.43	pD: 3.06	pD: -96.41	pD: 3.07	pD: -9.52
	DIC: 6.90	DIC: 7.84	DIC: 6.38	DIC: -93.24	DIC: 6.36	DIC: -6.38
**gender**	β: 0.13 (-0.66, 1.09)		β: 3.25 (-7.26, 28.64)		β: -0.86 (-6.61, 2.60)	
	No Covariate	Covariate gender	No Covariate	Covariate gender	No Covariate	Covariate gender

	Dbar: 3.94	Dbar: 4.02	Dbar: 3.32	Dbar: 3.21	Dbar: 3.29	Dbar: 3.16
	pD: 2.95	pD: 2.64	pD: 3.06	pD: -81.53	pD: 3.07	pD: -8.58
	DIC: 6.90	DIC: 6.66	DIC: 6.38	DIC: -78.31	DIC: 6.36	DIC: -5.42
**medicine**	β: -0.03 (-0.63, 0.52)		β: -1.45 (-5.01, 1.97)			
	No Covariate	Covariate medicine	No Covariate	Covariate medicine		
	Dbar: 3.94	Dbar: 4.40	Dbar: 3.32	Dbar: 2.96	-	
	pD: 2.95	pD: 3.48	pD: 3.06	pD: -18.62		
	DIC: 6.90	DIC: 7.88	DIC: 6.38	DIC: -15.65		

Network meta regression was analyzed based on the model using gemtc package.
DIC: deviance information criterion; Dbar: posterior mean of the residual deviance; pD: the leverage of the residual deviance; β: Regression Coefficient. Significance shown in red.

Table S14. Meta regression in pairwise meta-analysis (CHR vs HC).

Moderator / Marker	**IL-2**		**IL-5**		**IL-17**		**IL-12p70**	
	β	*p*	β	*p*	β	*p*	β	*p*
age	-0.05	0.40	-0.01	0.98	0.69	0.58	-0.05	0.78
gender	0.01	0.78	-0.01	0.11	-0.14	**0.03**	-0.02	0.25
education	-	-	-	-	-22.18	0.69	-	-

*BOLD indicates significance.

Table S15. Meta regression in pairwise meta-analysis (CHR-T vs CHR-NT).

Moderator / Marker	**TNFα**		**IL-4**		**IL-6**		**IL-8**	
	β	*p*	β	*p*	β	*p*	β	*p*
age	0.02	0.51	0.09	0.24	-0.03	0.46	0.04	0.67

gender	0.02	0.19	-0.03	0.59	-0.02	0.25	0.05	0.18
	**IL-1β**		**IL-2**		**IL-10**			
	β	*p*	β	*p*	β	*p*		
age	-0.02	0.76	0.05	0.72	0.04	0.31		
gender	0.03	0.42	0.01	0.96	0.03	0.55		
education	-	-	0.17	0.48	0.09	0.28		

*BOLD indicates significance.

Figure S1. Transitivity assessments of NMA analysis.

1.Age

**CRP**	Min.	1st Qu	Median	Mean	3rd Qu	Max.
	19.60	22.98	23.51	24.99	24.91	32.06

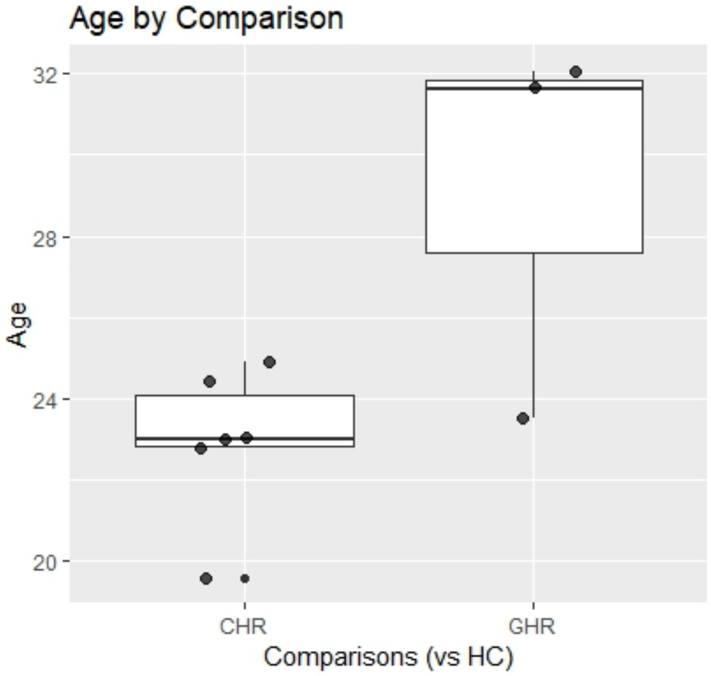

**Fibrinogen**	Min.	1st Qu	Median	Mean	3rd Qu	Max.
	19.60	22.83	23.01	24.23	24.45	32.06

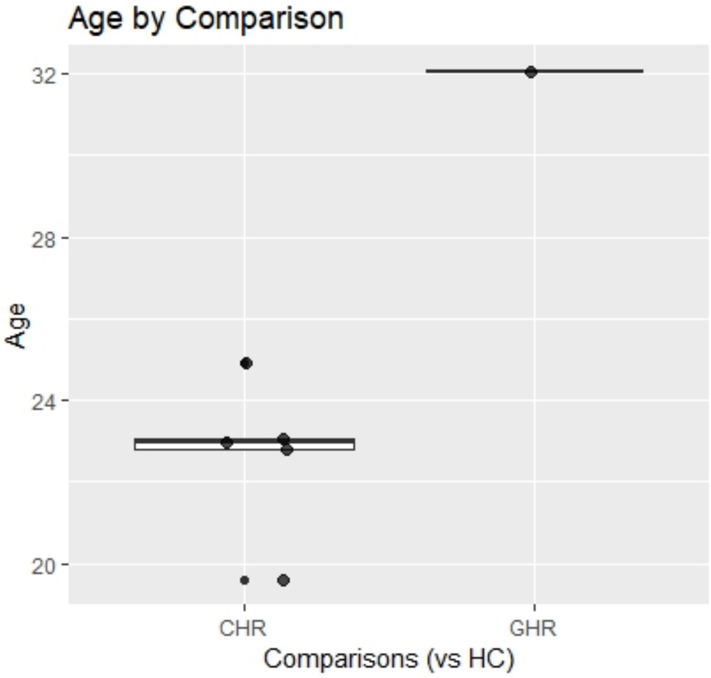

**IFN-γ**	Min.	1st Qu	Median	Mean	3rd Qu	Max.
	18.27	19.08	23.50	24.01	27.42	31.64

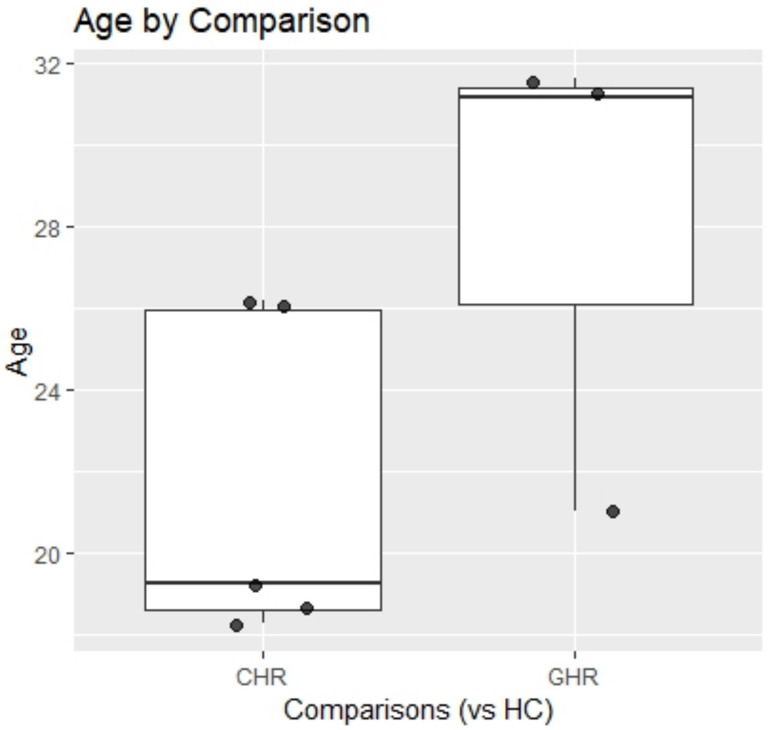

**IL-1β**	Min.	1st Qu	Median	Mean	3rd Qu	Max.
	18.58	19.06	20.25	23.07	26.07	34.56

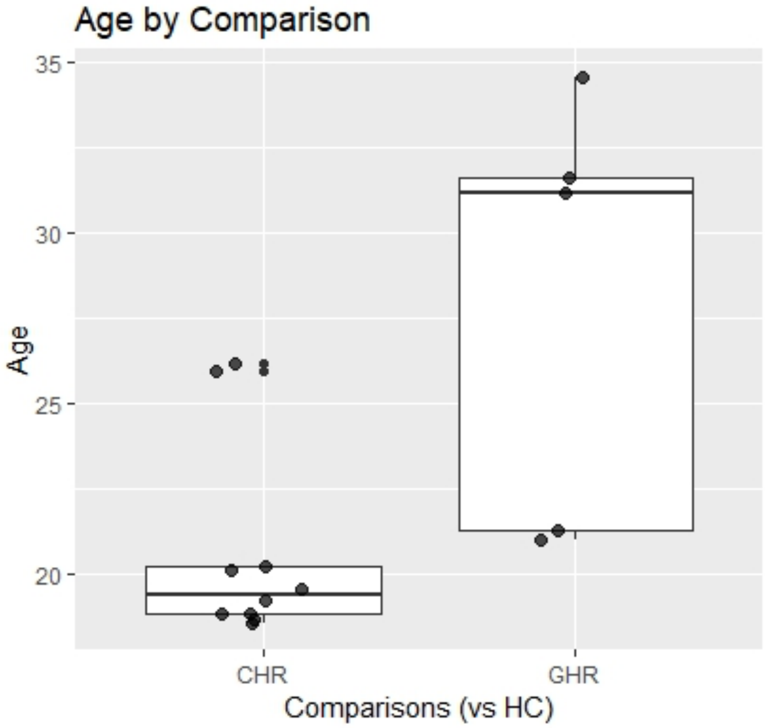

**IL-4**	Min.	1st Qu	Median	Mean	3rd Qu	Max.
	18.58	19.87	25.97	24.76	28.68	31.64

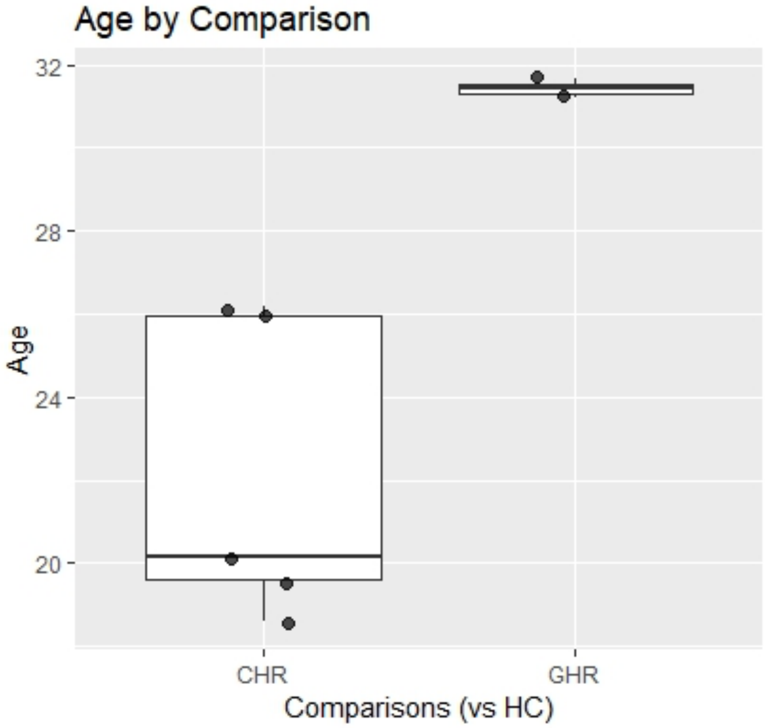

**IL-6**	Min.	1st Qu	Median	Mean	3rd Qu	Max.
	18.00	18.97	21.16	23.25	24.79	40.78

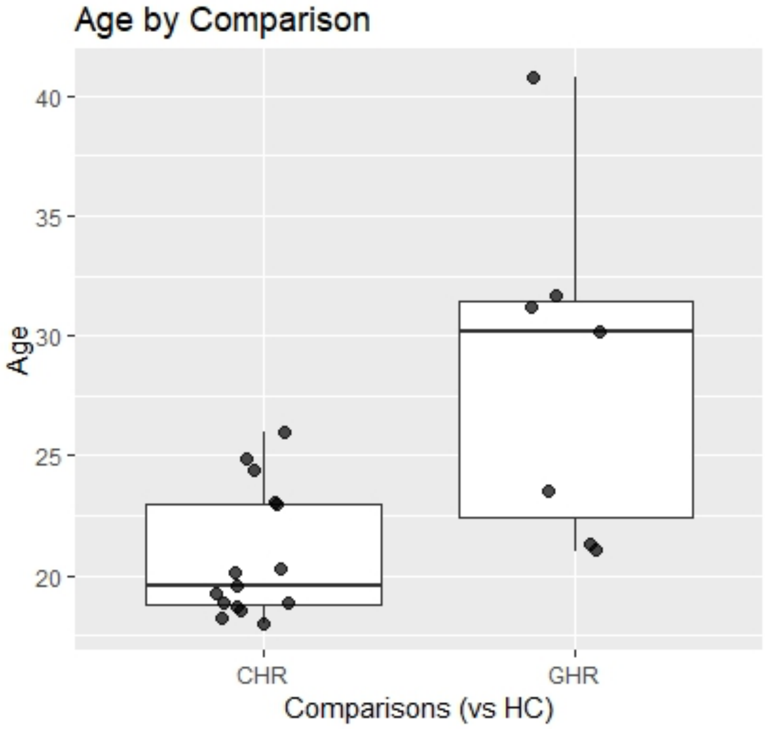

**IL-8**	Min.	1st Qu	Median	Mean	3rd Qu	Max.
	18.83	19.15	19.92	21.25	22.27	26.17

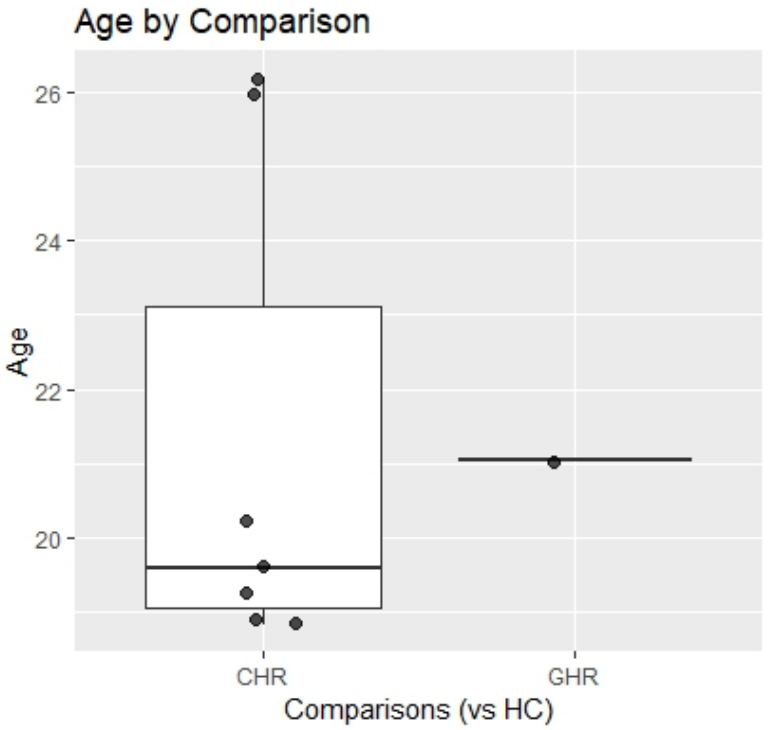

**IL-10**	Min.	1st Qu	Median	Mean	3rd Qu	Max.
	18.88	19.60	21.03	23.77	26.17	31.64

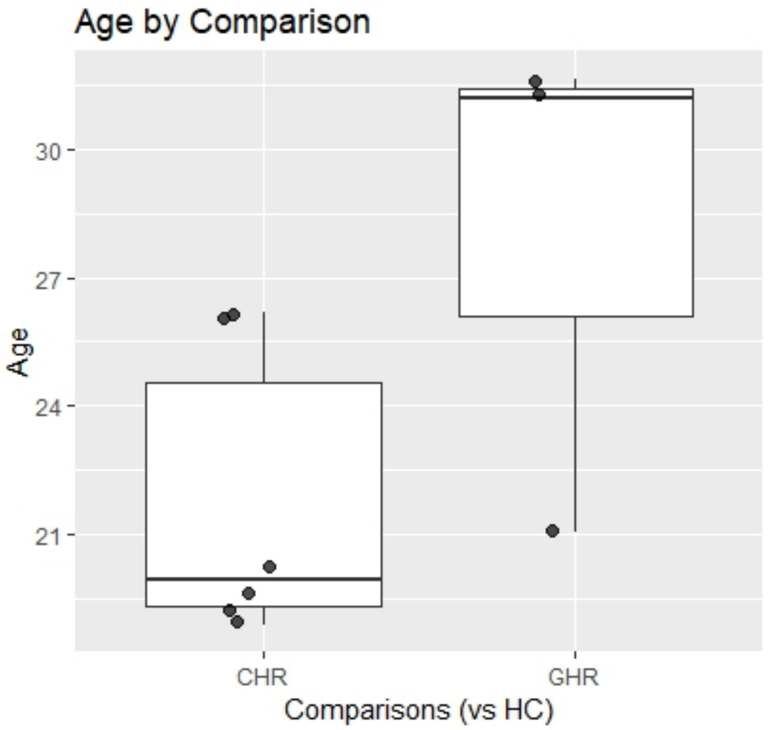

**TNF-α**	Min.	1st Qu	Median	Mean	3rd Qu	Max.
	18.58	19.33	21.16	24.07	26.12	40.78

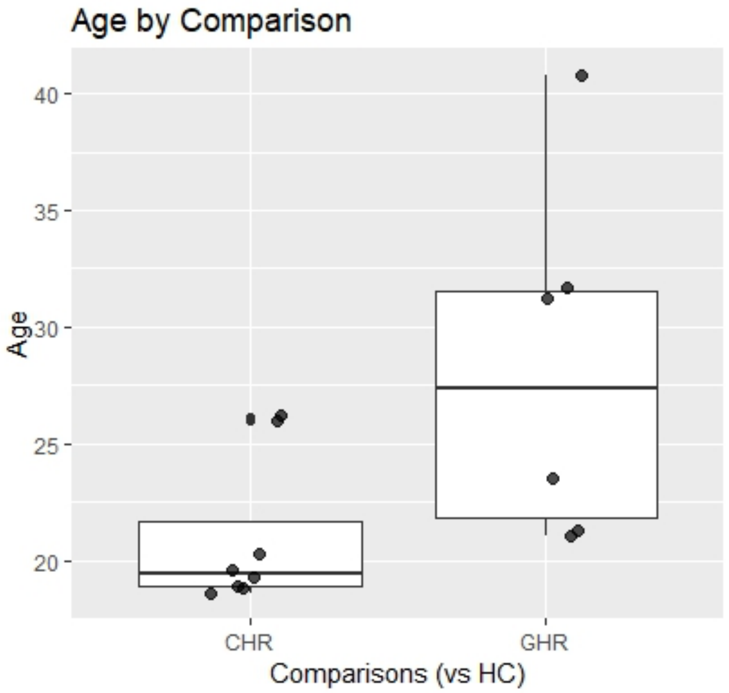

2. Gender

**CRP**	Min.	1st Qu	Median	Mean	3rd Qu	Max.
	35.43	44.93	57.15	53.83	65.79	31.64

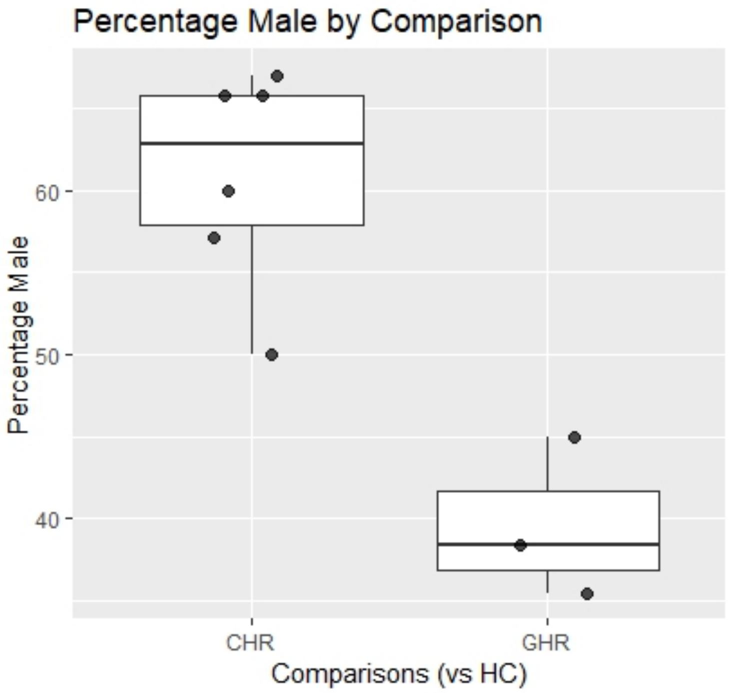

**Fibrinogen**	Min.	1st Qu	Median	Mean	3rd Qu	Max.
	35.43	57.86	62.89	58.53	65.80	67.03

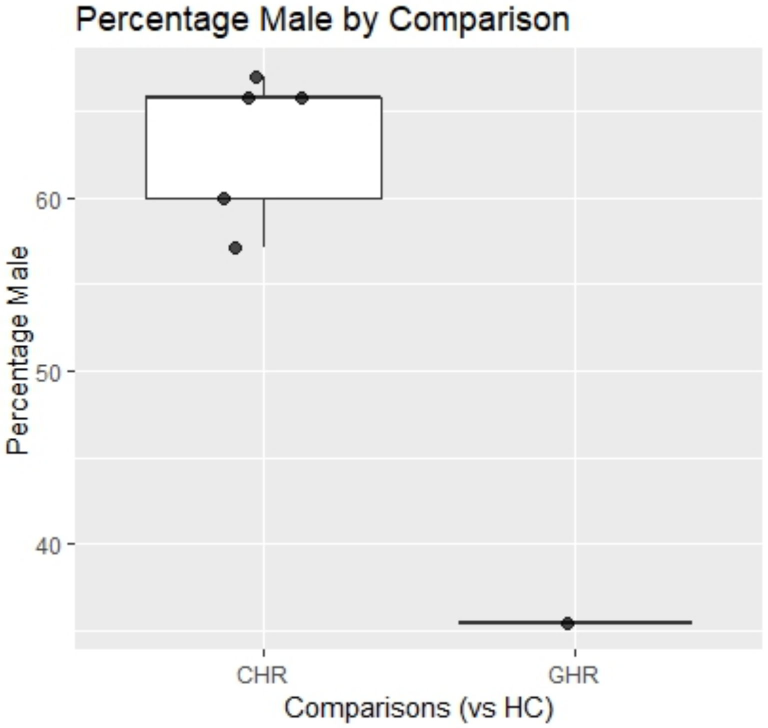

**IFN-γ**	Min.	1st Qu	Median	Mean	3rd Qu	Max.
	44.93	52.45	55.70	62.03	67.86	100.00

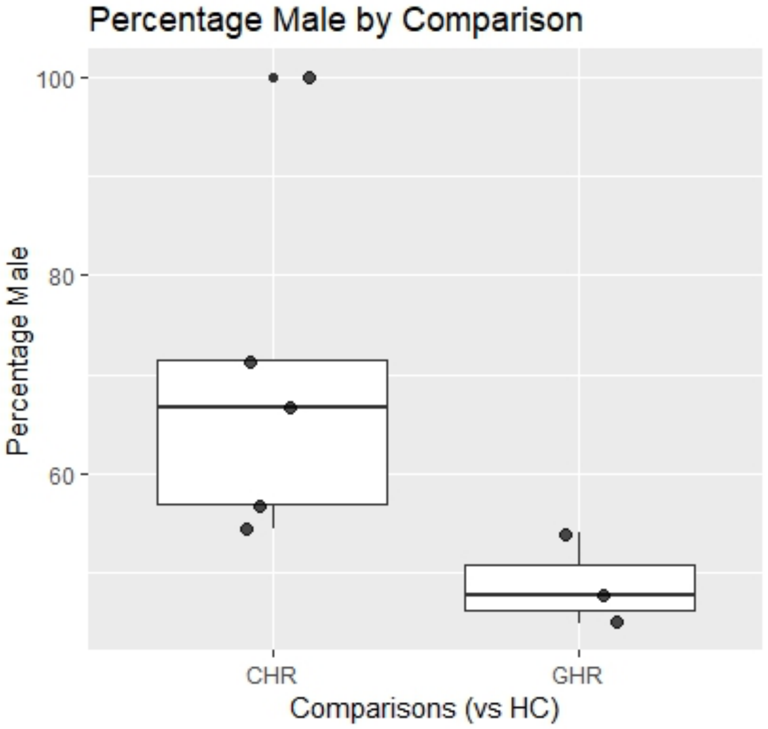

**IL-1β**	Min.	1st Qu	Median	Mean	3rd Qu	Max.
	36.38	48.55	53.68	55.37	56.44	100.00

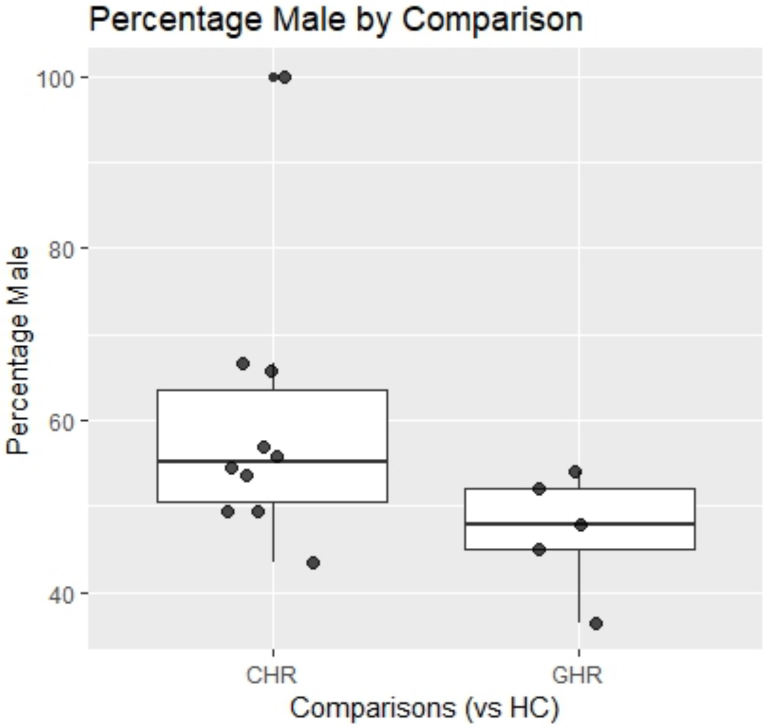

**IL-4**	Min.	1st Qu	Median	Mean	3rd Qu	Max.
	44.93	51.08	55.90	60.82	61.39	100.00

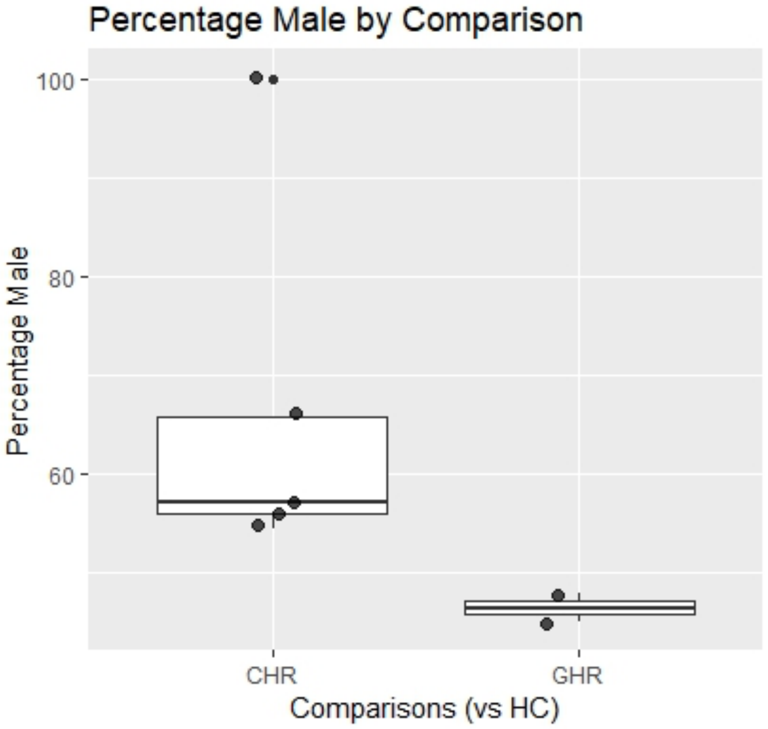

IL-6

Min.	1st Qu	Median	Mean	3rd Qu	Max.
38.35	49.37	53.85	54.28	59.28	71.46

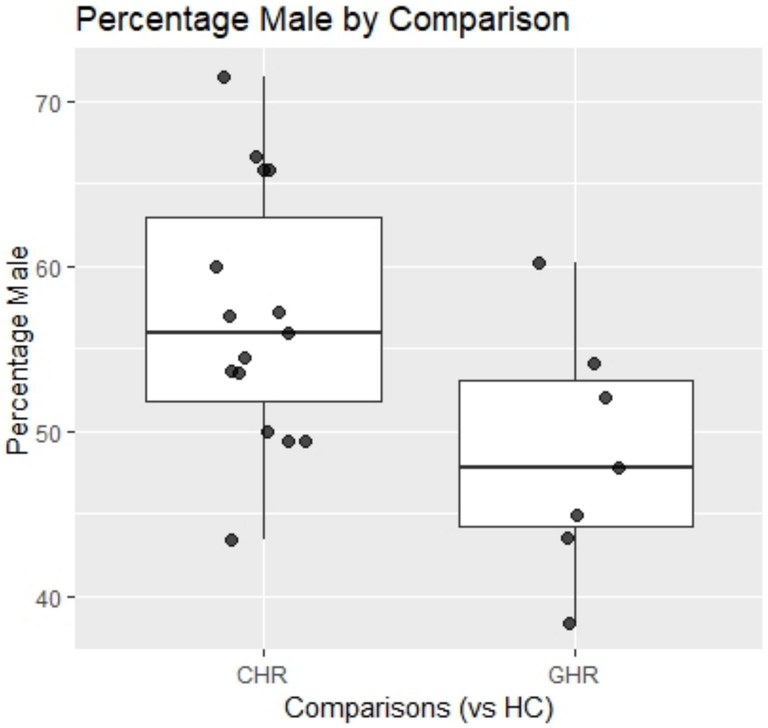

IL-8

Min.	1st Qu	Median	Mean	3rd Qu	Max.
43.35	49.37	54.23	60.38	66.01	100.00

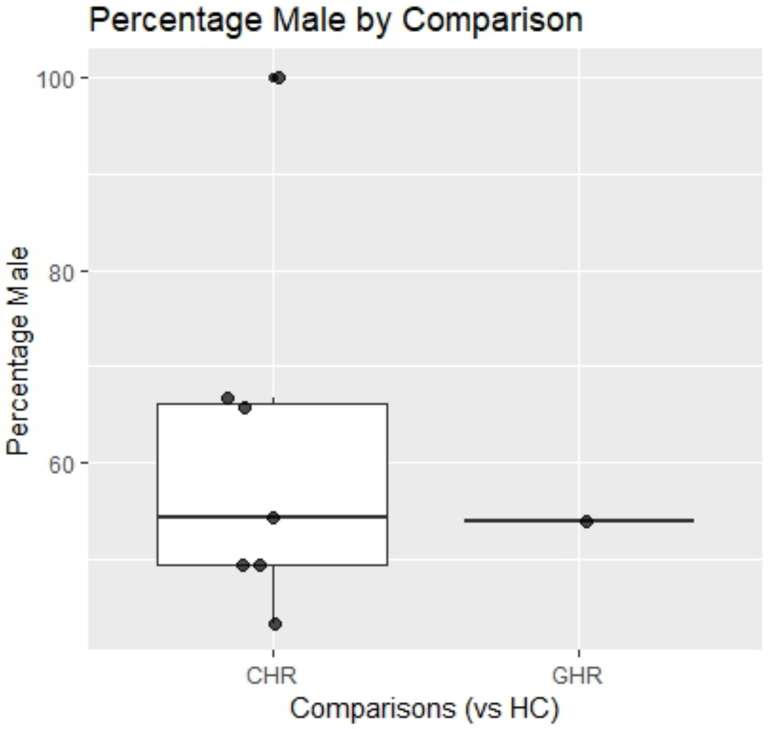

**IL-10**	Min.	1st Qu	Median	Mean	3rd Qu	Max.
	43.35	47.74	49.37	56.65	54.43	100.00

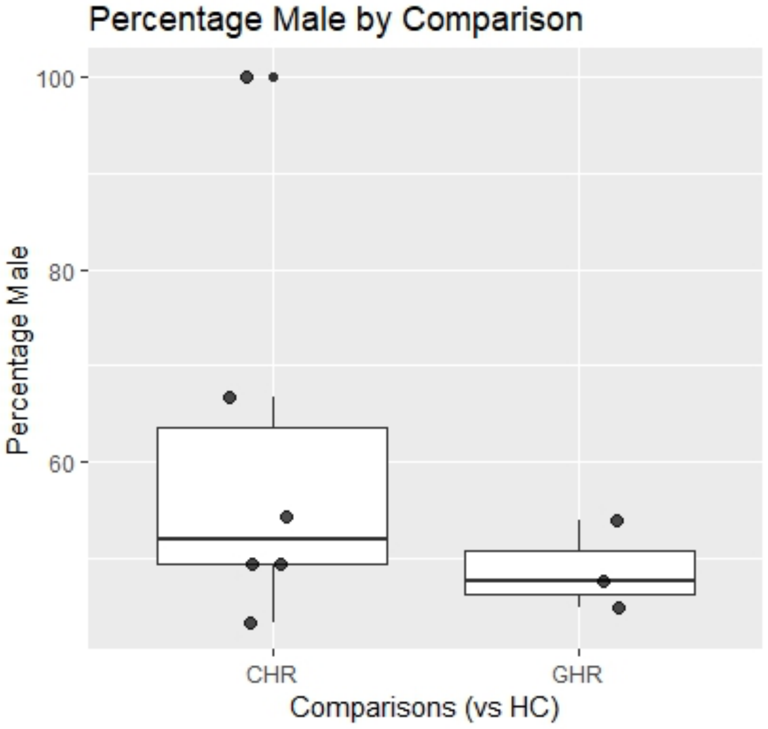

**TNF-α**	Min.	1st Qu	Median	Mean	3rd Qu	Max.
	38.35	45.63	50.68	54.75	56.34	100.00

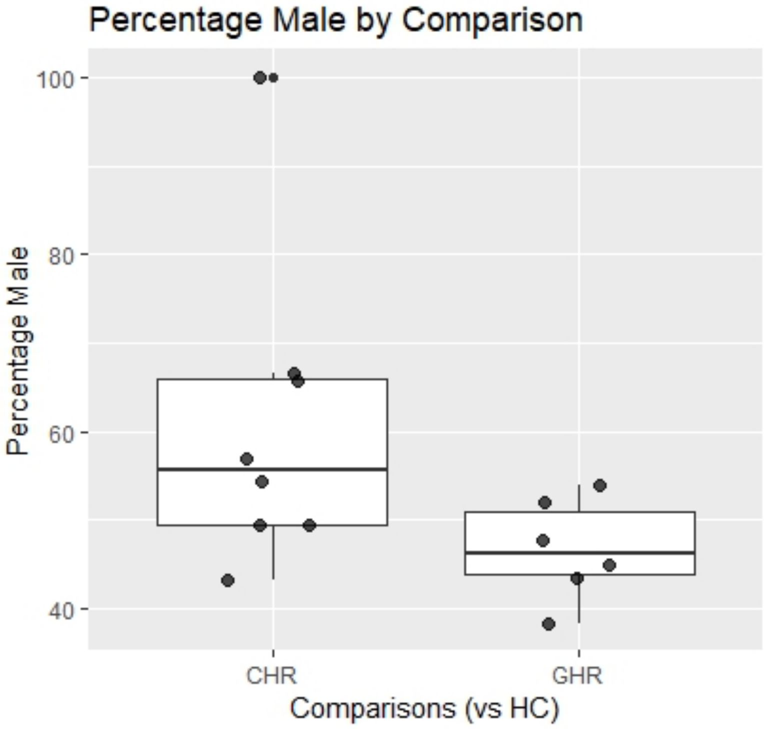

3. Antipsychotics

**CRP**	Min.	1st Qu	Median	Mean	3rd Qu	Max.
	0.0000	0.0000	0.1900	0.1812	0.2950	0.4700

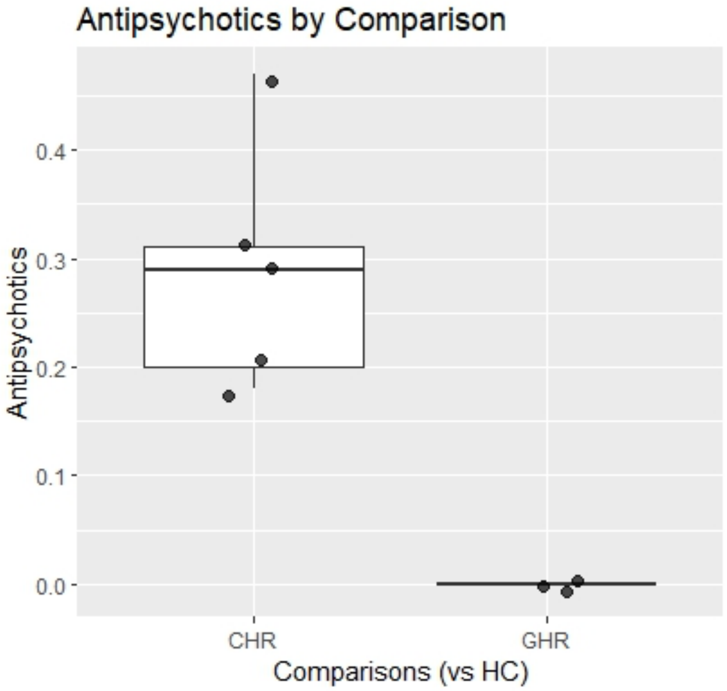

**Fibrinogen**	Min.	1st Qu	Median	Mean	3rd Qu	Max.
	0.0000	0.1850	0.2450	0.2417	0.3050	0.4700

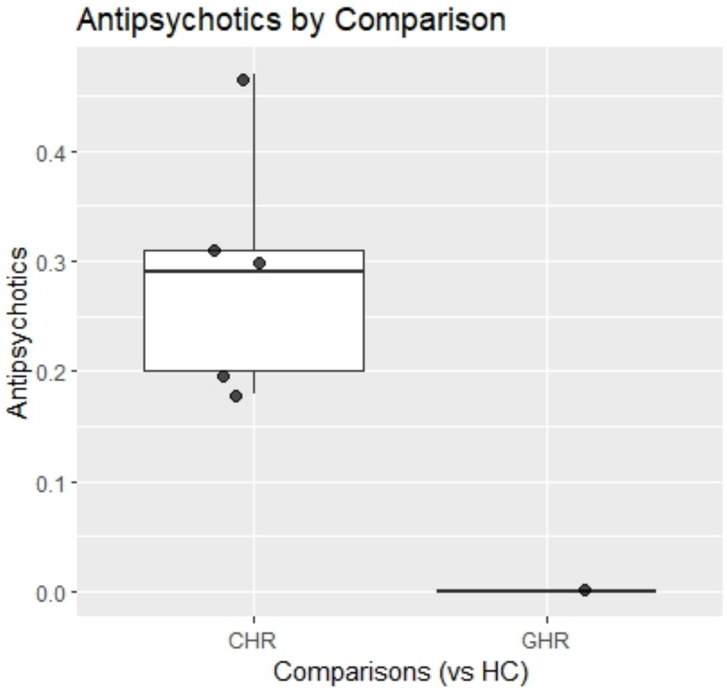

IFN-γ	Min.	1st Qu	Median	Mean	3rd Qu	Max.
	0.00	0.00	0.00	0.06	0.00	0.42

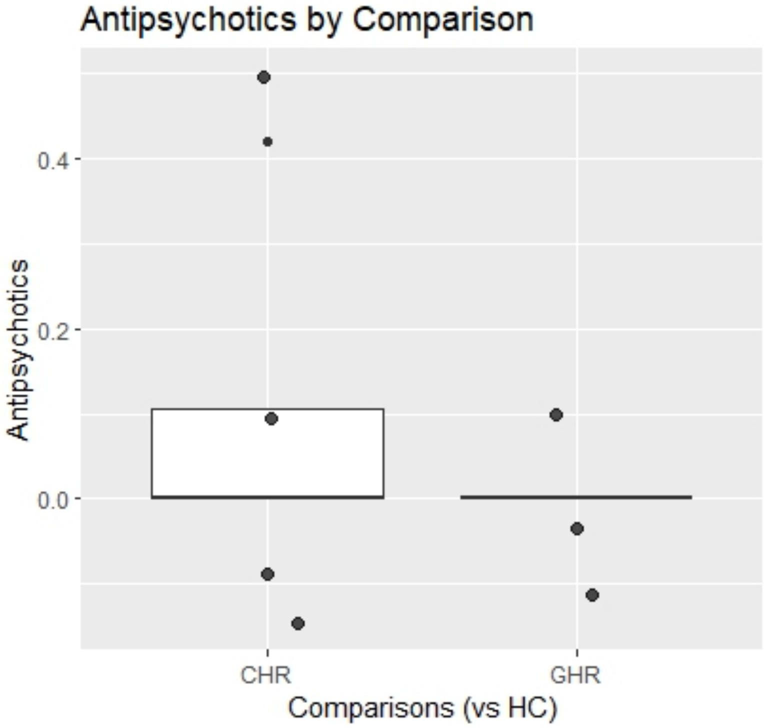

IL-1β	Min.	1st Qu	Median	Mean	3rd Qu	Max.
	0.0000	0.0000	0.0000	0.025	0.0000	0.200

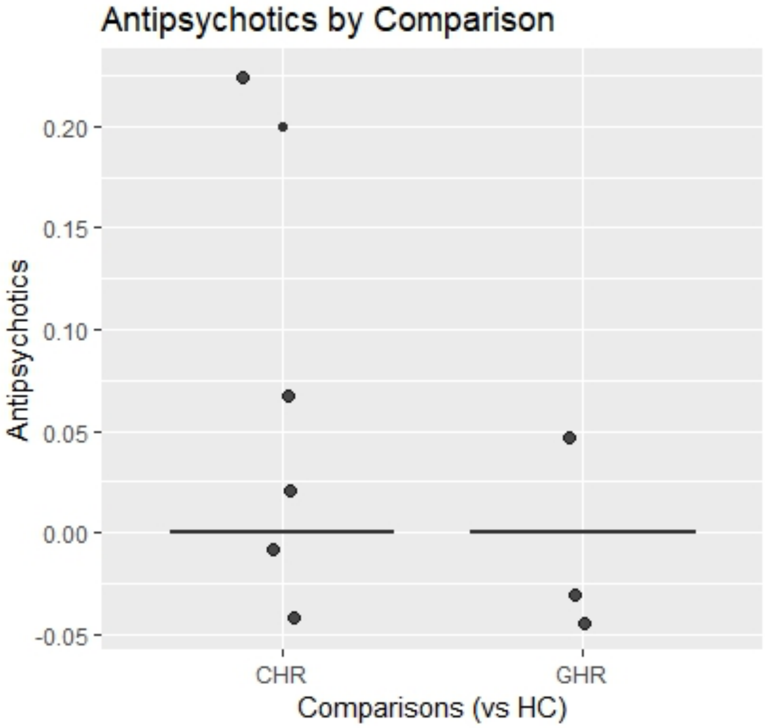

IL-4

Min.	1st Qu	Median	Mean	3rd Qu	Max.
0.00	0.00	0.00	0.04	0.00	0.20

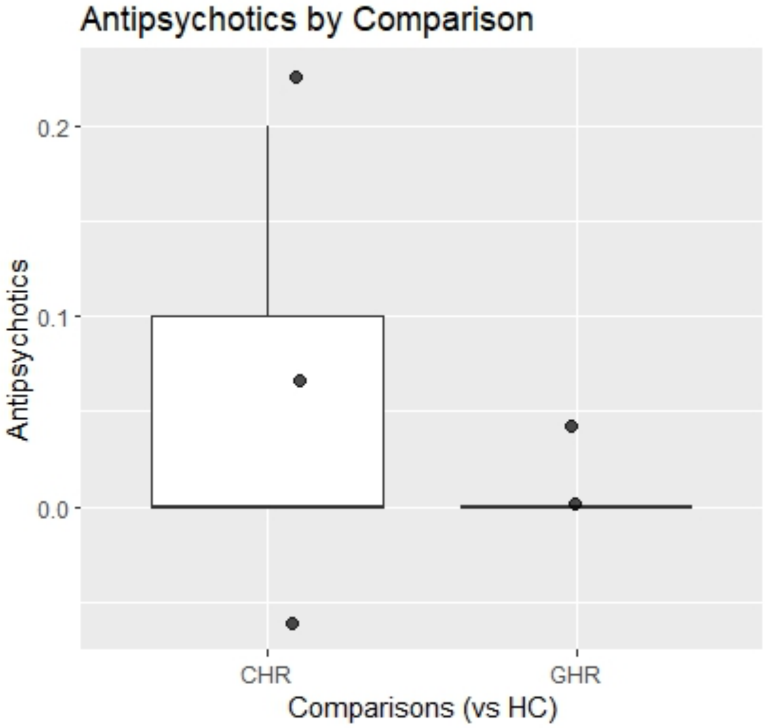

IL-6

Min.	1st Qu	Median	Mean	3rd Qu	Max.
0.0000	0.0000	0.0000	0.1207	0.2675	0.4700

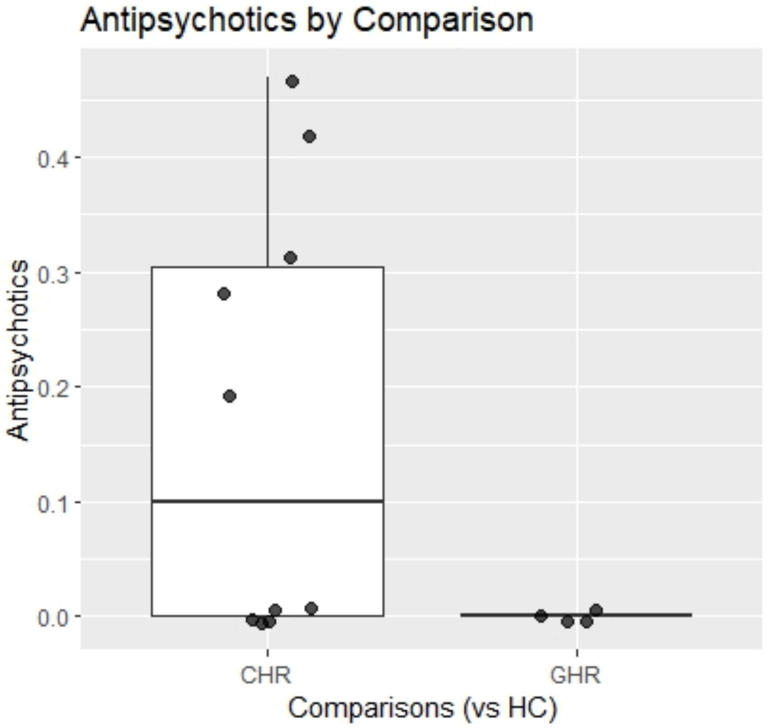

**IL-8**	Min.	1st Qu	Median	Mean	3rd Qu	Max.
	0.0000	0.00	0.00	0.05	0.05	0.20

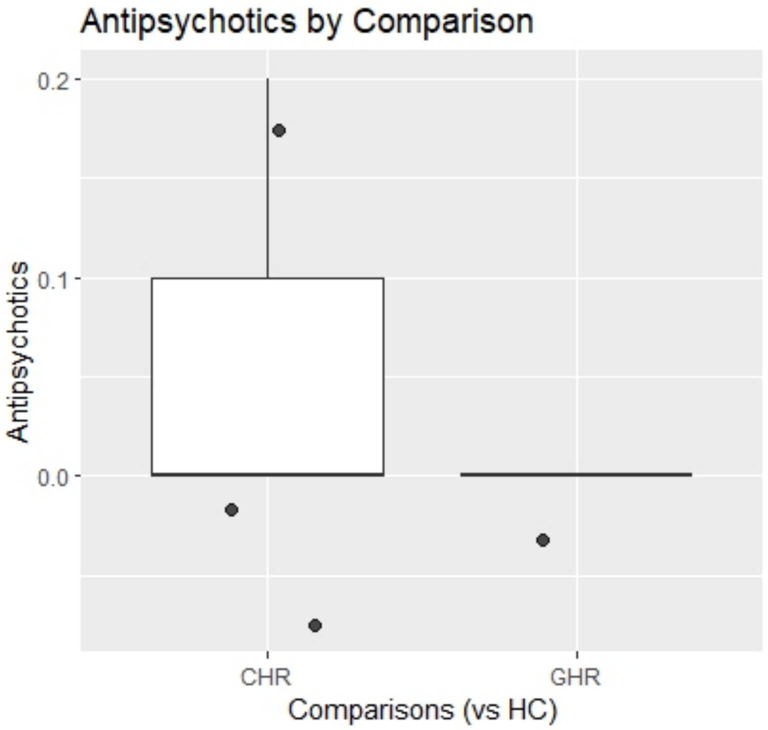

**IL-10**	Min.	1st Qu	Median	Mean	3rd Qu	Max.
	0.0000	0.0000	0.0000	0.03333	0.0000	0.2000

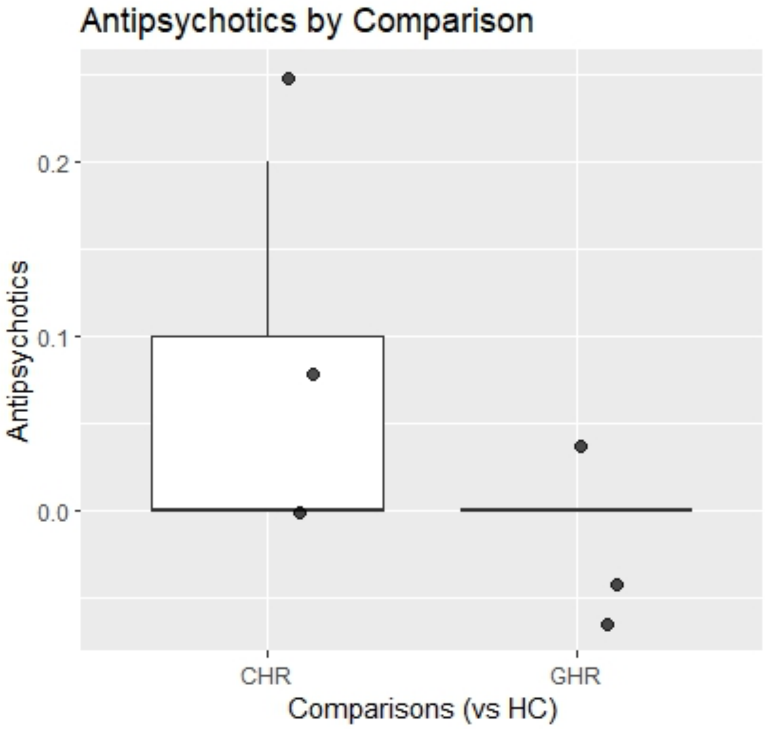

**TNF-α**	Min.	1st Qu	Median	Mean	3rd Qu	Max.
	0.0000	0.1850	0.2450	0.2417	0.3050	0.4700

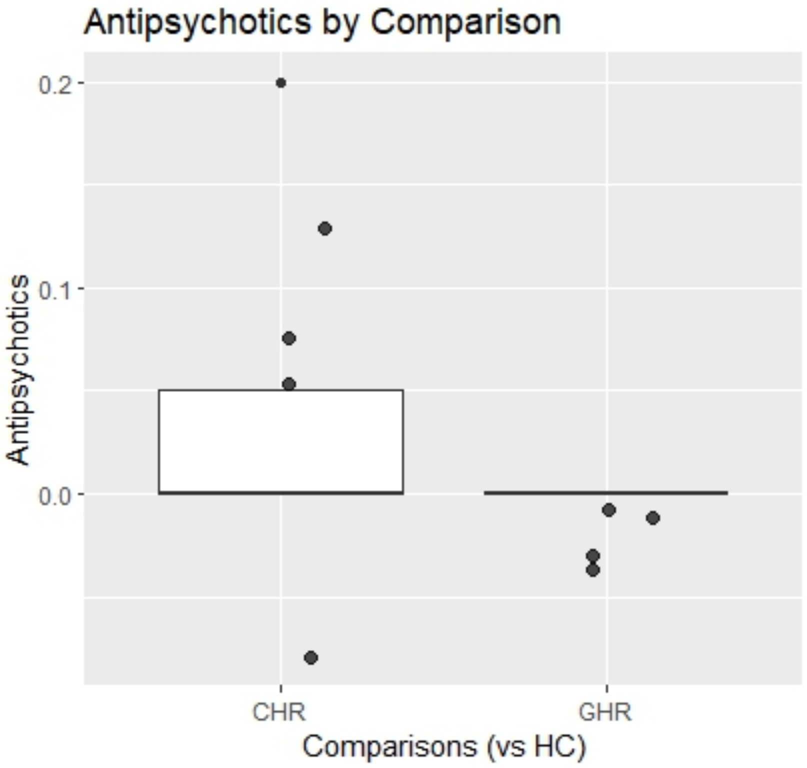

Note:

Min=Minimum; 1st Qu=First Quartile; 3rd Qu=Third Quartile; Max=Maximum

Figure S2. Network Geometry Plots.

BNDF:

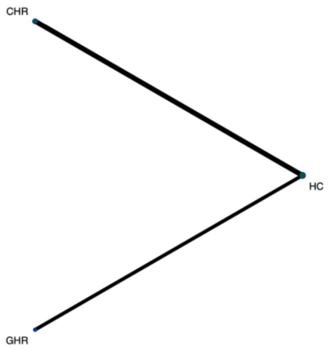

CRP:

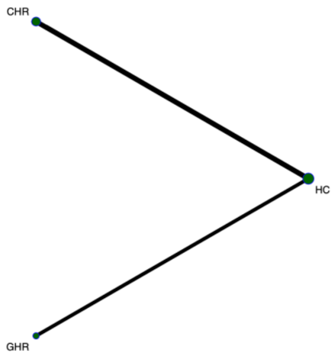

Fibrinogen:

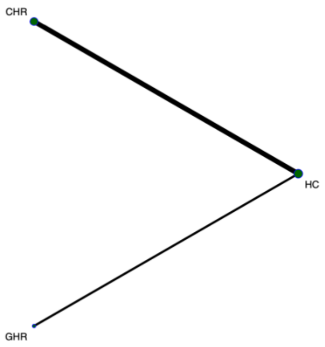

IFN-γ:

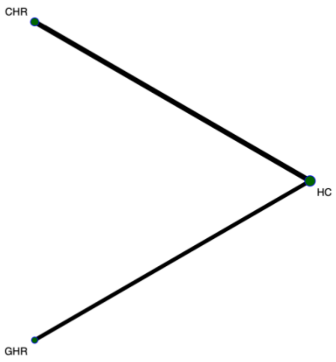

IL-1β:

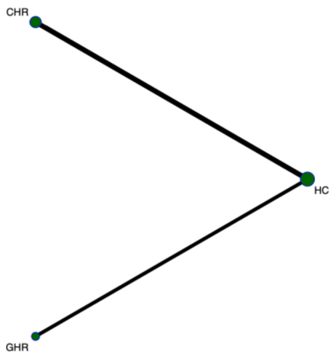

IL-4:

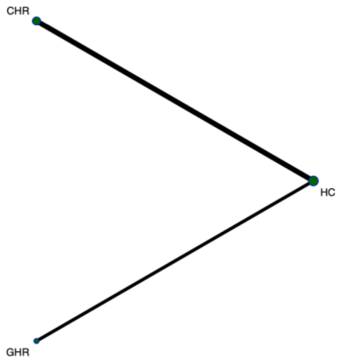

IL-6:

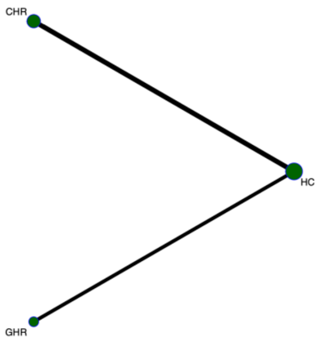

IL-8:

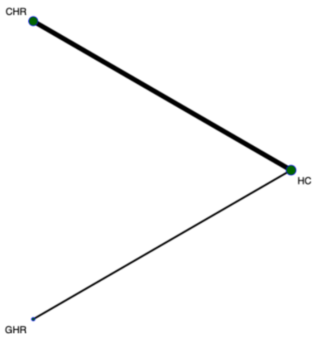

IL-10:

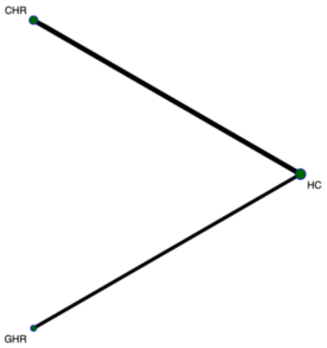

IL-12：

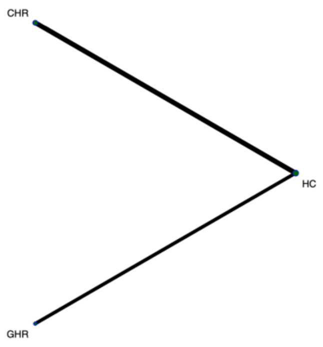

TGF-β：

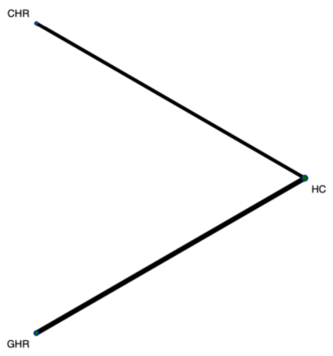

TNF-α：

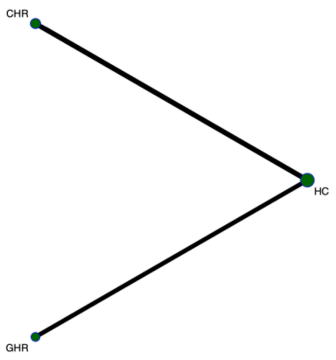

TNF-β：

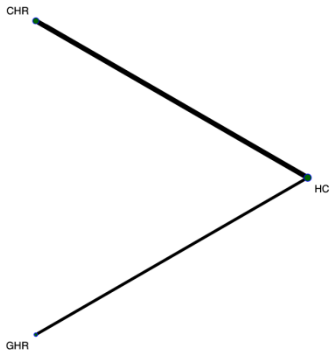

Figure S3. Funnel plots of NMA analysis.

BDNF

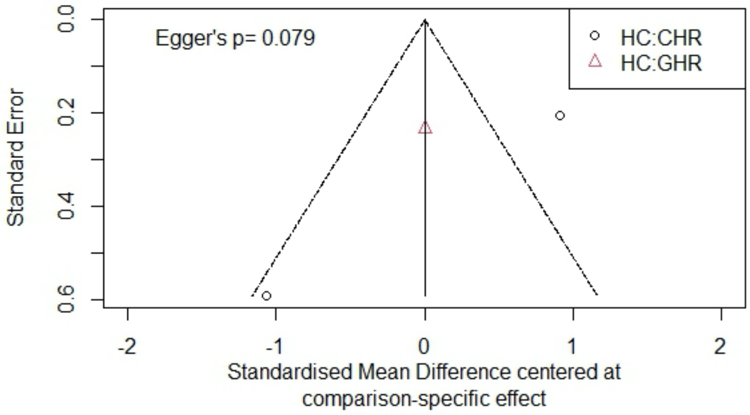

CRP

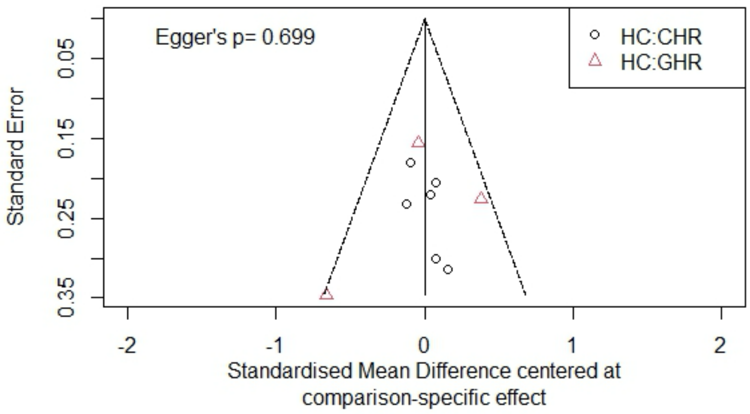

Fibrinogen

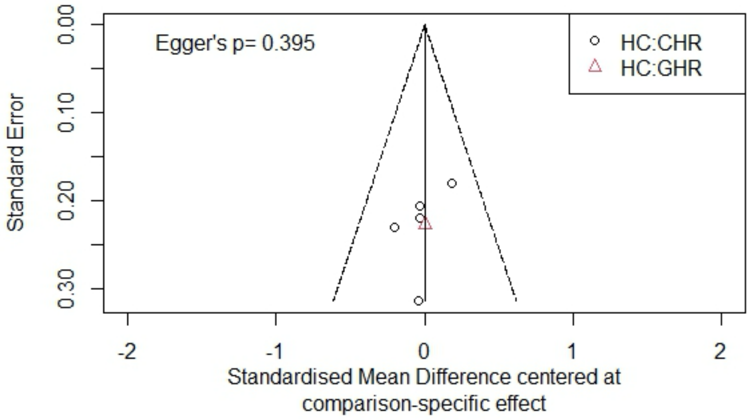

IFN-γ

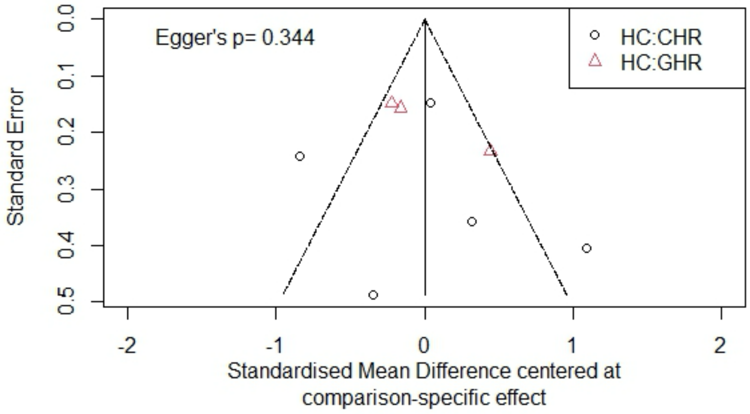

IL-1β

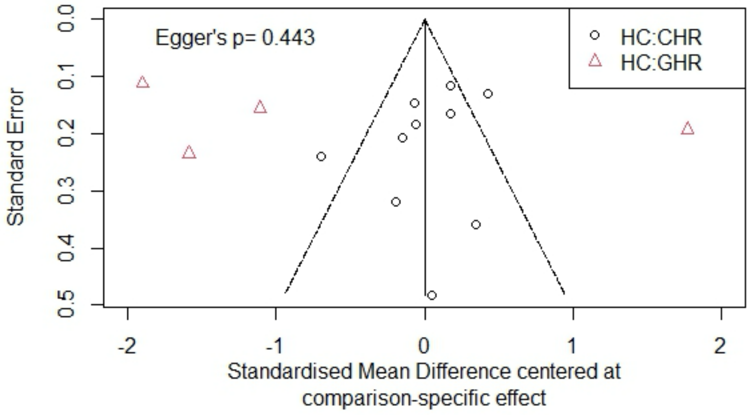

IL-4

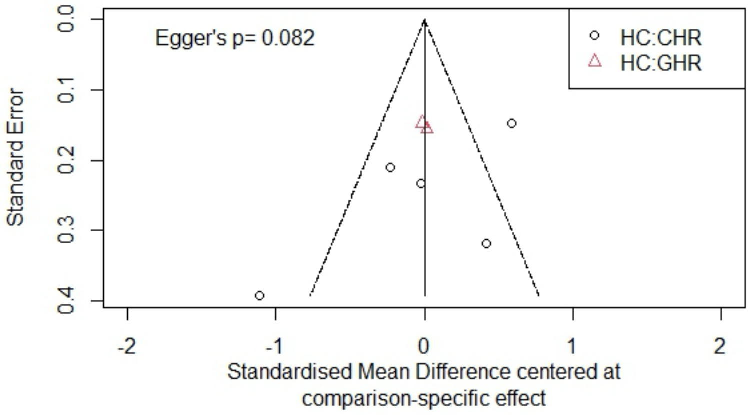

IL-6

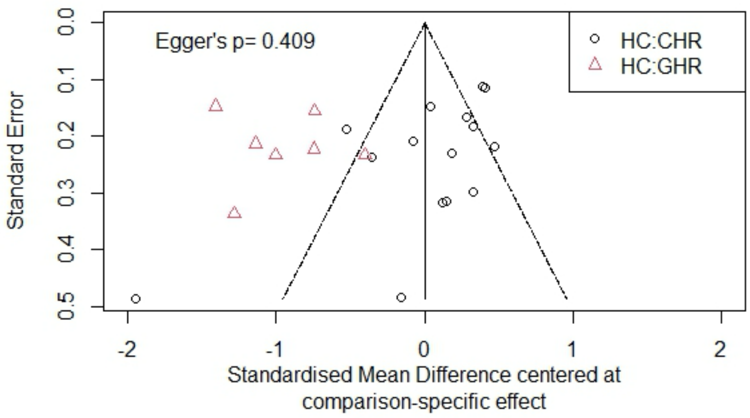

Egger's p= 0.409
HC:CHR
HC:GHR
Standard Error
0.0
0.1
0.2
0.3
0.4
0.5
-2
-1
0
1
2
Standardised Mean Difference centered at
comparison-specific effect

IL-8

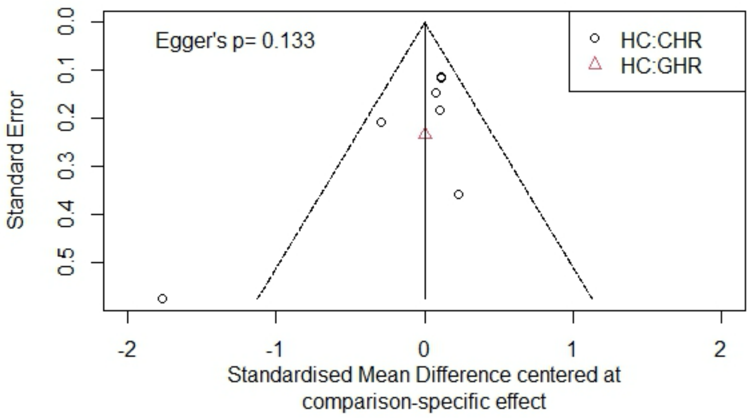

Egger's p= 0.133
HC:CHR
HC:GHR
Standard Error
0.0
0.1
0.2
0.3
0.4
0.5
-2
-1
0
1
2
Standardised Mean Difference centered at
comparison-specific effect

IL-10

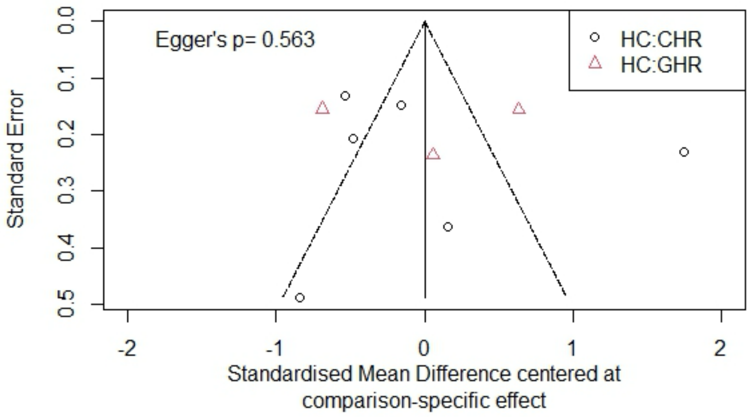

Egger's p= 0.563
HC:CHR
HC:GHR
Standard Error
0.0
0.1
0.2
0.3
0.4
0.5
-2
-1
0
1
2
Standardised Mean Difference centered at
comparison-specific effect

IL-12

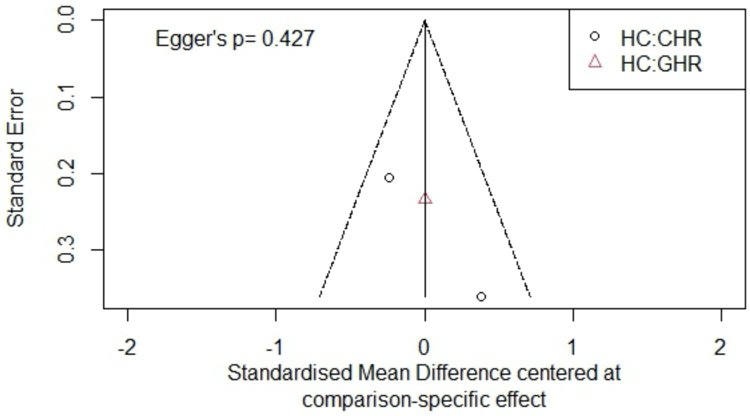

TGF-β

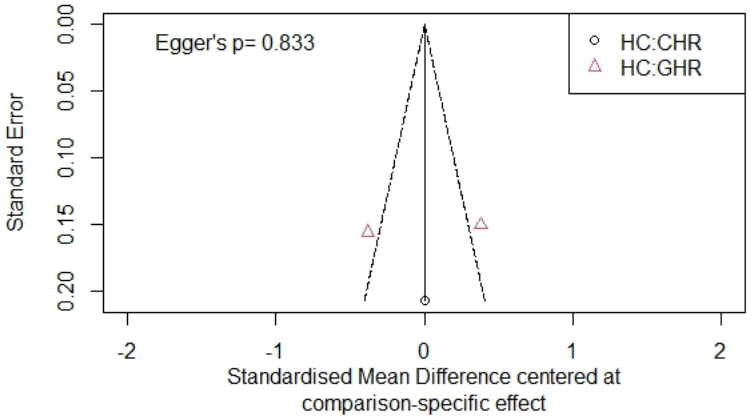

TNF-α

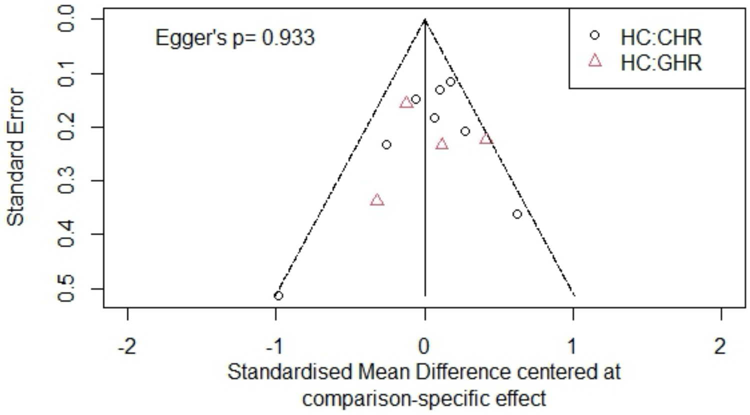

TNF-β

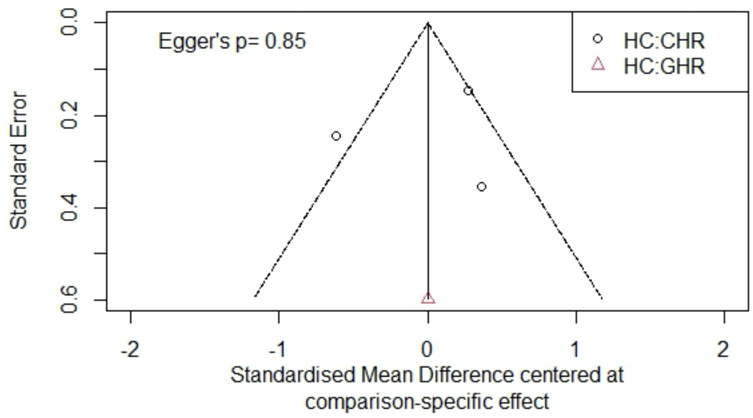

Figure S4. Pairwise meta-analysis results of comparison of makers between CHR and HC groups.

IL-2

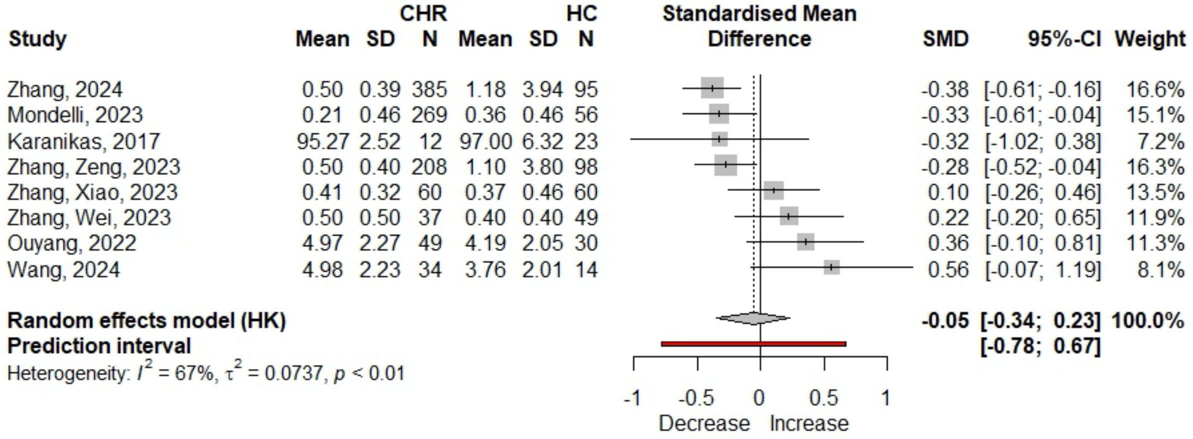

IL-5

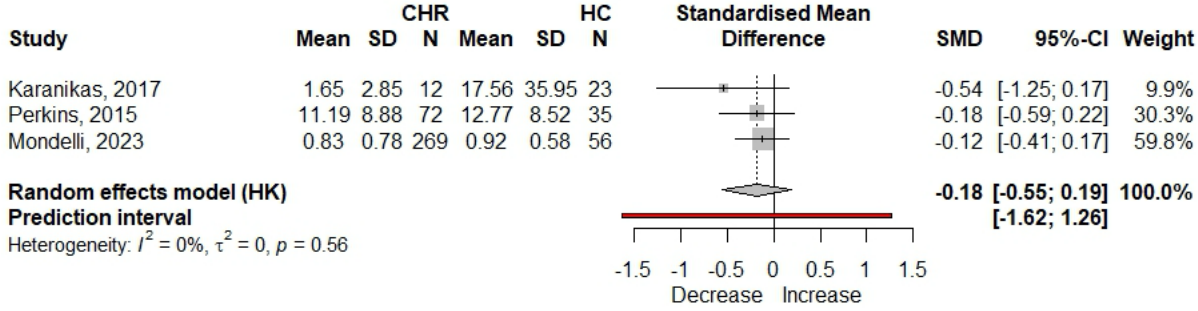

IL-7

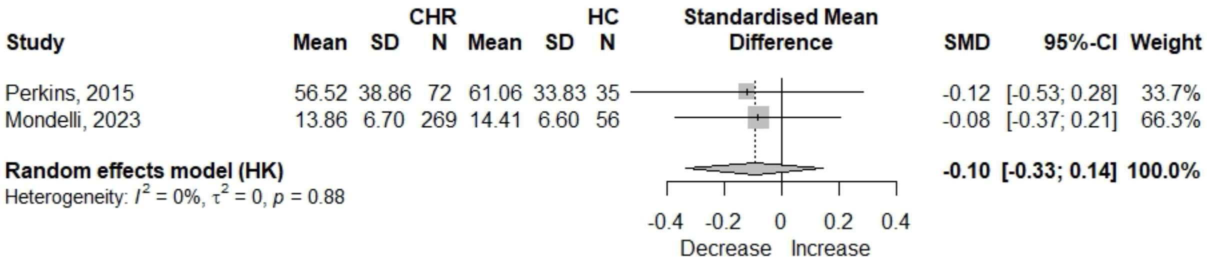

IL-

12P70

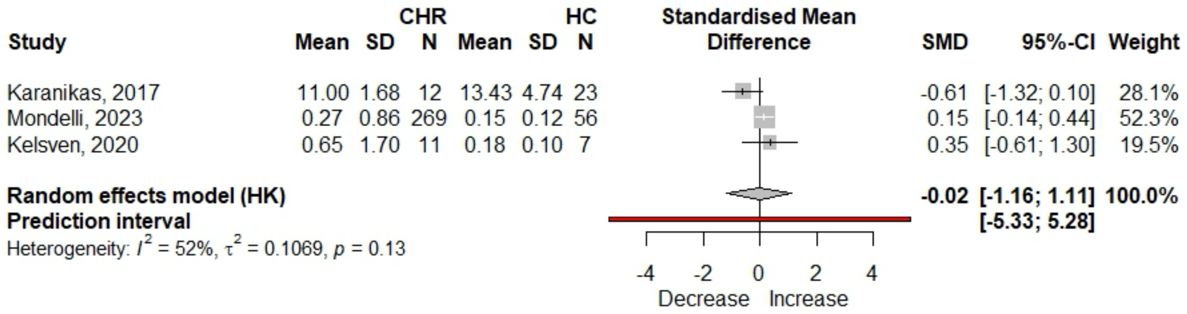

IL-13

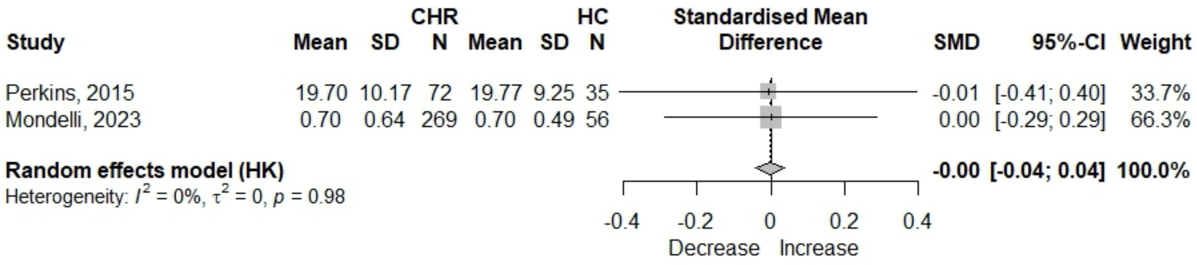

IL-15

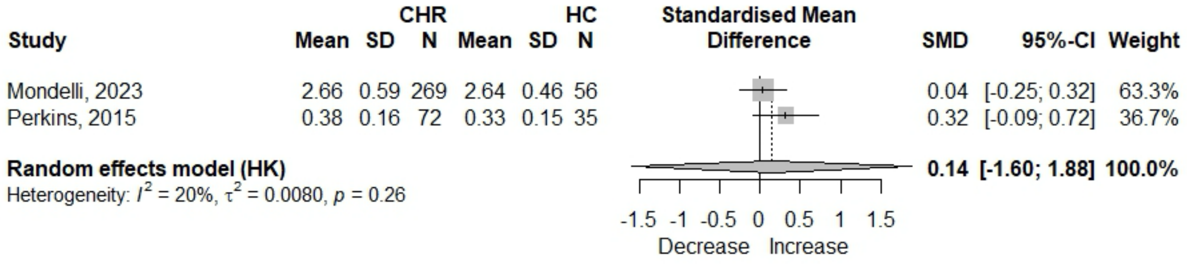

IL-17

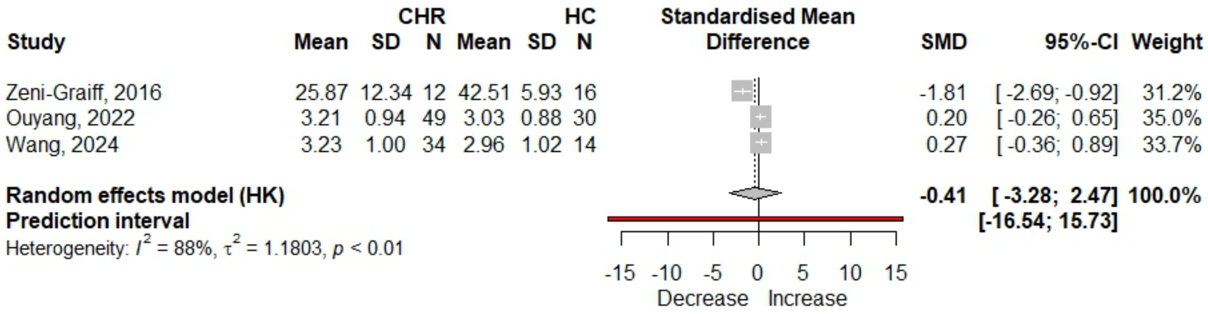

Figure S5. Results of sensitivity analysis tests for pairwise meta-analyses between CHR and HC groups.

IL-2

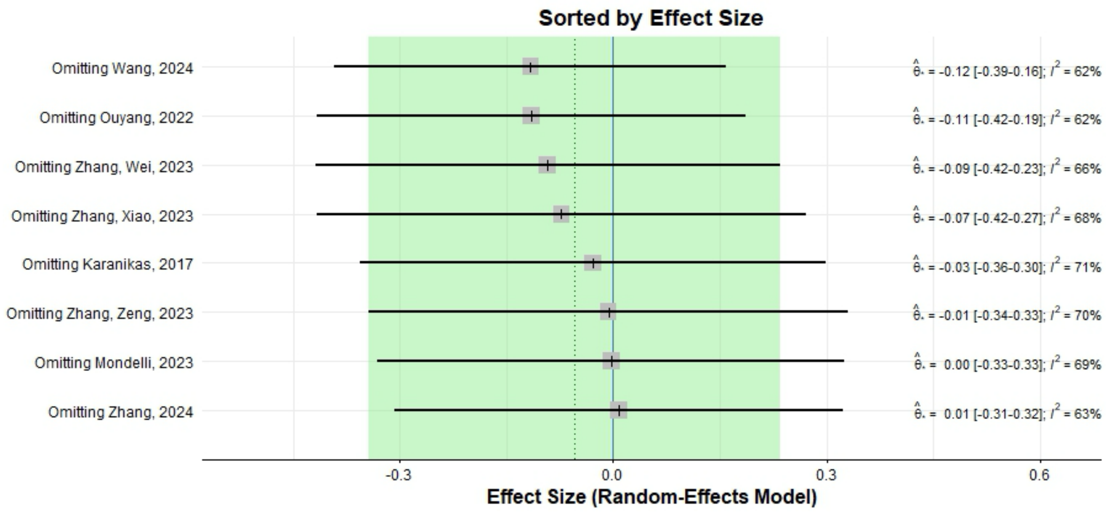

IL-5

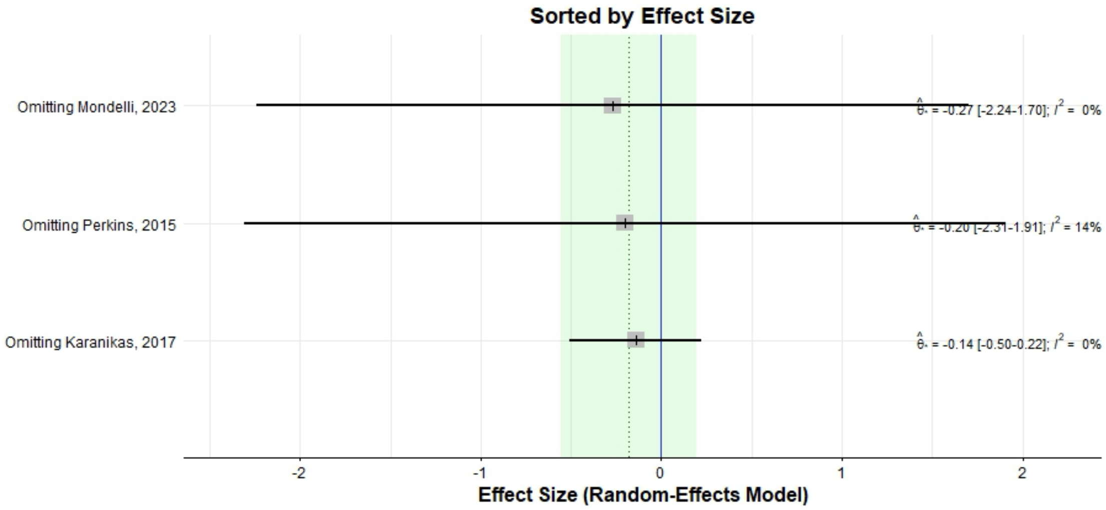

IL-12P70

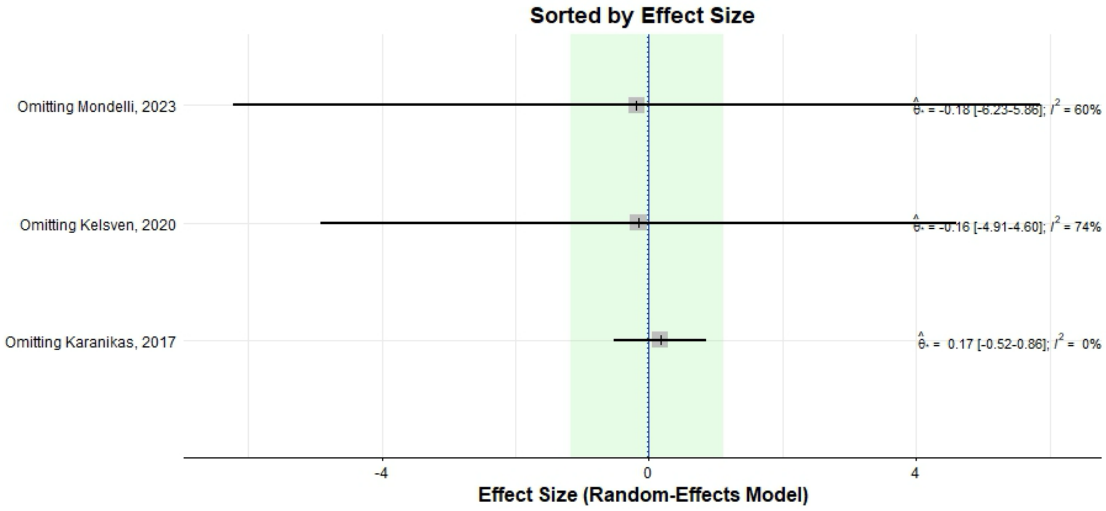

IL-17

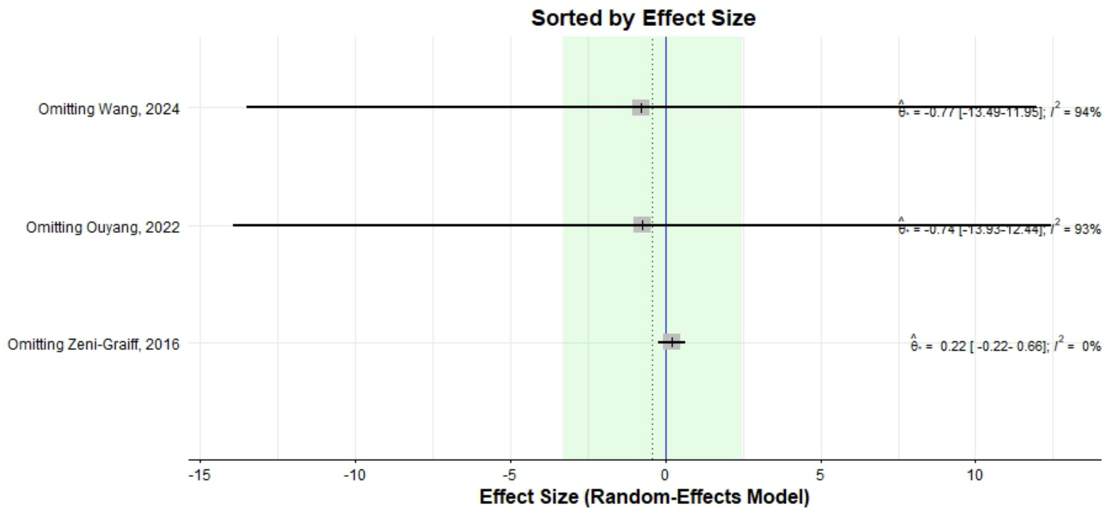

Figure S6. Funnel plots of pairwise analysis of inflammatory factors between CHR and HC groups.

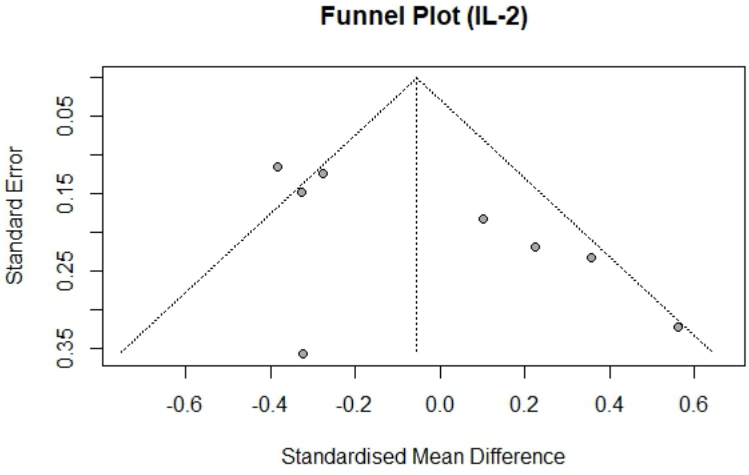

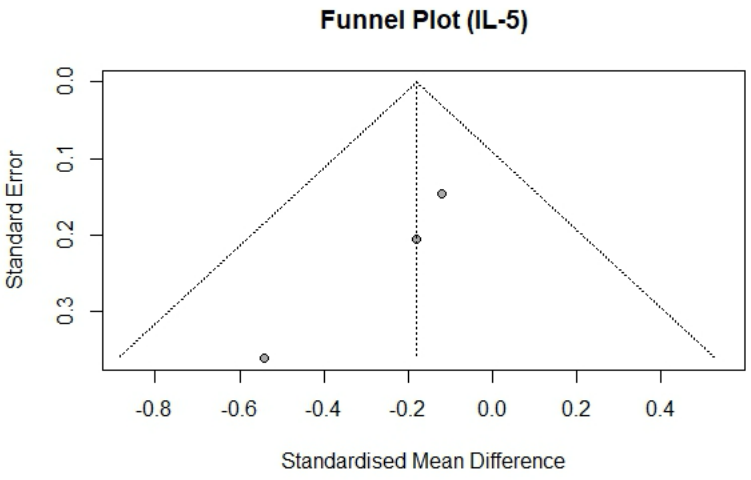
Funnel Plot (IL-5)
Standard Error
0.0
0.1
0.2
0.3
-0.8
-0.6
-0.4
-0.2
0.0
0.2
0.4
Standardised Mean Difference

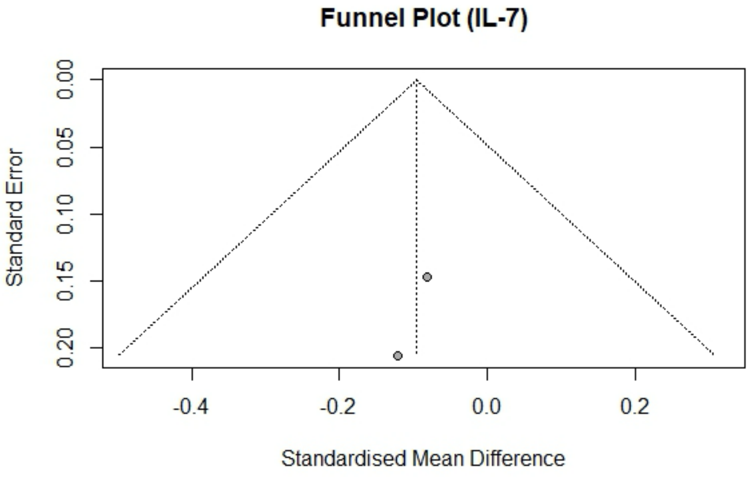
Funnel Plot (IL-7)
Standard Error
0.00
0.05
0.10
0.15
0.20
-0.4
-0.2
0.0
0.2
Standardised Mean Difference

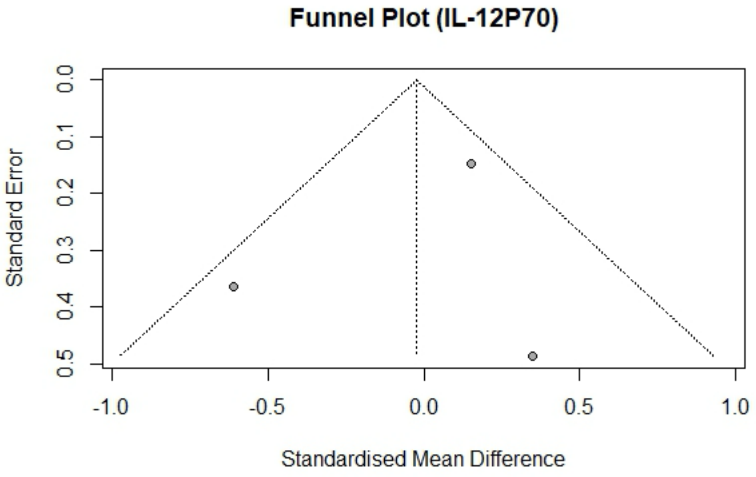
Funnel Plot (IL-12P70)
Standard Error
0.0
0.1
0.2
0.3
0.4
0.5
-1.0
-0.5
0.0
0.5
1.0
Standardised Mean Difference

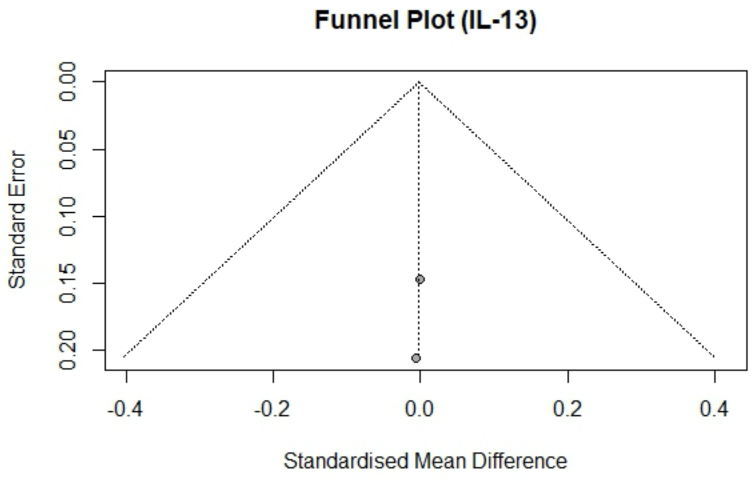

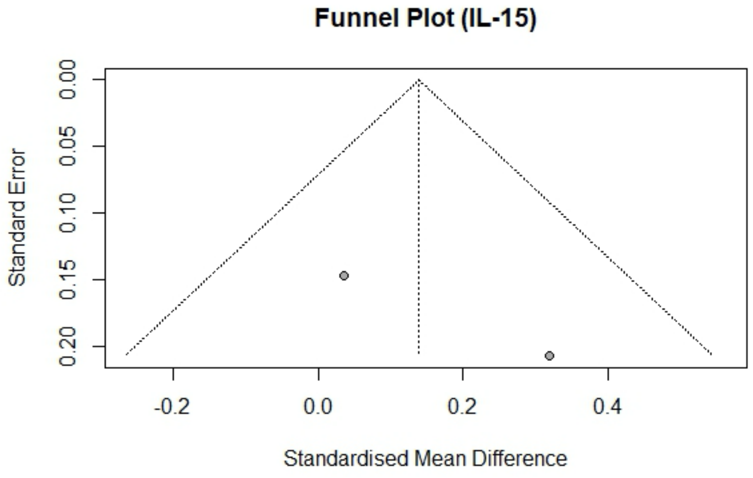

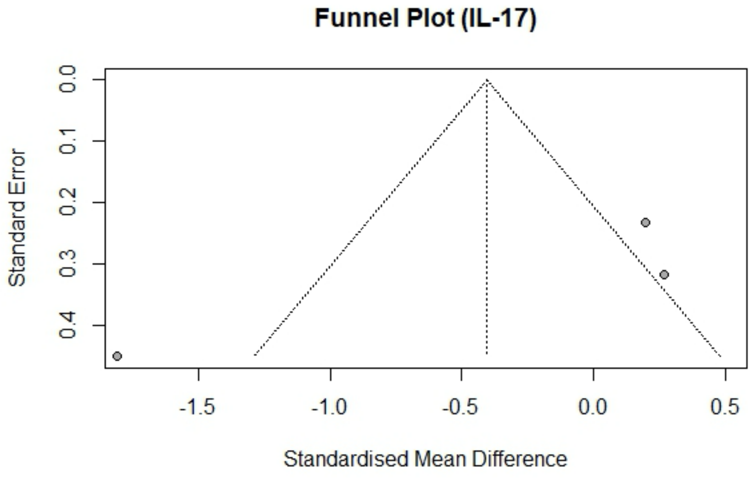

Figure S7. Pairwise meta-analysis results of comparison of makers between CHR-T and CHR-NT groups.

CRP

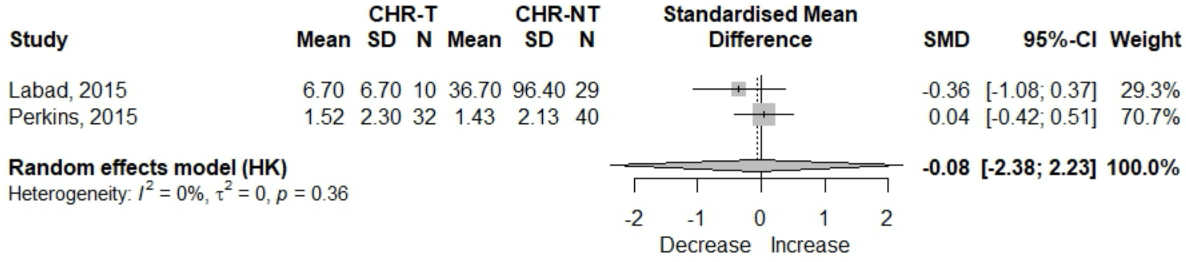

Fibrinogen

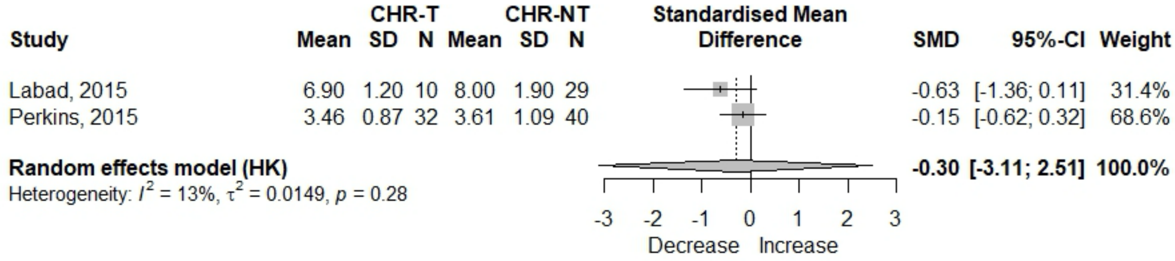

IL-1β

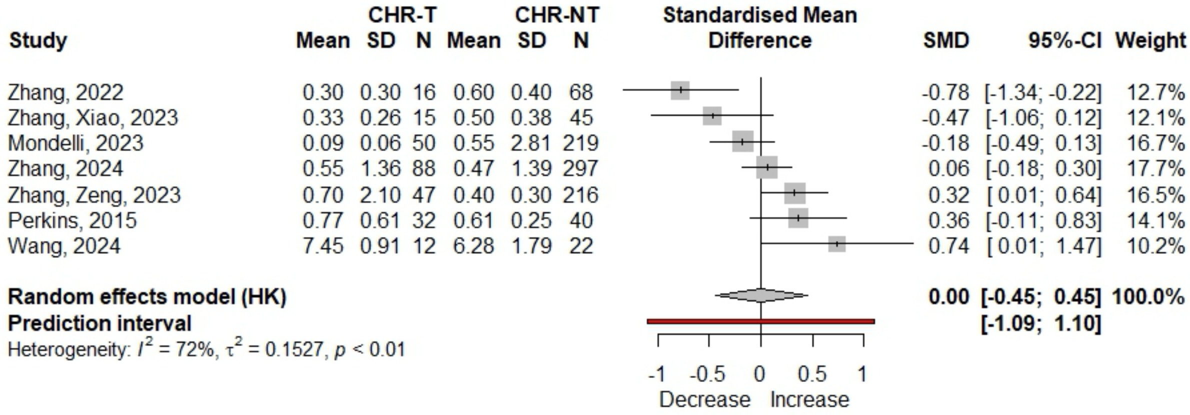

IL-2

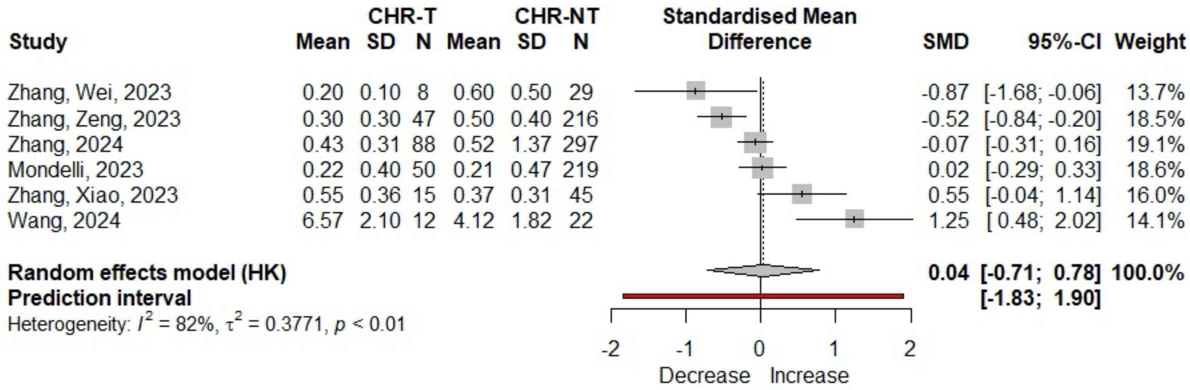

IL-4

Study	CHR-T Mean	CHR-T SD	CHR-T N	CHR-NT Mean	CHR-NT SD	CHR-NT N	SMD	95%-CI	Weight
Wang, 2024	24.91	8.27	12	27.58	5.74	22	-0.39	[-1.10; 0.32]	22.9%
Perkins, 2015	12.93	6.89	32	13.36	6.88	40	-0.06	[-0.53; 0.40]	34.1%
Mondelli, 2023	0.04	0.04	50	0.03	0.02	219	0.40	[0.09; 0.71]	43.0%
**Random effects model (HK)**							**0.06**	**[-0.91; 1.03]**	**100.0%**
**Prediction interval**								**[-4.83; 4.96]**	

Heterogeneity: $I^2 = 63\%$, $\tau^2 = 0.0964$, $p = 0.06$

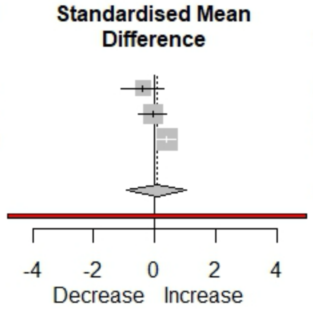

IL-5

Study	CHR-T Mean	CHR-T SD	CHR-T N	CHR-NT Mean	CHR-NT SD	CHR-NT N	SMD	95%-CI	Weight
Perkins, 2015	11.98	10.97	32	10.55	6.86	40	0.16	[-0.31; 0.63]	30.4%
Mondelli, 2023	0.94	0.66	50	0.80	0.68	219	0.21	[-0.10; 0.51]	69.6%
**Random effects model (HK)**							**0.19**	**[-0.08; 0.47]**	**100.0%**

Heterogeneity: $I^2 = 0\%$, $\tau^2 = 0$, $p = 0.87$

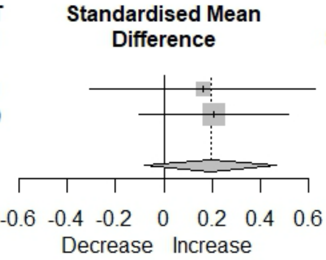

IL-6

Study	CHR-T Mean	CHR-T SD	CHR-T N	CHR-NT Mean	CHR-NT SD	CHR-NT N	SMD	95%-CI	Weight
Zhang, Wei, 2023	0.80	0.40	8	1.40	0.70	29	-0.92	[-1.73; -0.11]	4.2%
Perkins, 2015	1.95	0.98	32	2.25	1.12	40	-0.29	[-0.76; 0.17]	10.8%
Mondelli, 2023	0.48	0.33	50	0.70	0.91	219	-0.26	[-0.57; 0.04]	19.4%
Zhang, Zeng, 2023	1.00	1.40	47	1.20	1.80	216	-0.11	[-0.43; 0.20]	18.8%
Zhang, 2022	1.20	0.70	16	1.50	3.00	68	-0.11	[-0.65; 0.43]	8.4%
Zhang, 2024	1.30	1.33	88	1.25	0.52	297	0.06	[-0.17; 0.30]	25.8%
Zhang, Xiao, 2023	1.49	1.03	15	1.22	0.77	45	0.32	[-0.27; 0.90]	7.4%
Wang, 2024	2.13	0.61	12	1.77	0.62	22	0.57	[-0.15; 1.29]	5.2%
**Random effects model (HK)**							**-0.08**	**[-0.34; 0.17]**	**100.0%**
**Prediction interval**								**[-0.46; 0.29]**	

Heterogeneity: $I^2 = 45\%$, $\tau^2 = 0.0154$, $p = 0.08$

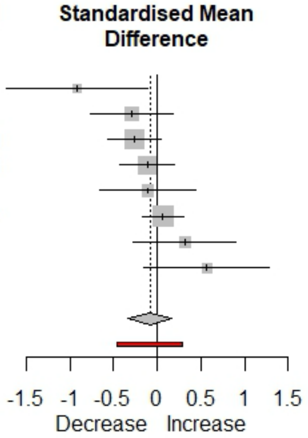

IL-7

Study	CHR-T Mean	CHR-T SD	CHR-T N	CHR-NT Mean	CHR-NT SD	CHR-NT N	SMD	95%-CI	Weight
Mondelli, 2023	14.67	6.07	50	13.46	6.83	219	0.18	[-0.13; 0.49]	69.8%
Perkins, 2015	62.42	45.05	32	51.80	32.94	40	0.27	[-0.19; 0.74]	30.2%
**Random effects model (HK)**							**0.21**	**[-0.34; 0.75]**	**100.0%**

Heterogeneity: $I^2 = 0\%$, $\tau^2 = 0$, $p = 0.74$

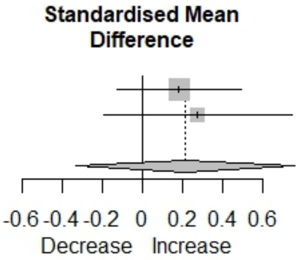

IL-8

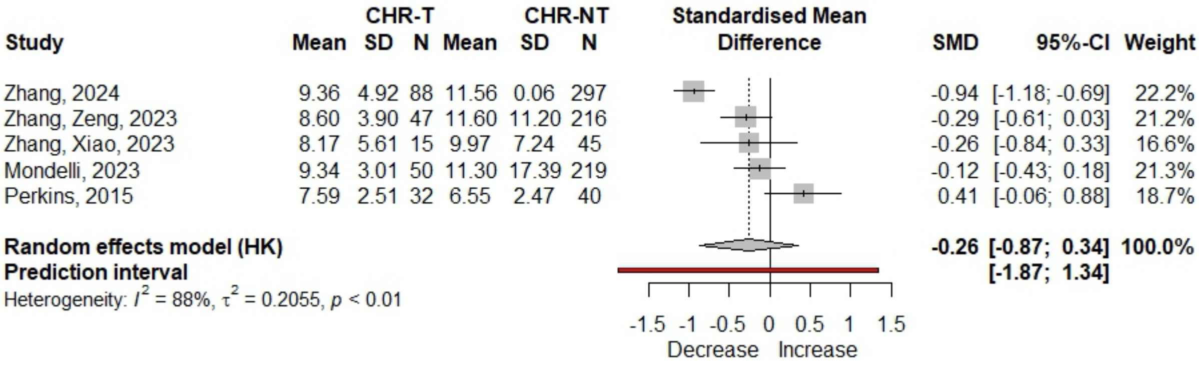

IL-10

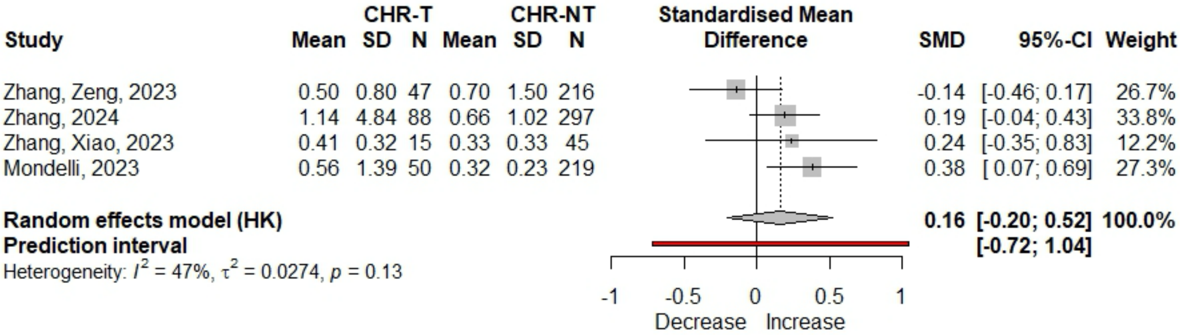

IL-13

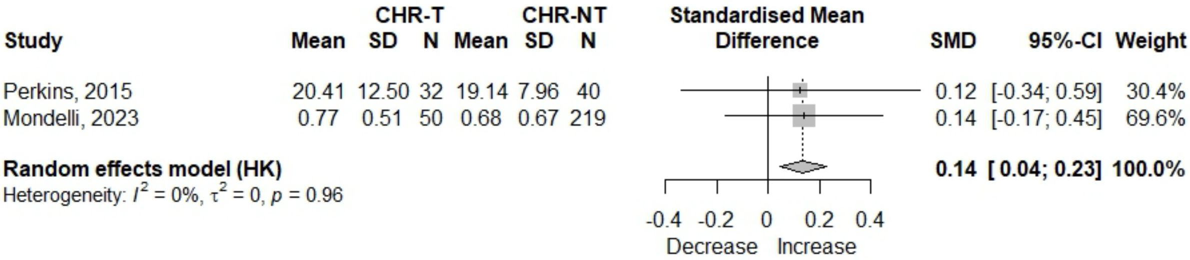

IL-15

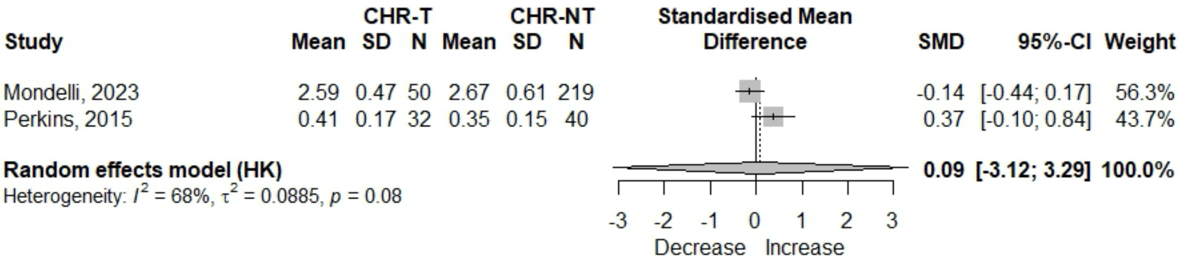

TNF-α

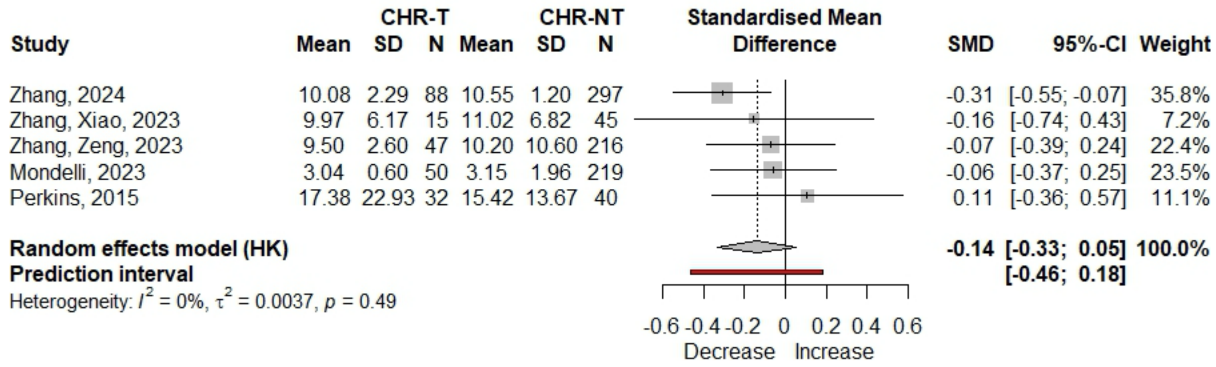

Figure S8. Results of sensitivity analysis tests for pairwise meta-analyses between CHR-T and CHR-NT groups.

IL-1β

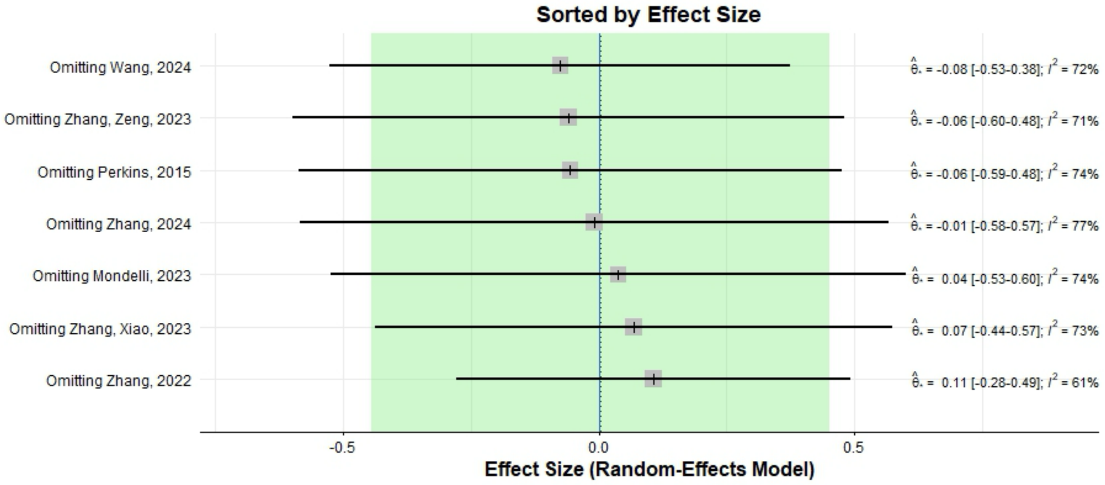

IL-2

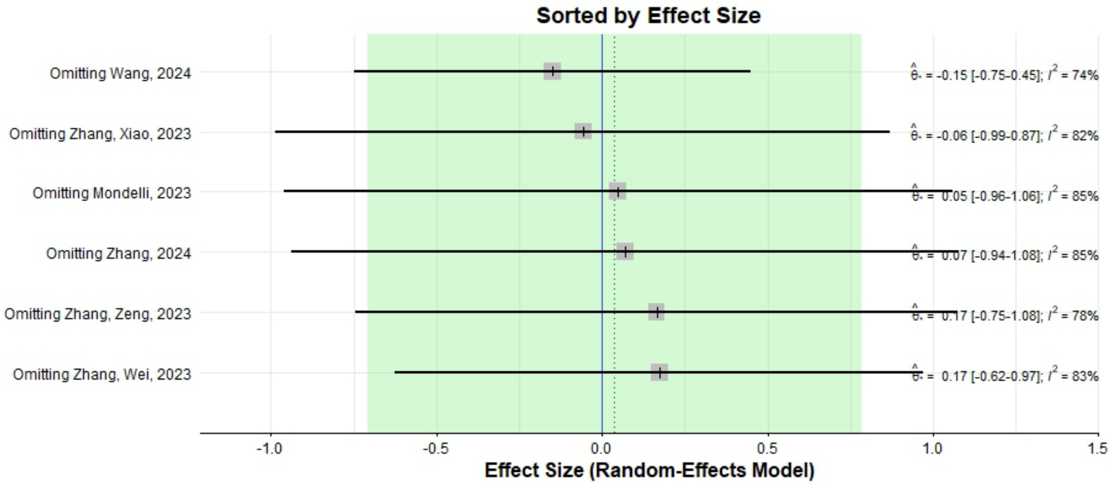

IL-4

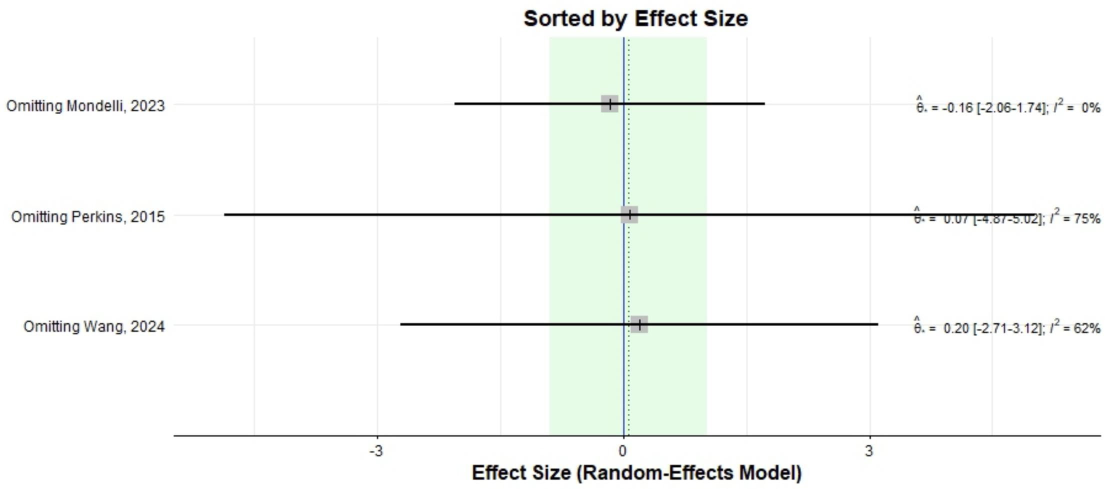

Sorted by Effect Size
Omitting Mondelli, 2023
Omitting Perkins, 2015
Omitting Wang, 2024
$\hat{\theta}$ = -0.16 [-2.06-1.74]; I^2 = 0%
$\hat{\theta}$ = 0.07 [-4.87-5.02]; I^2 = 75%
$\hat{\theta}$ = 0.20 [-2.71-3.12]; I^2 = 62%
-3
0
3
Effect Size (Random-Effects Model)

IL-6

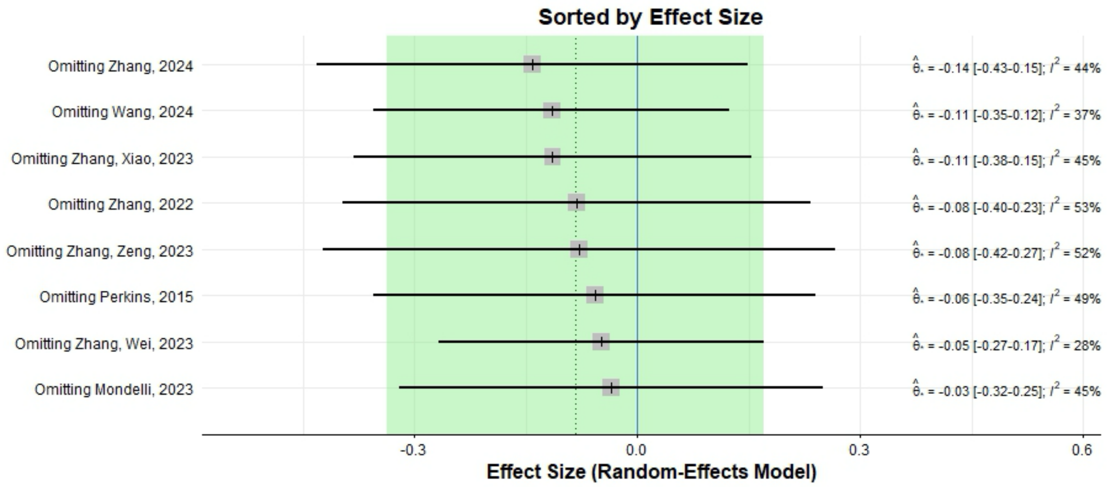

Sorted by Effect Size
Omitting Zhang, 2024
Omitting Wang, 2024
Omitting Zhang, Xiao, 2023
Omitting Zhang, 2022
Omitting Zhang, Zeng, 2023
Omitting Perkins, 2015
Omitting Zhang, Wei, 2023
Omitting Mondelli, 2023
$\hat{\theta}$ = -0.14 [-0.43-0.15]; I^2 = 44%
$\hat{\theta}$ = -0.11 [-0.35-0.12]; I^2 = 37%
$\hat{\theta}$ = -0.11 [-0.38-0.15]; I^2 = 45%
$\hat{\theta}$ = -0.08 [-0.40-0.23]; I^2 = 53%
$\hat{\theta}$ = -0.08 [-0.42-0.27]; I^2 = 52%
$\hat{\theta}$ = -0.06 [-0.35-0.24]; I^2 = 49%
$\hat{\theta}$ = -0.05 [-0.27-0.17]; I^2 = 28%
$\hat{\theta}$ = -0.03 [-0.32-0.25]; I^2 = 45%
-0.3
0.0
0.3
0.6
Effect Size (Random-Effects Model)

IL-8

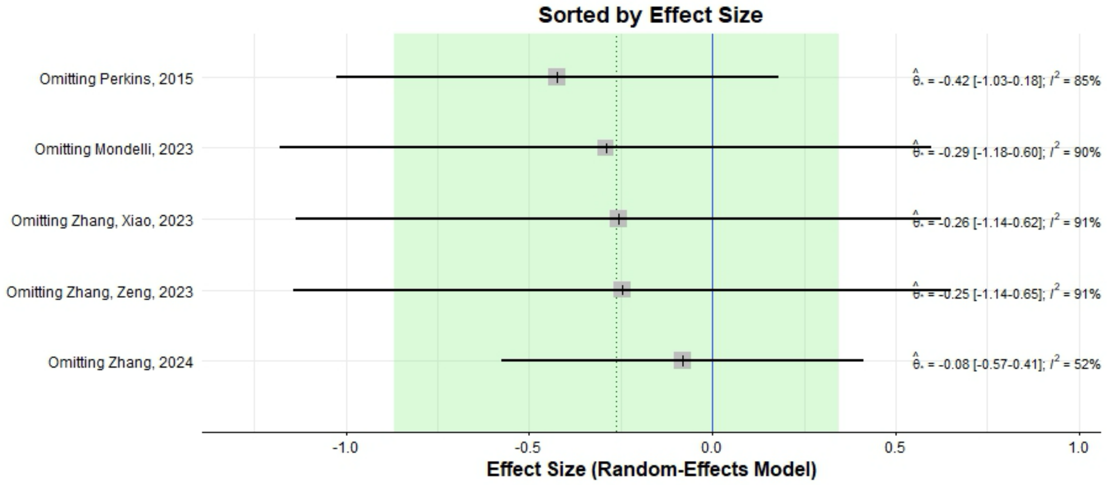

Sorted by Effect Size
Omitting Perkins, 2015
Omitting Mondelli, 2023
Omitting Zhang, Xiao, 2023
Omitting Zhang, Zeng, 2023
Omitting Zhang, 2024
$\hat{\theta}$ = -0.42 [-1.03-0.18]; I^2 = 85%
$\hat{\theta}$ = -0.29 [-1.18-0.60]; I^2 = 90%
$\hat{\theta}$ = -0.26 [-1.14-0.62]; I^2 = 91%
$\hat{\theta}$ = -0.25 [-1.14-0.65]; I^2 = 91%
$\hat{\theta}$ = -0.08 [-0.57-0.41]; I^2 = 52%
-1.0
-0.5
0.0
0.5
1.0
Effect Size (Random-Effects Model)

IL-10

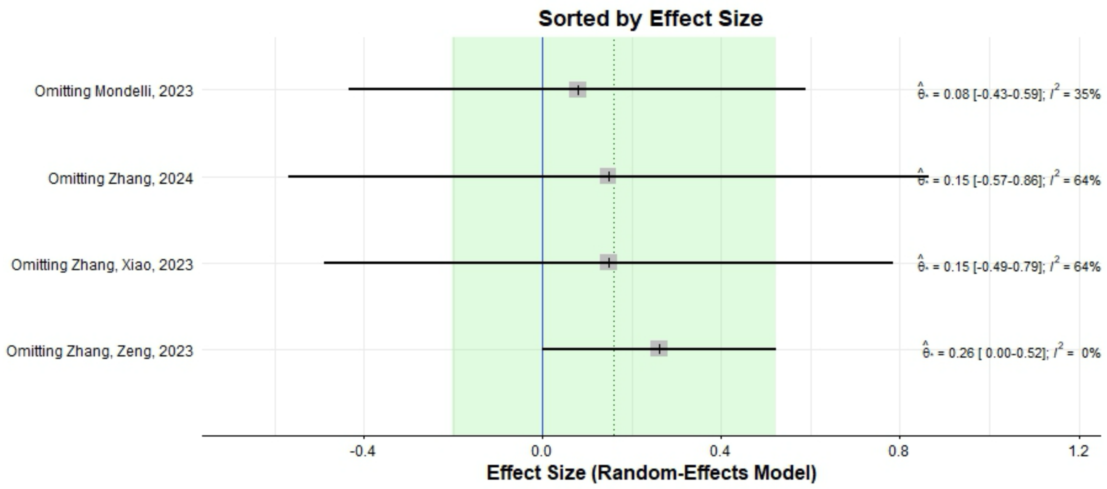

TNF-α

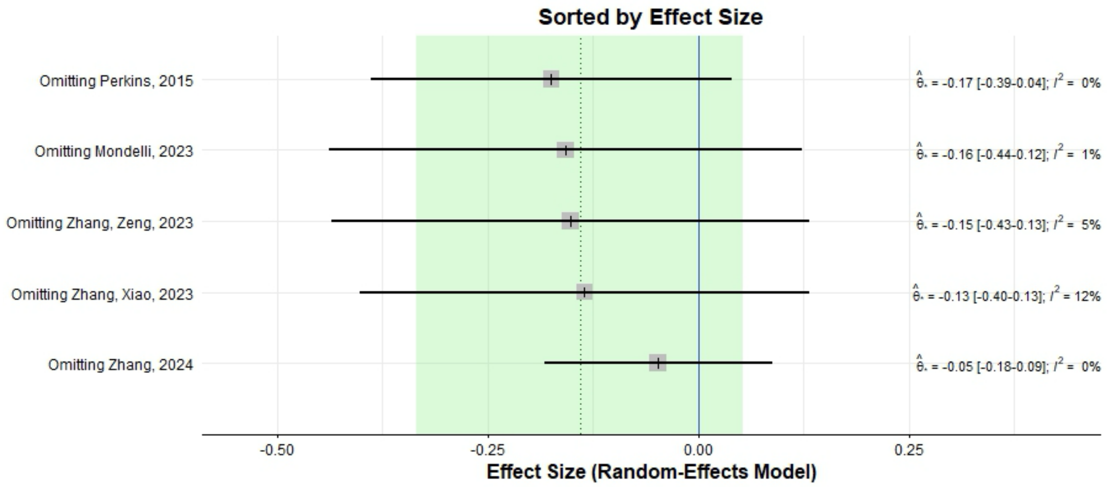

Figure S9. Funnel plots of pairwise analysis of inflammatory factors between CHR-T and CHR-NT groups.

CRP

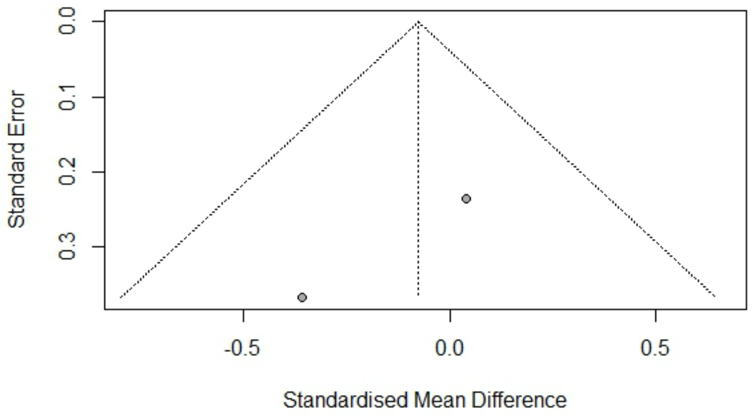

Fibrinogen

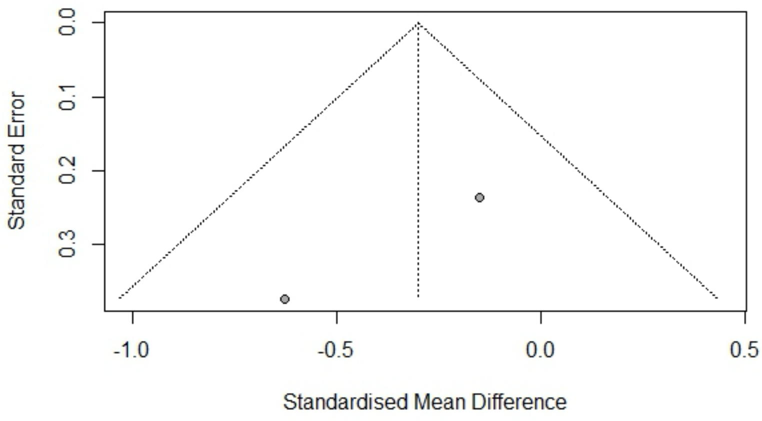

IL-1β

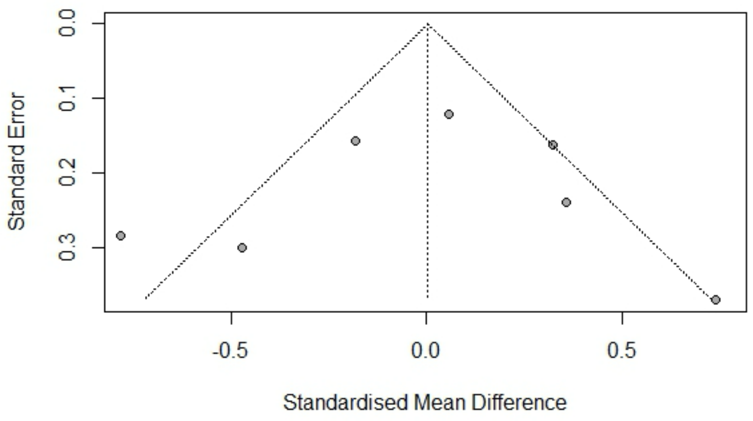

IL-2

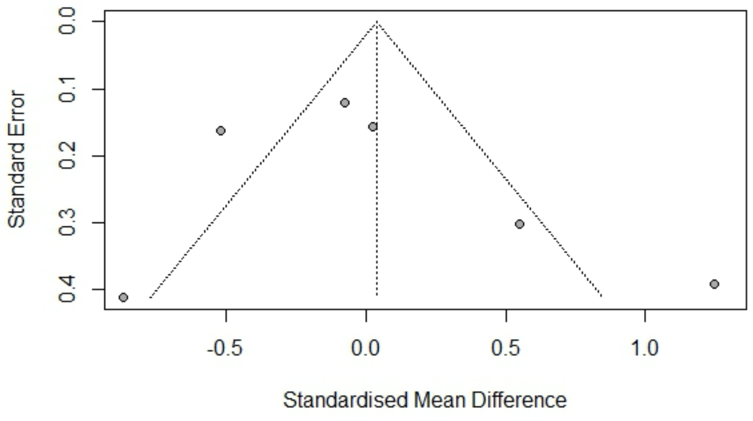

IL-4

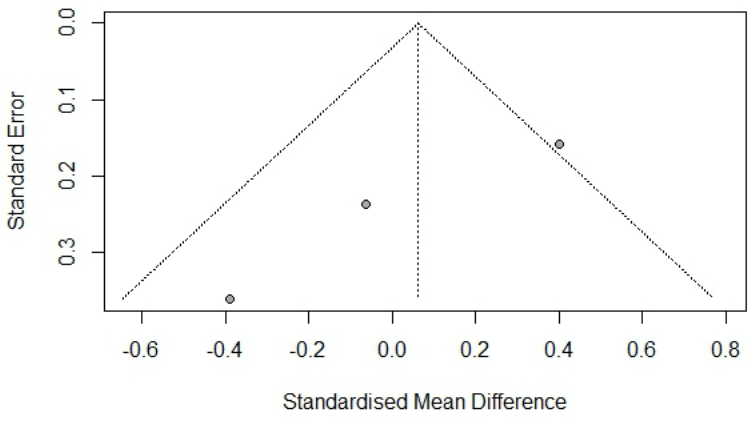

Standard Error
0.0
0.1
0.2
0.3
-0.6
-0.4
-0.2
0.0
0.2
0.4
0.6
0.8
Standardised Mean Difference

IL-5

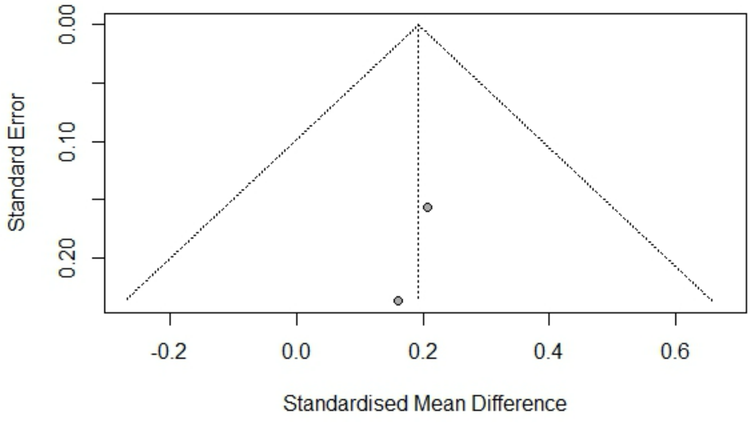

Standard Error
0.00
0.10
0.20
-0.2
0.0
0.2
0.4
0.6
Standardised Mean Difference

IL-6

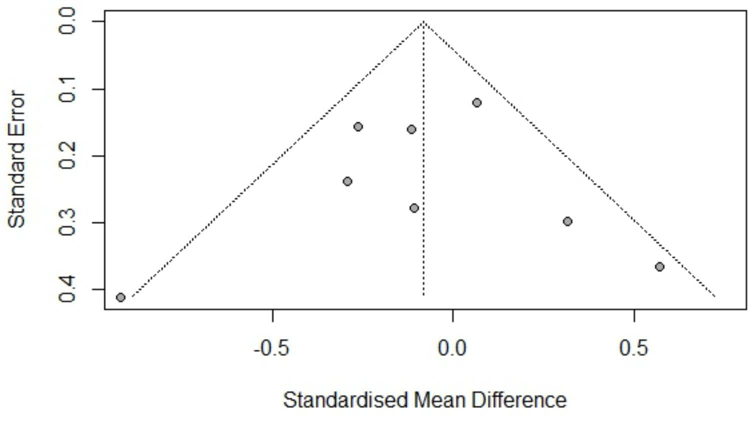

Standard Error
0.0
0.1
0.2
0.3
0.4
-0.5
0.0
0.5
Standardised Mean Difference

IL-7

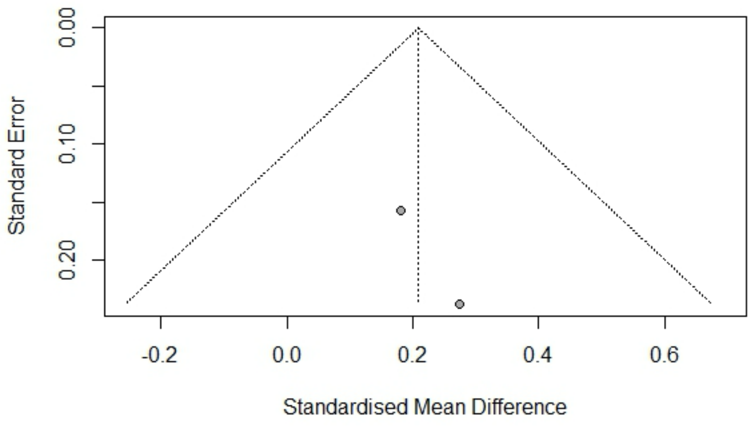

Standard Error
0.00
0.10
0.20
-0.2
0.0
0.2
0.4
0.6
Standardised Mean Difference

IL-8

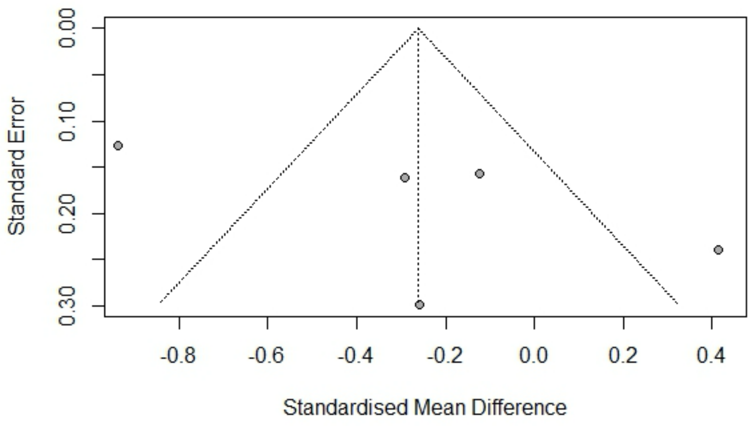

Standard Error
0.00
0.10
0.20
0.30
-0.8
-0.6
-0.4
-0.2
0.0
0.2
0.4
Standardised Mean Difference

IL-10

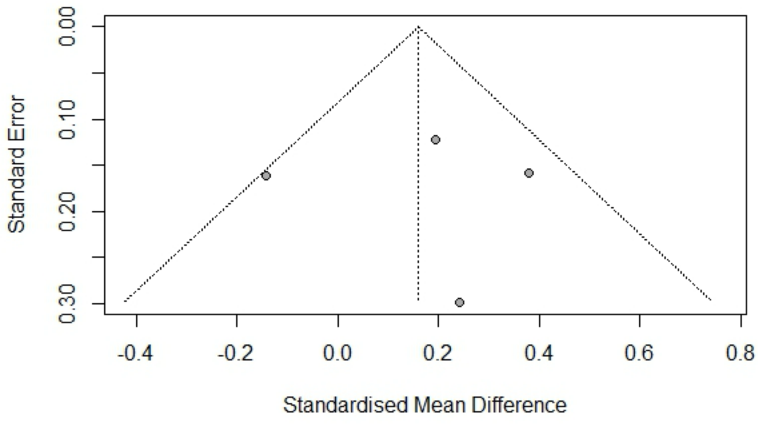

Standard Error
0.00
0.10
0.20
0.30
-0.4
-0.2
0.0
0.2
0.4
0.6
0.8
Standardised Mean Difference

IL-13

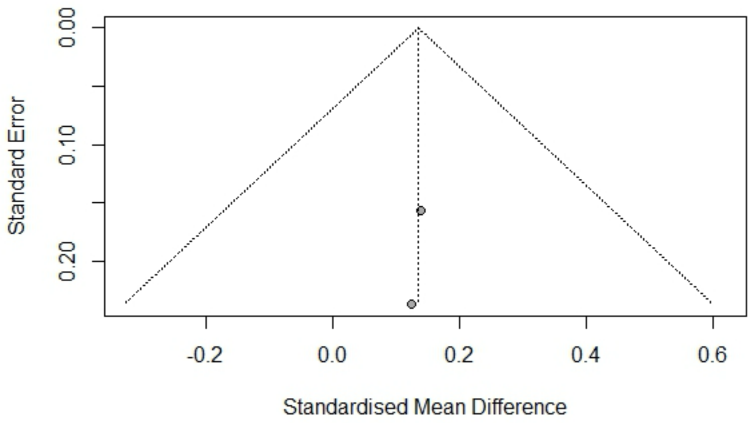

IL-15

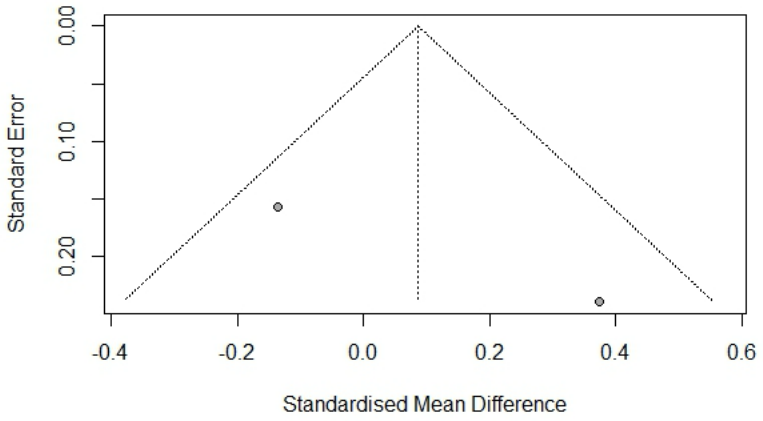

TNF-α

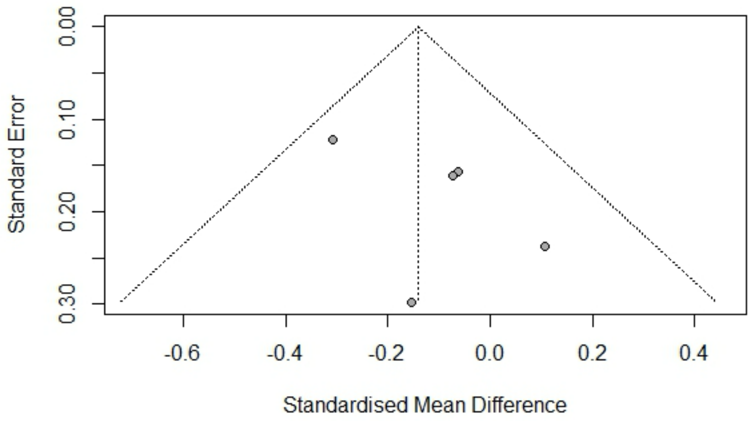

References for included studies (numbered as per table S4 & S5):

Arolt, V., Weitzsch, C., Wilke, I., Nolte, A., Pinnow, M., Rothermundt, M., & Kirchner, H. (1997). Production of interferon-gamma in families with multiple occurrence of schizophrenia. *Psychiatry Research, 66*(2-3), 145-152. Retrieved from https://pubmed.ncbi.nlm.nih.gov/9075278

Chouinard, V.-A., Henderson, D. C., Dalla Man, C., Valeri, L., Gray, B. E., Ryan, K. P., . . . Öngür, D. (2019). Impaired insulin signaling in unaffected siblings and patients with first-episode psychosis. *Molecular Psychiatry, 24*(10), 1513-1522. doi:10.1038/s41380-018-0045-1

Corsi-Zuelli, F., Loureiro, C. M., Shuhama, R., Fachim, H. A., Menezes, P. R., Louzada-Junior, P., . . . Del-Ben, C. M. (2020). Cytokine profile in first-episode psychosis, unaffected siblings and community-based controls: the effects of familial liability and childhood maltreatment. *Psychological Medicine, 50*(7), 1139-1147. doi:10.1017/S0033291719001016

Corsi-Zuelli, F., Quattrone, D., Ragazzi, T. C. C., Loureiro, C. M., Shuhama, R., Menezes, P. R., . . . Del-Ben, C. M. (2024). Transdiagnostic dimensions of symptoms and experiences associated with immune proteins in the continuity of psychosis. *Psychological Medicine, 54*(9), 2099-2111. doi:10.1017/S0033291724000199

Corsi-Zuelli, F., Schneider, A. H., Santos-Silva, T., Loureiro, C. M., Shuhama, R., Menezes, P. R., . . . Del-Ben, C. M. (2022). Increased blood neutrophil extracellular traps (NETs) associated with early life stress: translational findings in recent-onset schizophrenia and rodent model. *Translational Psychiatry, 12*(1), 526. doi:10.1038/s41398-022-02291-4

Delaney, S., Fallon, B., Alaedini, A., Yolken, R., Indart, A., Feng, T., . . . Javitt, D. (2019). Inflammatory biomarkers in psychosis and clinical high risk populations. *Schizophrenia Research, 206*, 440-443. doi:10.1016/j.schres.2018.10.017

Gallart-Palau, X., Muntané, G., Martorell, L., Amigó, N., Correig, X., Ribalta, J., . . . Vilella, E. (2023). Gradual Increase in Inflammation-Linked Glycoproteins and a Proatherogenic Lipoprotein Profile in the Early Stages of Psychosis as Characterized by 1H NMR Blood Analysis. *Journal of Proteome Research, 22*(7), 2271-2280. doi:10.1021/acs.jproteome.2c00847

Gaughran, F., O'Neill, E., Sham, P., Daly, R. J., & Shanahan, F. (2002). Soluble interleukin 2 receptor levels in families of people with schizophrenia. *Schizophrenia Research, 56*(3), 235-239. Retrieved from https://pubmed.ncbi.nlm.nih.gov/12072172

Kelsven, S., de la Fuente-Sandoval, C., Achim, C. L., Reyes-Madrigal, F., Mirzakhanian, H., Domingues, I., & Cadenhead, K. (2020). Immuno-inflammatory changes across phases of early psychosis: The impact of antipsychotic medication and stage of illness. *Schizophrenia Research, 226*, 13-23. doi:10.1016/j.schres.2020.01.003

Labad, J., Stojanovic-Pérez, A., Montalvo, I., Solé, M., Cabezas, Á., Ortega, L., . . . Gutiérrez-Zotes, A. (2015). Stress biomarkers as predictors of transition to psychosis in at-risk mental states: roles for cortisol, prolactin and albumin.

Journal of Psychiatric Research, 60, 163-169. doi:10.1016/j.jpsychires.2014.10.011

Lizano, P. L., Keshavan, M. S., Tandon, N., Mathew, I. T., Mothi, S. S., Montrose, D. M., & Yao, J. K. (2016). Angiogenic and immune signatures in plasma of young relatives at familial high-risk for psychosis and first-episode patients: A preliminary study. *Schizophrenia Research, 170*(1), 115-122. doi:10.1016/j.schres.2015.12.001

Mondelli, V., Blackman, G., Kempton, M. J., Pollak, T. A., Iyegbe, C., Valmaggia, L. R., . . . McGuire, P. (2023). Serum immune markers and transition to psychosis in individuals at clinical high risk. *Brain, Behavior, and Immunity, 110*, 290-296. doi:10.1016/j.bbi.2023.03.014

Moreno, I., Stojanovic-Pérez, A., Bulduk, B., Sánchez-Gistau, V., Algora, M. J., Ortega, L., . . . Martorell, L. (2023). High blood levels of brain-derived neurotrophic factor (BDNF) mRNA in early psychosis are associated with inflammatory markers. *Journal of Psychiatric Research, 164*, 440-446. doi:10.1016/j.jpsychires.2023.07.003

Noyan, H., Erdağ, E., Tüzün, E., Yaylım, İ., Küçükhüseyin, Ö., Hakan, M. T., . . . Üçok, A. (2021). Association of the kynurenine pathway metabolites with clinical, cognitive features and IL-1β levels in patients with schizophrenia spectrum disorder and their siblings. *Schizophrenia Research, 229*, 27-37. doi:10.1016/j.schres.2021.01.014

Ntouros, E., Karanikas, E., Floros, G., Andreou, C., Tsoura, A., Garyfallos, G., & Bozikas, V. P. (2018). Social cognition in the course of psychosis and its correlation with biomarkers in a male cohort. *Cognitive Neuropsychiatry, 23*(2), 103-115. doi:10.1080/13546805.2018.1440201

Nunes, S. O. V., Matsuo, T., Kaminami, M. S., Watanabe, M. A. E., Reiche, E. M. V., & Itano, E. N. (2006). An autoimmune or an inflammatory process in patients with schizophrenia, schizoaffective disorder, and in their biological relatives. *Schizophrenia Research, 84*(1), 180-182. Retrieved from https://pubmed.ncbi.nlm.nih.gov/16530388

Ouyang, L., Li, D., Li, Z., Ma, X., Yuan, L., Fan, L., . . . Chen, X. (2022). IL-17 and TNF-β: Predictive biomarkers for transition to psychosis in ultra-high risk individuals. *Frontiers In Psychiatry, 13*, 1072380. doi:10.3389/fpsyt.2022.1072380

Perkins, D. O., Jeffries, C. D., Addington, J., Bearden, C. E., Cadenhead, K. S., Cannon, T. D., . . . Heinssen, R. (2015). Towards a psychosis risk blood diagnostic for persons experiencing high-risk symptoms: preliminary results from the NAPLS project. *Schizophrenia Bulletin, 41*(2), 419-428. doi:10.1093/schbul/sbu099

Piotrowski, P., Kotowicz, K., Rymaszewska, J., Beszłej, J. A., Plichta, P., Samochowiec, J., . . . Misiak, B. (2019). Allostatic load index and its clinical correlates at various stages of psychosis. *Schizophrenia Research, 210*, 73-80. doi:10.1016/j.schres.2019.06.009

Rebouças, D. B., Rabelo-da-Ponte, F. D., Massuda, R., Czepielewski, L. S., & Gama, C. S. (2018). The Relationship between Cytokines and Verbal Memory in

Individuals with Schizophrenia and Their Unaffected Siblings. *Neuroimmunomodulation, 25*(5-6), 334-339. doi:10.1159/000492716

Stojanovic, A., Martorell, L., Montalvo, I., Ortega, L., Monseny, R., Vilella, E., & Labad, J. (2014). Increased serum interleukin-6 levels in early stages of psychosis: associations with at-risk mental states and the severity of psychotic symptoms. *Psychoneuroendocrinology, 41*, 23-32. doi:10.1016/j.psyneuen.2013.12.005

Wang, Y., Fan, L., He, Y., Yuan, L., Li, Z., Zheng, W., . . . Ma, X. (2024). Compensatory thickening of cortical thickness in early stage of schizophrenia. *Cerebral Cortex (New York, N.Y. : 1991), 34*(6). doi:10.1093/cercor/bhae255

Wang, Y., Wei, Y., Edmiston, E. K., Womer, F. Y., Zhang, X., Duan, J., . . . Wang, F. (2020). Altered structural connectivity and cytokine levels in Schizophrenia and Genetic high-risk individuals: Associations with disease states and vulnerability. *Schizophrenia Research, 223*, 158-165. doi:10.1016/j.schres.2020.05.044

Yüksel, R. N., Göverti, D., Kahve, A. C., Çakmak, I. B., Yücel, Ç., & Göka, E. (2020). Galectin-1 and Galectin-3 Levels in Patients with Schizophrenia and their Unaffected Siblings. *The Psychiatric Quarterly, 91*(3), 715-725. doi:10.1007/s11126-020-09731-8

Zeni-Graiff, M., Rizzo, L. B., Mansur, R. B., Maurya, P. K., Sethi, S., Cunha, G. R., . . . Brietzke, E. (2016). Peripheral immuno-inflammatory abnormalities in ultra-high risk of developing psychosis. *Schizophrenia Research, 176*(2-3), 191-195. doi:10.1016/j.schres.2016.06.031

Zhang, T., Wei, Y., Zeng, J., Ye, J., Tang, X., Xu, L., . . . Wang, J. (2023). Interleukin-2/interleukin-6 imbalance correlates with conversion to psychosis from a clinical high-risk state. *Psychiatry and Clinical Neurosciences, 77*(1), 62-63. doi:10.1111/pcn.13476

Zhang, T., Xiao, X., Wu, H., Zeng, J., Ye, J., Gao, Y., . . . Wang, J. (2023). Association of Attenuated Niacin Response With Inflammatory Imbalance and Prediction of Conversion to Psychosis From Clinical High-risk Stage. *The Journal of Clinical Psychiatry, 84*(5). doi:10.4088/JCP.22m14731

Zhang, T., Zeng, J., Wei, Y., Ye, J., Tang, X., Xu, L., . . . Wang, J. (2022). Changes in inflammatory balance correlates with conversion to psychosis among individuals at clinical high-risk: A prospective cohort study. *Psychiatry Research, 318*, 114938. doi:10.1016/j.psychres.2022.114938

Zhang, T., Zeng, J., Wei, Y., Ye, J., Tang, X., Xu, L., . . . Wang, J. (2023). Changes in Inflammatory Markers in Clinical High Risk of Developing Psychosis. *Neuropsychobiology, 82*(2), 104-116. doi:10.1159/000528770

Zhang, T. H., Chen, X., Wei, Y. Y., Tang, X. C., Xu, L. H., Cui, H. R., . . . Wang, J. J. (2024). Associations between cytokine levels and cognitive function among individuals at clinical high risk for psychosis. *Progress In Neuro-psychopharmacology & Biological Psychiatry, 136*, 111166. doi:10.1016/j.pnpbp.2024.111166

PRISMA 2020 checklist

Template downloaded from: https://www.prisma-statement.org/

Section and Topic	Item #	Checklist item	Location where item is reported
TITLE			
Title	1	Identify the report as a systematic review.	Page 1
ABSTRACT			
Abstract	2	See the PRISMA 2020 for Abstracts checklist.	Page 2
INTRODUCTION			
Rationale	3	Describe the rationale for the review in the context of existing knowledge.	Page 3-5
Objectives	4	Provide an explicit statement of the objective(s) or question(s) the review addresses.	Page 5-6
METHODS			
Eligibility criteria	5	Specify the inclusion and exclusion criteria for the review and how studies were grouped for the syntheses.	Page 6-7
Information sources	6	Specify all databases, registers, websites, organisations, reference lists and other sources searched or consulted to identify studies. Specify the date when each source was last searched or consulted.	Page 6
Search strategy	7	Present the full search strategies for all databases, registers and websites, including any filters and limits used.	Supplementary material page 2-3
Selection process	8	Specify the methods used to decide whether a study met the inclusion criteria of the review, including how many reviewers screened each record and each report retrieved, whether they worked independently, and if applicable, details of automation tools used in the process.	Page 7
Data collection process	9	Specify the methods used to collect data from reports, including how many reviewers collected data from each report, whether they worked independently, any processes for obtaining or confirming data from study investigators, and if applicable, details of automation tools used in the process.	page 7-8
Data items	10a	List and define all outcomes for which data were sought. Specify whether all results that were	Page 8

		compatible with each outcome domain in each study were sought (e.g. for all measures, time points, analyses), and if not, the methods used to decide which results to collect.	
	10 b	List and define all other variables for which data were sought (e.g. participant and intervention characteristics, funding sources). Describe any assumptions made about any missing or unclear information.	Page 8-9
Study risk of bias assessment	11	Specify the methods used to assess risk of bias in the included studies, including details of the tool(s) used, how many reviewers assessed each study and whether they worked independently, and if applicable, details of automation tools used in the process.	Page 7-8
			Supplementary material page 3-4
Effect measures	12	Specify for each outcome the effect measure(s) (e.g. risk ratio, mean difference) used in the synthesis or presentation of results.	Page 9-11
Synthesis methods	13a	Describe the processes used to decide which studies were eligible for each synthesis (e.g. tabulating the study intervention characteristics and comparing against the planned groups for each synthesis (item #5)).	Page 6-7
	13 b	Describe any methods required to prepare the data for presentation or synthesis, such as handling of missing summary statistics, or data conversions.	Page 8-9
	13c	Describe any methods used to tabulate or visually display results of individual studies and syntheses.	page 8-9
	13 d	Describe any methods used to synthesize results and provide a rationale for the choice(s). If meta-analysis was performed, describe the model(s), method(s) to identify the presence and extent of statistical heterogeneity, and software package(s) used.	Page 9-11
	13e	Describe any methods used to explore possible causes of heterogeneity among study results (e.g. subgroup analysis, meta-regression).	Page 9-10
	13f	Describe any sensitivity analyses conducted to assess robustness of the synthesized results.	Page 10
Reporting bias	14	Describe any methods used to assess risk of bias due to missing results in a synthesis (arising from	Page 7-8

assessment		reporting biases).	
Certainty assessment	15	Describe any methods used to assess certainty (or confidence) in the body of evidence for an outcome.	Page 9-10
RESULTS			
Study selection	16a	Describe the results of the search and selection process, from the number of records identified in the search to the number of studies included in the review, ideally using a flow diagram.	Page 11
			Figure 1
	16 b	Cite studies that might appear to meet the inclusion criteria, but which were excluded, and explain why they were excluded.	Supplement ary material page 17
Study characteris tics	17	Cite each included study and present its characteristics.	Page 11-12
			Table 1
			Supplement ary material page 5-14
Risk of bias in studies	18	Present assessments of risk of bias for each included study.	Supplement ary material page 15-16
Results of individual studies	19	For all outcomes, present, for each study: (a) summary statistics for each group (where appropriate) and (b) an effect estimate and its precision (e.g. confidence/credible interval), ideally using structured tables or plots.	Figure 2-3
			Table 2
Results of syntheses	20a	For each synthesis, briefly summarise the characteristics and risk of bias among contributing studies.	Page 11-13
	20 b	Present results of all statistical syntheses conducted. If meta-analysis was done, present for each the summary estimate and its precision (e.g. confidence/credible interval) and measures of statistical heterogeneity. If comparing groups, describe the direction of the effect.	Page 12-13
	20c	Present results of all investigations of possible causes of heterogeneity among study results.	Page 13
	20 d	Present results of all sensitivity analyses conducted to assess the robustness of the synthesized results.	Page 14
Reporting	21	Present assessments of risk of bias due to missing	Page 13-14

biases		results (arising from reporting biases) for each synthesis assessed.	
Certainty of evidence	22	Present assessments of certainty (or confidence) in the body of evidence for each outcome assessed.	Table 2
DISCUSSION			
Discussion	23a	Provide a general interpretation of the results in the context of other evidence.	Page 14
	23 b	Discuss any limitations of the evidence included in the review.	Page 15-20
	23c	Discuss any limitations of the review processes used.	Page 19-20
	23 d	Discuss implications of the results for practice, policy, and future research.	Page 16-21
OTHER INFORMATION			
Registration and protocol	24a	Provide registration information for the review, including register name and registration number, or state that the review was not registered.	Page 6
	24 b	Indicate where the review protocol can be accessed, or state that a protocol was not prepared.	Page 6
	24c	Describe and explain any amendments to information provided at registration or in the protocol.	Page 6
Support	25	Describe sources of financial or non-financial support for the review, and the role of the funders or sponsors in the review.	Page 22
Competing interests	26	Declare any competing interests of review authors.	Page 22
Availability of data, code and other materials	27	Report which of the following are publicly available and where they can be found: template data collection forms; data extracted from included studies; data used for all analyses; analytic code; any other materials used in the review.	Page 22
